# Repurposing anti-inflammasome NRTIs for improving insulin sensitivity and reducing type 2 diabetes development

**DOI:** 10.1038/s41467-020-18528-z

**Published:** 2020-09-23

**Authors:** Jayakrishna Ambati, Joseph Magagnoli, Hannah Leung, Shao-bin Wang, Chris A. Andrews, Dongxu Fu, Akshat Pandey, Srabani Sahu, Siddharth Narendran, Shuichiro Hirahara, Shinichi Fukuda, Jian Sun, Lekha Pandya, Meenakshi Ambati, Felipe Pereira, Akhil Varshney, Tammy Cummings, James W. Hardin, Babatunde Edun, Charles L. Bennett, Kameshwari Ambati, Benjamin J. Fowler, Nagaraj Kerur, Christian Röver, Norbert Leitinger, Brian C. Werner, Joshua D. Stein, S. Scott Sutton, Bradley D. Gelfand

**Affiliations:** 1 Center for Advanced Vision Science, University of Virginia School of Medicine, Charlottesville, VA USA; 2 Department of Ophthalmology, University of Virginia School of Medicine, Charlottesville, VA USA; 3 Department of Pathology, University of Virginia School of Medicine, Charlottesville, VA USA; 4 Department of Microbiology, Immunology, and Cancer Biology, University of Virginia School of Medicine, Charlottesville, VA USA; 5 Dorn Research Institute, Columbia VA Health Care System, Columbia, SC USA; 6 Department of Clinical Pharmacy & Outcomes Sciences, College of Pharmacy, University of South Carolina, Columbia, SC USA; 7 Department of Ophthalmology and Visual Sciences, University of Michigan Medical School, Ann Arbor, MI USA; 8 Center for Eye Policy and Innovation, University of Michigan, Ann Arbor, MI USA; 9 Department of Pharmacology, University of Virginia School of Medicine, Charlottesville, VA USA; 10 Department of Ophthalmology, University of Tsukuba, Ibaraki, Japan; 11 Department of Epidemiology & Biostatistics, University of South Carolina, Columbia, SC USA; 12 Center for Medication Safety and Efficacy, College of Pharmacy, University of South Carolina, Columbia, SC USA; 13 Department of Ophthalmology and Visual Sciences, University of Kentucky, Lexington, KY USA; 14 Department of Neuroscience, University of Virginia School of Medicine, Charlottesville, VA USA; 15 Department of Medical Statistics, University Medical Center Göttingen, Göttingen, Germany; 16 Department of Orthopaedics, University of Virginia School of Medicine, Charlottesville, VA USA; 17 Department of Health Management and Policy, University of Michigan School of Public Health, Ann Arbor, MI USA; 18 Department of Biomedical Engineering, University of Virginia School of Medicine, Charlottesville, VA USA; 19 Present Address: Department of Medicine, Baystate Medical Center, Springfield, MA USA

**Keywords:** Chronic inflammation, Type 2 diabetes, Preclinical research, Inflammasome

## Abstract

Innate immune signaling through the NLRP3 inflammasome is activated by multiple diabetes-related stressors, but whether targeting the inflammasome is beneficial for diabetes is still unclear. Nucleoside reverse-transcriptase inhibitors (NRTI), drugs approved to treat HIV-1 and hepatitis B infections, also block inflammasome activation. Here, we show, by analyzing five health insurance databases, that the adjusted risk of incident diabetes is 33% lower in patients with NRTI exposure among 128,861 patients with HIV-1 or hepatitis B (adjusted hazard ratio for NRTI exposure, 0.673; 95% confidence interval, 0.638 to 0.710; P < 0.0001; 95% prediction interval, 0.618 to 0.734). Meanwhile, an NRTI, lamivudine, improves insulin sensitivity and reduces inflammasome activation in diabetic and insulin resistance-induced human cells, as well as in mice fed with high-fat chow; mechanistically, inflammasome-activating short interspersed nuclear element (SINE) transcripts are elevated, whereas SINE-catabolizing DICER1 is reduced, in diabetic cells and mice. These data suggest the possibility of repurposing an approved class of drugs for prevention of diabetes.

## Abstract (web summary)

Inflammasome activation may contribute to type 2 diabetes, but whether targeting inflammasome is beneficial is unclear. Here the authors show that repurposing nucleoside reverse transcriptase inhibitors for inhibiting inflammasome activation is associated with reduced diabetes development in people and improves insulin sensitivity in experimental settings.

## Introduction

The number of people with diabetes worldwide is nearly 500 million and is projected to grow dramatically in the coming decades^1^. Most of these individuals have type 2 diabetes, a chronic metabolic disease characterized by insulin resistance and hyperglycemia^2^.

Chronic inflammation is a critical facet of type 2 diabetes^3,4^. The NLRP3 inflammasome, a multimeric protein complex, is implicated as a key driver of type 2 diabetes^5,6^. Activation of this inflammasome occurs in response to multiple danger-associated molecular patterns including several molecules involved in the pathogenesis of type 2 diabetes: glucose, islet amyloid polypeptide, free fatty acids, and mitochondrial reactive oxygen species^7,8^, and leads to production of the mature forms of the proinflammatory cytokines IL-1β and IL-18^9^. In animal models, inflammasome inhibition protects against insulin resistance^6,10–13^. Importantly, inflammasome activation is observed in circulating cells and adipose tissue of patients with insulin resistance^6,14,15^, and plasma concentrations of IL-1β and IL-18 are elevated in patients with type 2 diabetes and predict development of this disease^16,17^.

One of the activators of this inflammasome is RNA derived from *Alu* mobile genetic elements^18^. *Alu* RNAs recently are implicated in Alzheimer’s disease^19^, macular degeneration^18,20,21^, and systemic lupus erythematosus^22^. Elevated levels of *Alu* RNA and inflammasome activation in macular degeneration result from reduced levels of the enzyme DICER1, one of whose metabolic functions is to catabolize *Alu* RNAs^20,23,24^. Interestingly, DICER1 levels are decreased in circulating cells in patients with type 2 diabetes^25,26^, and deletion of *DICER1* in adipose or pancreatic islet beta cells triggers insulin resistance and diabetes in mice^27–30^. Given the expanding array of human disorders in which mobile genetic elements are recognized to play a pathogenic role^31^, and because DICER1 and inflammasome activation are implicated in diabetes, we explore whether *Alu* RNAs might be dysregulated in this disease as well.

*Alu* mobile elements propagate themselves by hijacking the endogenous reverse-transcriptase LINE-1^31,32^. Notably, nucleoside reverse-transcriptase inhibitors (NRTIs), drugs that are used to treat HIV-1 and hepatitis B infections, inhibit not only viral reverse-transcriptases but also LINE-1 reverse-transcriptase activity^33,34^. NRTIs also block inflammasome activation by *Alu* RNAs and other stimuli, independent of their ability to block reverse-transcriptase^35^. Therefore, we seek to determine whether, among patients with HIV-1 or hepatitis B, there is a relation between exposure to NRTIs and development of type 2 diabetes.

Here we employ a health insurance claims database analyses approach to examine the link between NRTI use and incident type 2 diabetes. We also investigate the effect of the NRTI lamivudine on insulin resistance in diabetic human adipocytes and myocytes and in a mouse model of type 2 diabetes to obtain experimental evidence in support of the clinical observations. We find that NRTI exposure is associated with reduced development of type 2 diabetes in people and that lamivudine inhibits inflammasome activation and improves insulin sensitivity in experimental systems. These data suggest the possibility of either repurposing this approved class of drugs or exploring less toxic modified NRTIs for treating prediabetes or diabetes.

## Results

### NRTIs associated with reduced hazard of developing diabetes

We examined associations between exposure to NRTIs (a list of specific medications is in Supplementary Table 1) and subsequent development of type 2 diabetes in the Veterans Health Administration, the largest integrated healthcare system in the United States, that was studied for over a 17-year period. To confirm these main findings, we then studied four other health insurance databases comprising diverse populations. In each of the five cohorts that included a total of 128,861 patients (Supplementary Fig. 1), we determined the association between NRTI use and the hazard of developing type 2 diabetes after adjustment for sociodemographic factors, overall health, comorbidities (a list of specific disease codes is in Supplementary Table 2), and use of medications that are known to alter risk of diabetes development.

**Fig. 1:**
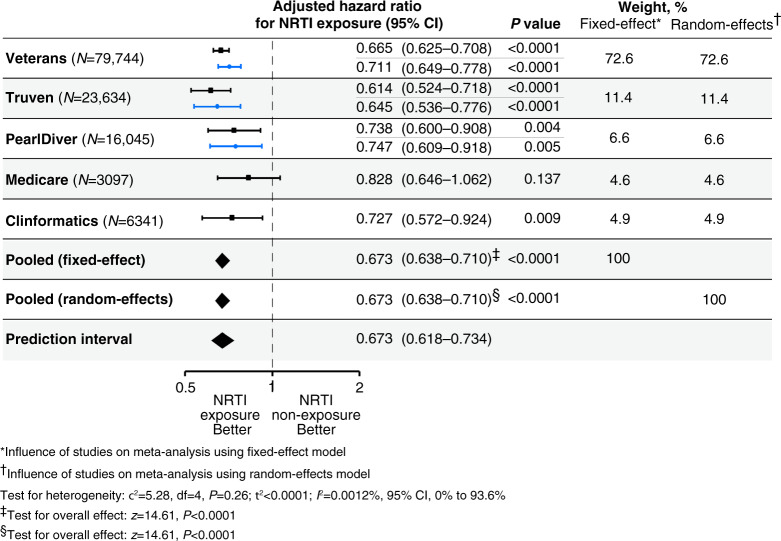
Forest plot of incident diabetes. Hazard ratios based on a Cox proportional-hazards model and adjusted for the confounding variables listed in Supplementary Tables 5, 7, 9, 11, and 13 were estimated separately for each database. The dashed vertical line denotes a hazard ratio of 1.0, which represents no difference in risk between nucleoside reverse-transcriptase inhibitor (NRTI) exposure and non-exposure. The black horizontal bars represent 95% confidence intervals (CI) for unmatched models. The blue horizontal bars represent 95% CI for propensity score-matched models. *P* values derived from *z* tests for individual databases are reported. Inverse-variance-weighted random-effects and fixed-effect meta-analyses were performed to obtain a pooled estimate of the adjusted hazard ratio of incident diabetes for NRTI exposure (ever versus never). The prediction interval is reported. The estimate of heterogeneity (τ^2^) and the results of the statistical test of heterogeneity using the chi-square (χ^2^) test statistic and its degrees of freedom (df) are shown below the plot. The Higgins *I*^2^ statistic and its 95% CI are presented. The results of the statistical tests of overall effect, the *z* test statistics, and corresponding *P* values are presented. All tests were two-tailed.

In our main analysis of the Veterans Health Administration database, which comprises predominantly men, 79,744 patients with confirmed diagnoses of HIV or hepatitis B and without a prior diagnosis of type 2 diabetes were identified (Supplementary Table 3). Of this group (baseline characteristics in Supplementary Table 4), 12,311 patients developed incident type 2 diabetes. After adjustment for potential confounders, users of NRTIs had 34% reduced hazard of developing type 2 diabetes (hazard ratio, 0.665; 95% CI, 0.625 to 0.708; *P* < 0.0001) (Fig. 1, Supplementary Table 5).

In the Truven database, which comprises employer-based health insurance claims, 23,634 patients with confirmed diagnoses of HIV or hepatitis B and without a prior diagnosis of type 2 diabetes were identified (Supplementary Table 3). Of this group (baseline characteristics in Supplementary Table 6), 1630 patients developed incident type 2 diabetes. After adjustment for potential confounders, users of NRTIs had 39% reduced hazard of developing type 2 diabetes (hazard ratio, 0.614; 95% CI, 0.524–0.718; *P* < 0.0001) (Fig. 1, Supplementary Table 7).

In the PearlDiver database, which comprises predominantly private health insurance claims, 16,045 patients with confirmed diagnoses of HIV or hepatitis B and without a prior diagnosis of type 2 diabetes were identified (Supplementary Table 3). Of this group (baseline characteristics in Supplementary Table 8), 1068 patients developed incident type 2 diabetes. After adjustment for potential confounders, users of NRTIs had 26% reduced hazard of developing type 2 diabetes (hazard ratio, 0.738; 95% CI, 0.600–0.908; *P* = 0.004) (Fig. 1, Supplementary Table 9).

In the Medicare 20% sample (primarily men and women 65 years or older), 3,097 patients with confirmed diagnoses of HIV or hepatitis B and without a prior diagnosis of type 2 diabetes were identified (Supplementary Table 3). Of this group (baseline characteristics in Supplementary Table 10), 707 patients developed incident type 2 diabetes. After adjustment for potential confounders, users of NRTIs had a 17% reduced hazard of developing type 2 diabetes (hazard ratio, 0.828; 95% CI 0.646–1.062; *P* = 0.137) (Fig. 1, Supplementary Table 11).

In the Clinformatics dataset, which comprises predominantly commercial health insurance claims, we identified 6341 patients with confirmed diagnoses of HIV or hepatitis B and without a prior diagnosis of type 2 diabetes (Supplementary Table 3). Of this group (baseline characteristics in Supplementary Table 12), 1067 patients developed incident type 2 diabetes. After adjustment for potential confounders, users of NRTIs had 27% reduced hazard of developing type 2 diabetes (hazard ratio, 0.727; 95% CI, 0.572–0.924; *P* = 0.009) (Fig. 1, Supplementary Table 13).

Given the low proportion of the observed variance among the five studies that could be attributed to heterogeneity (*I*^2^ = 0.0012%; 95% CI, 0.0–93.6%; *P* = 0.26, test of heterogeneity), summary risks were calculated using both fixed-effect and random-effects models, which yielded identical estimates and confidence intervals because of the extremely low estimate of heterogeneity (τ^2^ < 0.0001). Collectively, among 128,861 patients with HIV-1 or hepatitis B, users of NRTIs had 33% reduced hazard of developing type 2 diabetes (adjusted hazard ratio, 0.673; 95% CI, 0.638–0.710; *P* < 0.0001, test of overall effect; 95% prediction interval, 0.618–0.734) (Fig. 1).

### Bayesian meta-analysis

**Fig. 2:**
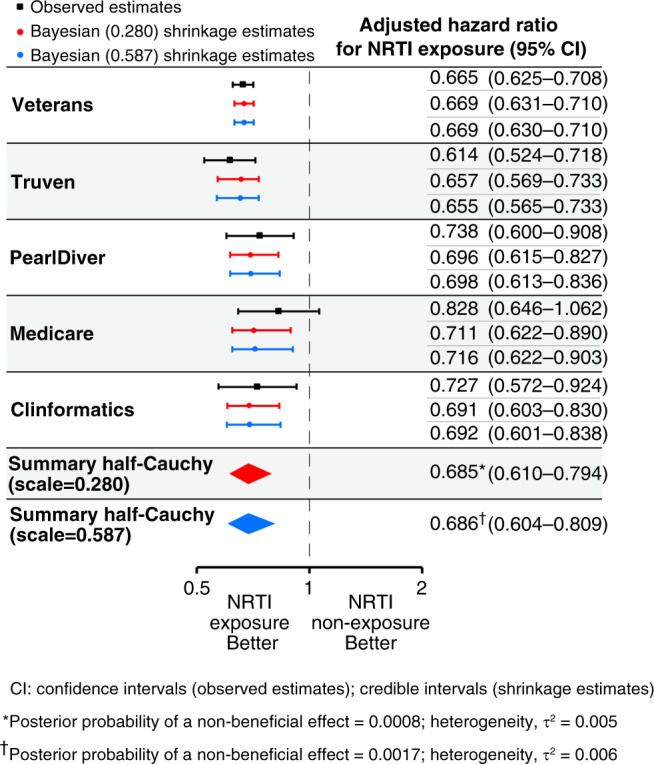
Bayesian meta-analysis of incident diabetes. Hazard ratios based on a Cox proportional-hazards model and adjusted for the confounding variables listed in Supplementary Tables 5, 7, 9, 11, and 13 were estimated separately for each database and are shown in black along with their 95% confidence intervals. A Bayesian meta-analysis was performed using a random-effects model and a weakly informative hierarchical half-Cauchy prior distribution^85,86^ for between-study variance with the assumption that it was unlikely for the between-study hazard ratios to vary by more than 3-fold (scale = 0.280). A sensitivity analysis to the choice of the prior by assuming that it was unlikely for the between-study hazard ratios to vary by more than 10-fold was also performed (scale = 0.587). The Bayesian shrinkage estimates and the summary estimates of the adjusted hazard ratio of incident diabetes for NRTI exposure (ever versus never), along with the 95% credible intervals, are shown in red (scale = 0.280) and blue (scale = 0.587). The dashed vertical line denotes a hazard ratio of 1.0, which represents no difference in risk between nucleoside reverse-transcriptase inhibitor (NRTI) exposure and non-exposure. The estimates of heterogeneity (τ^2^) and the posterior probabilities of a non-beneficial effect for each model are shown below the plot.

To complement this classical “frequentist” approach to meta-analysis, we performed a Bayesian meta-analysis using a random-effects normal-normal hierarchical model, which accounts for uncertainty in the estimation of the between-study variance. We used a weakly informative half-Cauchy prior distribution for between-study variability (τ) with the assumption that it was unlikely for the between-study hazard ratios to vary by more than 3-fold (scale = 0.280). In this model, collectively, among the patients with HIV-1 or hepatitis B in the five databases, users of NRTIs had a 32% median reduced hazard of developing type 2 diabetes (adjusted hazard ratio, 0.685; 95% credible interval, 0.610 to 0.794; *P*(HR > 1) = 0.0008, posterior probability of a non-beneficial effect) (Fig. 2). We performed a sensitivity analysis of these results to the choice of the prior by assuming that it was unlikely for the between-study hazard ratios to vary by more than 10-fold (scale = 0.587). The posterior distribution was quite robust to changes in the scale as the summary effect was remarkably insensitive to the choice of the prior: applying this model, users of NRTIs had a 31% median reduced hazard of developing type 2 diabetes (adjusted hazard ratio, 0.686; 95% credible interval, 0.604 to 0.809; *P*(HR > 1) = 0.0017, posterior probability of a non-beneficial effect) (Fig. 2). In both models, the estimate of heterogeneity (τ^2^) was low (0.005–0.006). These Bayesian meta-analyses yielded estimates that were qualitatively similar and directionally identical to the frequentist meta-analyses.

### Sensitivity analyses

Next we performed three types of sensitivity analyses in the main Veterans cohort. First, a single hazard ratio averaged over the entire study duration may not necessarily reflect a robust measure of the exposure effect because the hazard ratio may change over time^36^. Therefore, we computed the average hazard ratios after 1, 2, 5, and 10 years of follow-up. After adjustment for potential confounders, users of NRTIs had a reduced hazard of developing type 2 diabetes over each of these time periods (Supplementary Table 14). In addition, we plotted survival curves adjusted for baseline confounders (Supplementary Fig. 2). None of the period-specific hazard ratios crossed 1.0 nor did the adjusted survival curves for the NRTI-exposed and NRTI-unexposed groups cross one another, suggesting that the protective association of NRTI use against the development of type 2 diabetes was maintained throughout the study period.

Second, in chronic diseases such as type 2 diabetes, the competing risk of death can preclude the diagnosis of diabetes. Therefore, we performed a competing risk regression analysis, which was possible in the Veterans cohort as it contains comprehensive mortality data^37^, but not in the other four databases we studied as they do not provide mortality information. The follow-up duration and mortality rates were comparable between NRTI users and NRTI non-users in the Veterans cohort (Supplementary Table 15). Among these 79,744 patients (who account for the majority of the patients in all 5 databases), using a competing risk of mortality analysis and using the same list of covariates as the primary analysis, NRTI use was associated with a 27% reduced risk of incident type 2 diabetes (adjusted subdistribution hazard ratio, 0.727; 95% CI, 0.683–0.775; *P* < 0.0001) (Supplementary Table 16). These data, which are similar to risk reduction observed in the primary analysis, suggest that the differential mortality rates are not responsible for the observed risk reduction of incident type 2 diabetes among NRTI users in the Veterans cohort.

Third, as the NRTI exposure prevalence was markedly different between HIV-positive and hepatitis B-positive persons (Supplementary Tables 17–21), we performed another sensitivity analysis by analyzing these populations separately. The proportions of HIV-positive patients who did not have a record of NRTI exposure (VA – 34%; Truven – 1%; PearlDiver – 17%; Medicare – 19%; Clinformatics – 24%) were similar to previously reported rates^38,39^. Because this subgroup analysis markedly reduces the sample sizes, we again studied the largest Veterans cohort. Among HIV-positive hepatitis B-negative individuals, NRTI use was associated with a 38% reduced risk of incident type 2 diabetes (adjusted hazard ratio, 0.621; 95% CI, 0.562–0.685; *P* < 0.0001). Among hepatitis B-positive HIV-negative individuals, NRTI use was associated with a 28% reduced risk of incident type 2 diabetes (adjusted hazard ratio, 0.717; 95% CI, 0.656–0.783; *P* < 0.0001). Collectively, these analyses (Supplementary Tables 22 and 23) suggest that NRTI exposure is beneficial in reducing incident type 2 diabetes risk among HIV-positive as well as hepatitis B-positive individuals in the Veterans cohort.

### Continuous exposure modeling

Next, we studied NRTI exposure as a continuous rather than categorical covariate by estimating the hazard of developing type 2 diabetes as a function of per year of NRTI exposure. This approach addresses, in part, the issue of allocation bias. By focusing inferences on how the outcome of incident type 2 diabetes depends on cumulative exposure to NRTIs, this approach also provides insight into potential dose-response effects. In each of the five databases, there was a reduced hazard of developing type 2 diabetes with each increasing year of NRTI exposure (Supplementary Tables 24–28). Summary risks were calculated by performing meta-analyses using both fixed-effect and random-effects models. Collectively, among the patients with HIV-1 or hepatitis B in the five databases, users of NRTIs had 3–8% reduced hazard of developing type 2 diabetes with each additional year of use (fixed-effect: adjusted hazard ratio per year of NRTI exposure, 0.974; 95% CI, 0.967 to 0.980; *P* < 0.0001, test of overall effect; random-effects: adjusted hazard ratio, 0.922; 95% CI, 0.872–0.976; *P* = 0.005, test of overall effect) (Supplementary Fig. 3).

In contrast, we did not observe any consistent association across the five databases between incident development of type 2 diabetes and exposure to three other drug classes used to treat persons with HIV-1 infection: non-nucleoside reverse transcriptase inhibitors, protease inhibitors, or integrase inhibitors (Supplementary Tables 24–28).

### Falsification testing

To test for residual confounding, we conducted falsification tests using the two outcomes of appendicitis and hernia, which were not anticipated to be associated with NRTI exposure, among patients with confirmed diagnoses of HIV or hepatitis B and without a prior diagnosis of these outcomes. NRTI use was not associated with reduced hazard of developing incident appendicitis (Supplementary Fig. 4) or hernia (Supplementary Fig. 5) in any of the five databases individually or in pooled fixed-effect or random-effects model meta-analyses.

### Propensity score matching analysis

As assignment to NRTI treatment was not randomized, differences in incident diabetes might result from different characteristics of the treatment groups rather than NRTI usage itself. Therefore, we used propensity-score matching to assemble cohorts of patients with similar baseline characteristics and thereby reduced possible bias in estimating treatment effects. Because this procedure markedly reduces the original patient sample size, we confined these analyses to the three largest databases: Veterans Health Administration, Truven Marketscan, and PearlDiver databases. In the Veterans database, 9057 patients who had NRTI exposure were matched with 9057 patients who did not have NRTI exposure. In the Truven database, 4343 patients who had NRTI exposure were matched with 4343 patients who did not have NRTI exposure. In the PearlDiver database, 2153 patients who had NRTI exposure were matched with 2153 patients who did not have NRTI exposure. To further control for any residual covariate imbalance, we adjusted for all of the sociodemographic factors, overall health, comorbidities, and use of medications known to alter risk of diabetes development that were employed for the original unmatched group analyses. In all three databases, after adjustment for potential confounders, users of NRTIs had a reduced hazard of developing type 2 diabetes (Veterans: hazard ratio, 0.711; 95% CI, 0.649–0.778; *P* < 0.0001; Truven: hazard ratio, 0.645; 95% CI, 0.536–0.776; *P* < 0.0001; PearlDiver: hazard ratio, 0.747; 95% CI, 0.609–0.918; *P* = 0.005) (Fig. 1). We also estimated hazard ratios as a function of per year of NRTI exposure in the propensity-score matched groups. In all three databases, after adjustment for potential confounders, users of NRTIs had a reduced hazard of developing type 2 diabetes with each additional year of use (Veterans: hazard ratio, 0.979; 95% CI, 0.959–0.999; *P* = 0.042; Truven: hazard ratio, 0.926; 95% CI, 0.857–0.999; *P* = 0.049; PearlDiver: hazard ratio, 0.830; 95% CI, 0.719–0.958; *P* = 0.01) (Supplementary Tables 29–31 and Supplementary Figs. 6–11). The small differences in the hazard estimates between the unmatched and propensity-score-matched analyses suggests that the residual bias in the unmatched analyses is likely to be small.

### NRTI reduces insulin resistance and inflammasome activation

Next we investigated one potential activator of the NLRP3 inflammasome: RNA derived from *Alu* mobile genetic elements^18^, which have been implicated in other human diseases such as Alzheimer’s disease^19^, macular degeneration^18,20,21^, and systemic lupus erythematosus^22^. In macular degeneration, elevated levels of *Alu* RNA and inflammasome activation result from reduced levels of the enzyme DICER1, one of whose metabolic functions is to catabolize *Alu* RNAs^20,23,24^. DICER1 levels are decreased in circulating cells in patients with type 2 diabetes^25,26^, and deletion of *DICER1* in adipose or pancreatic islet beta cells triggers insulin resistance and diabetes in mice^27–30^. Given that many human disorders are associated with pathogenic mobile genetic elements^31^, and because DICER1 and inflammasome activation are implicated in diabetes, we explored whether *Alu* RNAs might be dysregulated in this disease as well.

**Fig. 3:**
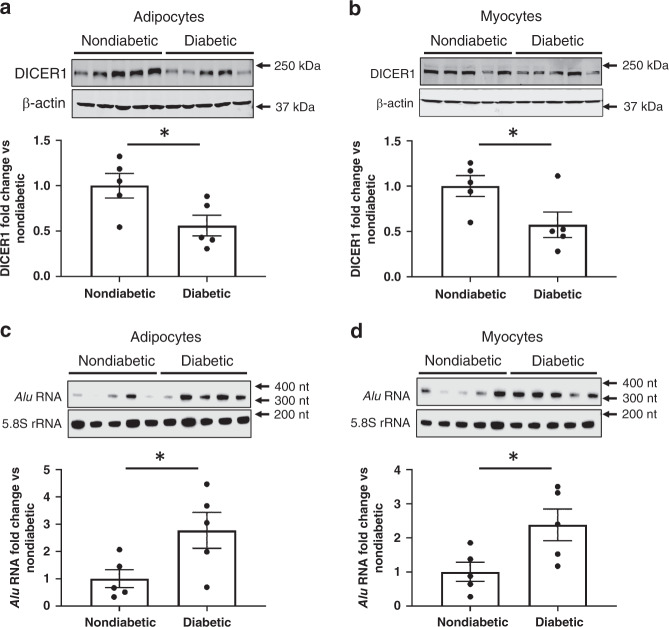
Expression of DICER1 and *Alu* in human diabetic adipocytes and skeletal myocytes. The top panels show the results of western blotting of extracts of proteins from human adipocytes **a** and human myocytes **b** isolated from nondiabetic and diabetic persons. Immunoreactive bands corresponding to DICER1 and beta-actin (β-actin) are shown. The bottom panels show bar graphs of the densitometric analyses of the DICER1 western blots in the top panels that have been normalized to β-actin abundance and to the nondiabetic group data. *P = 0.04 (diabetic versus nondiabetic adipocytes), *P = 0.046 (diabetic versus nondiabetic myocytes), two-tailed unpaired Student *t* test. The top panels show the results of northern blotting of total RNA extracts from human adipocytes **c** and human myocytes **d** isolated from nondiabetic and diabetic persons. Hybridization bands corresponding to *Alu* RNA and 5.8S ribosomal RNA (5.8S rRNA) are shown. The bottom panels show bar graphs of the densitometric analyses of the *Alu* northern blots in the top panels that have been normalized to 5.8S rRNA abundance and to the nondiabetic group data. *P = 0.04 (diabetic versus nondiabetic adipocytes), *P = 0.03 (diabetic versus nondiabetic myocytes), two-tailed unpaired Student *t* test. Data are reported as mean ± s.e.m. *n* = 5 samples per group **a**–**d**. Source data are provided as a Source Data file.

**Fig. 4:**
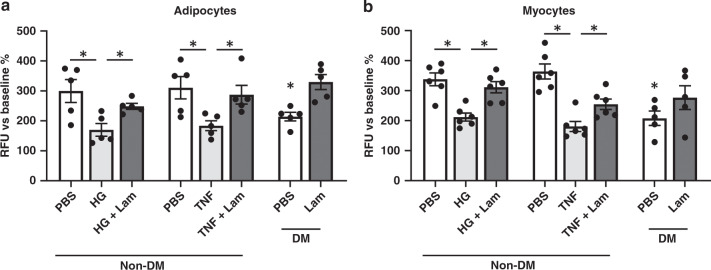
Insulin-induced glucose uptake in human adipocytes and skeletal myocytes. The results of glucose uptake assays in human adipocytes **a** and in human skeletal myocytes **b** are shown. Cells from nondiabetic (Non-DM) persons were treated with either tumor necrosis factor (TNF; 2.5 nM) or with high glucose (25 mM) and high insulin (100 nM) (HG) to induce insulin resistance. Cells from diabetic (DM) and nondiabetic persons were treated with lamivudine (Lam; 100 μM) or phosphate-buffered saline (PBS; vehicle). Glucose uptake measurements were performed by exposing cells to insulin (20 nM) using a fluorescent derivative of glucose, and quantified as relative fluorescence units (RFU) and normalized to baseline (prior to insulin treatment) levels of fluorescence. Data are reported as mean ± s.e.m.* In Non-DM adipocytes, *P* = 0.02 (HG versus PBS), *P* = 0.01 (HG + 3TC versus HG), *P* = 0.01 (TNF versus PBS), *P* = 0.02 (TNF + 3TC versus TNF), two-tailed unpaired Student *t* test. In DM adipocytes, *P* = 0.02 (3TC versus PBS), two-tailed paired Student *t* test. *n* = 5 samples per group (**a**). In Non-DM myocytes, *P* < 0.001 (HG versus PBS), *P* = 0.001 (HG + 3TC versus HG), *P* < 0.001 (TNF versus PBS), *P* = 0.01 (TNF + 3TC versus TNF), two-tailed unpaired Student *t* test. In DM myocytes, *P* = 0.03 (3TC versus *P*BS), two-tailed paired Student *t* test. *n* = 6 (nondiabetic) or 5 (diabetic) samples per group (**b**). Source data are provided as a Source Data file.

Primary cells isolated from the adipose or skeletal muscle tissues of type 2 diabetes patients expressed lower levels of DICER1 protein (Fig. 3a, b) and higher levels of *Alu* RNA (Fig. 3c, d) compared with the cells of nondiabetic individuals. Insulin-induced glucose uptake into cells was impaired in diabetic adipocytes and myocytes; this resistance to insulin was reversed by lamivudine treatment (Fig. 4a, b). Insulin resistance was induced in nondiabetic adipocytes and myocytes by either TNF exposure or treatment with high glucose and high insulin; lamivudine prevented insulin resistance induced in both these models (Fig. 4a, b). Phosphorylation of the protein kinase AKT in response to insulin stimulation, a key signaling event in insulin-dependent glucose uptake^40^, was impaired in diabetic adipocytes and myocytes; this resistance to insulin was reversed by lamivudine treatment (Supplementary Fig. 12). Lamivudine also restored AKT phosphorylation in nondiabetic adipocytes and myocytes rendered insulin resistant by TNF treatment (Supplementary Fig. 12). These data suggest that lamivudine might ameliorate insulin resistance in part via AKT-dependent pathways.

Since NRTIs as a class exhibit inflammasome-inhibitory effects^35^, we explored whether other members of this drug class also exerted similar effects. We found that, similar to lamivudine, both azidothymidine and stavudine exerted beneficial effects on insulin-induced AKT phosphorylation in diabetic adipocytes and in nondiabetic adipocytes rendered insulin resistant by TNF treatment (Supplementary Fig. 13). At the doses tested, all three NRTI drugs had no deleterious effect on cell viability (Supplementary Fig. 14). Collectively, these data suggest that several NRTI drugs exhibit class-effects on ameliorating insulin resistance.

**Fig. 5:**
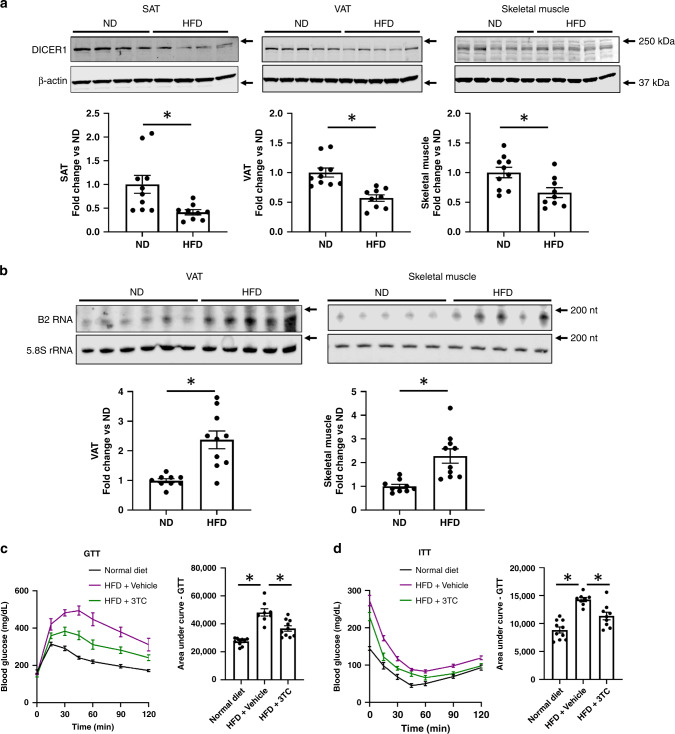
Expression of DICER1, B2, and insulin sensitivity in high-fat diet-fed mice. **a** The top three panels show the results of western blotting of extracts of proteins from subcutaneous adipose tissue (SAT), visceral adipose tissue (VAT), and skeletal muscle tissue isolated from mice fed a normal diet (ND) or a high-fat diet (HFD). Immunoreactive bands corresponding to DICER1 and beta-actin (β-actin) are shown. The bottom three panels show bar graphs of the densitometric analyses of the DICER1 western blots in the top panels that have been normalized to β-actin abundance and to the ND group data. *n* = 10 (ND) or 9 (HFD) samples per group. *P = 0.01 (ND versus HFD in SAT), *P* < 0.001 (ND versus HFD in VAT), *P* = 0.01 (ND versus HFD in Skeletal muscle), two-tailed unpaired Student *t* test. **b** The top two panels show the results of northern blotting of total RNA extracts from visceral adipose tissue (VAT) and skeletal muscle tissue isolated from mice fed a normal diet (ND) or a high-fat diet (HFD). Hybridization bands corresponding to B2 RNA and 5.8S ribosomal RNA (5.8S rRNA) are shown. The bottom two panels show bar graphs of the densitometric analyses of the B2 northern blots in the top panels that have been normalized to 5.8S rRNA abundance and to the ND group data. *n* = 9 (ND) or 10 (HFD) samples per group. *P < 0.001 (ND versus HFD in VAT) and *P* = 0.001 (ND versus HFD in Skeletal muscle), two-tailed unpaired Student *t* test. Glucose tolerance test (GTT; **c**) and insulin tolerance test (ITT; **d**) measurements, and area under the curve (AUC) quantification in mice fed a normal diet, a high-fat diet and treated with phosphate-buffered saline vehicle (HFD + Vehicle), or a high-fat diet and treated with once-daily intraperitoneal administration of lamivudine (70 mg/kg of body weight) (HFD + Lam). Data are reported as mean ± s.e.m. *n* = 10 (ND), 8 (HFD + Vehicle), or 10 (HFD + Lam) samples per group **c**. *n* = 10 (ND), 8 (HFD + Vehicle), or 9 (HFD + Lam) samples per group **d**. *In GTT, *P* < 0.001 (HFD + Vehicle versus Normal Diet) and *P* = 0.002 (HFD + 3TC versus HFD + Vehicle), two-tailed unpaired Student *t* test. *In ITT, *P* < 0.001 (HFD + Vehicle versus Normal Diet) and *P* = 0.003 (HFD + 3TC versus HFD + Vehicle), two-tailed unpaired Student t test. Source data are provided as a Source Data file.

High-fat diet-fed mice are used to model impaired glucose tolerance and type 2 diabetes^41^. In mice raised on a high-fat diet for 8 weeks, we measured higher RNA levels of B2, a rodent *Alu*-like mobile genetic element, and lower protein levels of DICER1 in their adipose and muscle tissues compared to regular diet-fed mice (Fig. 5a, b). Glucose tolerance and insulin sensitivity in high-fat diet-fed mice, as monitored by glucose tolerance tests and insulin tolerance tests, respectively, were improved by once-daily intraperitoneal administration of lamivudine (Fig. 5c, d). Insulin stimulation of AKT phosphorylation was impaired in the subcutaneous and visceral adipose tissues and skeletal muscle of high-fat diet-fed mice; lamivudine-treated mice retained the activity of this insulin signaling pathway (Supplementary Fig. 15). Protein levels of IL-1β or IL-18, which are products of inflammasome activation, were elevated in the subcutaneous and visceral adipose tissue and skeletal muscle of high-fat diet-fed mice; lamivudine treatment inhibited the increase in these cytokine levels (Supplementary Fig. 16). Of note, lamivudine did not alter high-fat diet-induced gain in body-weight (Supplementary Fig. 17), indicating that its salutary effects were not due to weight reduction. Collectively, these data suggest that lamivudine increased sensitivity to endogenous insulin and reduced inflammasome activation in the context of a high-fat diet.

## Discussion

We identify an association between exposure to NRTIs and lower rates of development of type 2 diabetes among persons with HIV-1 or hepatitis B infection. We also present biochemical evidence that the NRTI lamivudine restores insulin sensitivity in type 2 diabetic human cells and prevents induction of insulin resistance in non-diabetic human cells. At doses allometrically scaled to those used in humans, lamivudine improves glucose tolerance and insulin sensitivity and reduces inflammasome activation in high-fat diet-fed mice. These investigations of human cell, mouse, and population database systems collectively suggest a potential beneficial effect of NRTIs in forestalling diabetes onset.

In the main analysis of NRTI exposure versus incident type 2 diabetes risk, the pooled summary estimate of the adjusted hazard ratio across the five databases (0.673) and confidence interval (0.638–0.710) provide information on how well we have determined the mean effect. In contrast, the prediction interval (0.618–0.734) illuminates the range of true effects that can be expected in future settings by providing an estimate of an interval in which a future observation, e.g., the result of a future clinical trial, will fall^42^. From this prediction interval, we infer that the probability that a future study, e.g., a clinical trial, observes a beneficial effect of NRTIs (i.e., hazard ratio <1.0) is 99.99% (calculations in Methods). Similarly, we calculate that there is a 95% probability that such a future study will observe a hazard ratio of less than 0.713, i.e., a reduced risk of at least 29%. Likewise, we estimate a 50% probability that a future study would observe a hazard ratio of less than of 0.673 (a reduced risk of at least 33%). However, such inferences are only valid in settings that are exchangeable, i.e., similar, to those on which our meta-analysis is based.

Repurposing of existing drugs is an urgent priority for revitalizing, accelerating, and optimizing drug development^43^. Not all NRTIs are suitable candidates for repurposing. First-generation NRTIs such as stavudine and didanosine are more toxic than subsequently deployed NRTIs (lamivudine, emtricitabine, tenofovir), induce mitochondrial toxicity and lipodystrophy, and are associated with induction of insulin resistance and increased risk of type 2 diabetes in HIV-infected individuals, particularly when combined with PIs^44–47^. In the five insurance databases we analyze, over the time-periods studied (2000–2017), stavudine and didanosine use ranges from 3–9%, thus limiting the likelihood that their use significantly influences rates of diabetes development in these populations. The association of NRTI use with reduced incident diabetes in the cohorts we study might also reflect the much larger populations we study as well as our inclusion of multiple comorbidities and concomitant medication use that are known to affect the development of diabetes.

Limitations to our observational study include limitations intrinsic to all health insurance claims database analyses, particularly proper documentation and coding^48,49^. However, collectively these five databases encompass 150 million patient lives spanning a multidimensionally diverse array of populations in terms of age, gender, geography, race, and time-period. A notable strength of our study is that our clinical findings are replicated in five independent and geographically dispersed cohorts that collectively represent the majority of adults with health insurance in the United States. In addition, the Bayesian meta-analysis approach, which has advantages in terms of accounting for parameter uncertainty^50^ and the generation of credible intervals that account for the prior distribution^51^, parallel the results of the frequentist meta-analysis results, thereby increasing confidence in the main finding that NRTI exposure is associated with a risk in incident type 2 diabetes.

In addition to the main analysis that analyzes exposure to NRTIs as a binary covariate (ever versus never), our analysis of NRTI exposure as a continuous cumulative exposure covariate (per year of exposure), is also associated with reduced incident type 2 diabetes. By focusing inferences on how the outcome of incident type 2 diabetes depends on cumulative exposure to NRTIs, this approach deals, in part, with fixed between-person confounding that may result from unmeasured confounders that result in ever-users and never-users of NRTIs having differential susceptibility to the outcome of diabetes for reasons independent of NRTI use. However, analyses of cumulative NRTI exposure may be affected by time-varying risk factor confounding; thus, estimates provided by the Cox models for the time-updated variable of years of NRTI exposure could be biased.

We study an extensive number of demographic variables, comorbid conditions, concomitant medication use, and laboratory tests that are known risk factors for development of diabetes by including them as fixed risk factor covariates whose values are considered at baseline (index date). Several of these risk factors could have changed with time; however, we do not consider their time-dependent variance for several reasons. First, the availability of information on many of the variables, e.g., body mass index or CD4+ counts, is not uniform over the entire follow-up duration. Second, it is not altogether clear how the time-dependent variance of several of these variables, e.g., body mass index or concomitant medication use, impacts ongoing risk of incident type diabetes, because of complex, non-linear effects and unknowable biological interactions. Third, certain risk factors have greater short-term effects whereas others have greater long-term effects on chronic disease outcomes^52^, and often these are ill-defined. Use of time-dependent covariates also runs the risk that the value of a covariate during follow-up could change as a result of risk factors being studied^52^. Nevertheless, we acknowledge that our use of risk factors as covariates fixed at baseline could understate or overstate their influence on the association of NRTI use with development of diabetes.

Another limitation of insurance claims analyses is the non-availability of information on diet, physical activity, and stress, all of which influence development of diabetes. We do, however, control for numerous comorbidities, medications, and laboratory abnormalities known to influence rates of development of diabetes. Despite covariate adjustment for a large number of relevant confounders and performing robust propensity score matching, we cannot rule out the possibility of selection bias or residual confounding. However, the likelihood that unmeasured confounding accounts for identification of an association of NRTI exposure with incident diagnoses of diabetes is diminished by the observation that exposures to NNRTIs, PIs, or INSTIs, which serve as negative controls for medication usage^53^, are not associated with reduced incident type 2 diabetes. In addition, the results of propensity score matching and falsification testing (which detects confounding, selection bias and measurement error)^53–55^ both increase the internal validity of the conclusions of our main analyses. We also note that the impact of loss of follow-up due to mortality is assessed only in the Veterans cohort and not in the other databases. As our analyses are restricted to patients with HIV-1 or hepatitis B, our results might not be applicable to other populations. However, the protective effects of lamivudine are evident in human cells and mice not infected with these viruses. Therefore, the protective effects of NRTIs, which, as a class, block inflammasome activation in other models of non-infectious disease^35,56^, might extend beyond the setting of viral infections.

All statistical modeling approaches, including the many we employ, are subject to inherent assumptions and limitations. Analyzing nonlinear covariate effects as well as more complex interactions could potentially provide better model fits in the individual datasets. Propensity score-based methods such as propensity score matching, which we employ, are widely used to draw causal inferences from observational studies^57–60^. Alternative causal reasoning frameworks^61,62^ and introducing synthetic positive controls^63^ offer additional approaches to analyzing observational data, and could be worthwhile exploring in future studies. Ultimately, prospective randomized trials can provide the best insights into causality.

In addition to providing clinical evidence supportive of the inflammasome hypothesis of type 2 diabetes, we introduce the concept that perturbation in the homeostasis of the DICER1-*Alu* RNA regulatory axis could be involved in triggering this aging-associated disease, as dysregulation of the DICER1-*Alu*/B2 RNA pathway is evident in adipose and muscle cells of type 2 diabetic humans and of high-fat diet-fed mice. Our findings expand the spectrum of pathologies potentially triggered by *Alu*, the most successful of human genomic parasites. Additional mechanistic and phenotypic studies of NRTI treatment in various animal models of type 2 diabetes would enhance confidence in a therapeutic effect.

More recently developed NRTIs are well tolerated and are associated with lower adverse event rates^64^. Randomized trials of lamivudine monotherapy in adults and children with hepatitis B^65–67^ and of other current-generation NRTIs in non-HIV-infected individuals^68,69^ have safety profiles that are similar to placebo treatment. However, lamivudine, although less toxic than its predecessors, is associated with development of rare adverse events such as lactic acidosis, hepatomegaly and steatosis, particularly when used in combination with other more toxic anti-retroviral drugs administered to sicker HIV patients in earlier eras, and in children^70^. Regulatory agency labels for NRTIs contain warnings of lactic acidosis, although it should be noted that currently-approved anti-diabetic medications such as metformin also carry these warnings. Nevertheless, it is prudent to explore in prospective trials whether modified NRTIs known as Kamuvudines, which retain the ability to inhibit inflammasome activation but lack attendant toxicities^35^, represent better candidates for treating prediabetes or diabetes.

Finally, we stress that despite our “triangulation”^71^ by integrating interlocking evidence from multiple approaches^72^ such as health insurance claims analyses performed on different cohorts by different investigators, cell culture studies, and animal models, we caution against advocating use of NRTIs in prediabetes or diabetes in the absence of prospective randomized clinical trials. Cost-benefit analyses of the future utilization of NRTIs following prospective evaluation should include consideration of their potential for inducing viral resistance as well as how they compare to certain diets and exercise regimens, which can benefit individuals with prediabetes^73–75^.

## Methods

### Data sources

Data were evaluated from five health insurance claims databases: U.S. Veterans Health Administration database (which includes health care claims information extracted from the VA Informatics and Computing Infrastructure) for the years 2000–2017; Truven Marketscan, which includes employer-based health insurance claims for the years 2006–2017; PearlDiver, which includes health care claims for persons in the Humana managed care network for the years 2007–2017; a random 20% sample of Medicare beneficiaries with Parts A, B, and D coverage for the years 2008–2016; and Clinformatics DataMart database (OptumInsight), which captures health care claims for persons in a large nationwide managed care network for the years 2001–2016. Disease-specific diagnoses using codes from the International Classification of Diseases, 9th Revision, Clinical Modification (ICD-9-CM) were evaluated. For the VA and Truven databases, codes from the 10th Revision, Clinical Modification (ICD-10-CM) (Supplementary Methods) were also evaluated using a cross-walk between ICD-10-CM and ICD-9-CM codes.

### Study population

Patients were included in these analyses if they had at least two medical claims for HIV/AIDS or hepatitis B during study dates, and were excluded if they had pre-existing diabetes, defined as at least one such ICD-9-CM/ICD-10-CM diagnosis prior to their ICD-9-CM /ICD-10-CM diagnosis of HIV/AIDS or hepatitis B. Baseline participant characteristics, described by means and standard deviations for continuous variables and frequencies and percentages for categorical variables, are presented in Supplementary Table 3.

### Exposure definition

Individuals were classified as receiving NRTI, NNRTI (nonnucleoside reverse-transcriptase inhibitor), PI (protease inhibitor), or INSTI (integrase strand transfer inhibitor) medications if at least one outpatient pharmacy prescription for these medications was filled. American Hospital Formulary Service drug codes and U.S. National Drug Codes (a list of specific medications is in Supplementary Table 1) were evaluated. Patients filling prescriptions for combination anti-viral medications were counted as having received medications from each class. Medication use was summarized as a time-dependent covariate measuring the cumulative days or months supplied.

### Outcomes

The main outcome was incident type 2 diabetes. Time to initial diagnosis of type 2 diabetes during the follow-up period was the dependent variable. Observations were right-censored at the end of plan enrollment, death, or diabetes development. Falsification tests were performed using the two prespecified outcomes of appendicitis and hernia, which were not anticipated to be associated with NRTI exposure, among patients with at least two medical claims for HIV or hepatitis B and without a prior diagnosis of these falsification outcomes. Time to initial diagnosis of appendicitis or hernia during the follow-up period were dependent variables for these analyses. Observations were right censored at the end of plan enrollment, death, or development of appendicitis or hernia.

### Statistical analyses for data sources

Key predictors were use of the HIV-1 and hepatitis B drugs NRTI, NNRTI, PI, and INSTI. Cox regression was used to estimate the hazard for developing type 2 diabetes in relation to NRTI, NNRTI, PI, and INSTI exposure, with adjustment for baseline covariates, which included demographic variables, comorbidities, use of other medications, and laboratory test values known to be associated with diabetes including those listed by the National Institute of Diabetes and Digestive and Kidney Diseases (NIDDK)^76^, the Centers for Disease Control and Prevention (CDC)^77^, and the International Diabetes Federation (IDF)^1^, and those identified by supplementary literature research; 95% confidence intervals for hazard ratios were constructed based on standard errors derived from the model. Schoenfeld’s global goodness-of-fit test^78,79^ were used to test the proportional hazards assumption of the Cox regression. In both the NRTI ever/never and the NRTI per year of exposure analyses in all 5 databases, all these *P* values were >0.05, confirming the validity of the proportional hazards assumption of the fitted models.

We used SAS software, version 9.4 (SAS Institute) and Excel, version 16.35 (Microsoft) to perform statistical analyses. An inverse- variance weighted analysis of the five databases combined was performed to estimate the combined hazard ratio and to compute 95% confidence intervals using fixed-effect and random-effects models. Meta-analyses were performed with the use of the statistical program R, version 3.6.1 (the R project [http://r-project.org]) and the R packages metafor and bayesmeta. The restricted maximum-likelihood estimator was used to estimate the between-study variance. A forest plot was created to depict the HR and 95% confidence intervals or credible intervals of each study and of the summary results. Statistical tests were two-sided. *P* values of less than 0.05 were considered to indicate statistical significance.

Frequentist meta-analysis (primary analysis): Variability among the five databases was evaluated using Cochran’s Q-test^80^. A random-effects model was used in the primary analyses as it assumes that individual databases are samples of different populations with different underlying true effects. In contrast, fixed-effect models assume that individual databases are samples from the same population^81–83^.

Bayesian meta-analysis (secondary analysis): Bayesian meta-analysis was performed using a random-effects normal-normal hierarchical model (the same as the random-effects model above). For the effect parameter μ, we choose a neutral unit information prior given by a normal prior with mean μ_p_ = 0 (centered around a hazard ratio of 1.0) and a variance of (σ_p_^2^ = 4)^84^. In a hierarchical model θ ~ N[μ, τ^2^], where τ^2^ is the between-study variance of the logarithmic hazard ratio, the “range” of hazard ratios, defined as the ratio of the 97.5% and 2.5% quantiles of the hazard ratio, is equal to (*e*^3.92τ^)^84^. We used a weakly informative half-Cauchy prior distribution^85,86^ for between-study variability with the assumption that it was unlikely for the between-study hazard ratios to vary by more than 3-fold. For this assumption, range = 3 and τ = 0.280 (scale). We performed a sensitivity analysis to the choice of the prior by assuming that it was unlikely for the between-study hazard ratios to vary by more than 10-fold. For this assumption, range = 10 and τ = 0.587 (scale). Computation of posterior predictive *P* values were implemented in bayesmeta via Monte Carlo sampling.

Falsification tests were performed using the two prespecified outcomes of appendicitis and hernia, which were not anticipated to be associated with NRTI exposure, among patients with at least two medical claims for HIV or hepatitis B and without a prior medical claim for these falsification outcomes. Time to initial diagnosis of appendicitis or hernia during the follow-up period were dependent variables for these analyses. Observations were right censored at the end of plan enrollment, death, or development of appendicitis or hernia. Additional information about the statistical analyses is provided in the Supplementary Methods.

### Propensity score matching

For the Veterans Health Administration, Truven Marketscan, and PearlDiver databases, we estimated propensity-score models including use of NRTIs and no use of NRTIs. The individual propensities for starting NRTI treatment were estimated with the use of logistic regression. As predictors, the propensity-score models included the set of variables which displayed *P* values < 0.1 in logistic regression analyses. In the Veterans Health Administration Database, these variables were thus used in the propensity score model: Labs (CD4 counts, viral load, body mass index), other medications (PI, NNRTI, lipid lowering agents, antihypertensives), comorbidities (Charlson comorbidity index, systemic hypertension, depression, ischemic heart disease, other heart disease, stroke, hepatitis C, osteoarthritis, rheumatoid arthritis), and demographics (age, race, index year, tobacco use). In the Truven Marketscan Database, these variables were thus used in the propensity score model: comorbidities (Charlson comorbidity index, osteoarthritis, rheumatoid arthritis, systemic hypertension, hyperglyceridemia, stroke, acanthosis nigricans, hepatitis C), demographics (age, sex, index year). In the PearlDiver Database, these variables were thus used in the propensity score model: Labs (HDL, triglycerides, CD4 counts, body mass index, ALT, AST), other medications (fluoroquinolones, corticosteroids, lipid lowering agents, antihypertensives), comorbidities (Charlson comorbidity index, osteoarthritis, systemic hypertension, pure hypercholesterolemia, hyperglyceridemia, ischemic heart disease, other heart disease, gestational diabetes), demographics (age, race, sex, index year, tobacco use, family history of diabetes). Matching was performed in a 1:1 ratio using greedy nearest neighbor matching. In addition, to control for any residual covariate imbalance, we estimated the relative hazard in the propensity score-matched groups using the multivariable Cox model that included the covariates from the multivariable regression analysis employed for the original unmatched group analyses. Statistical tests were two-sided. *P* values < 0.05 were considered statistically significant.

### Prediction interval and threshold probabilities

The prediction interval^42^ was computed using the metafor package in *R*. The probability *P* that the true effect in a new study will be below a desired threshold *D* was calculated with the left-tail cumulative t-distribution with k–1 degrees of freedom (df) for k studies in the meta-analysis. The probability that the effect is below *D* equals *P*. For HRs, calculations were based on the *ln* HR, with the summary meta-analysis estimate *μ* = 0.673, SD_PI_ = $\sqrt {\tau ^2 + SE^2} = 0.0272772$ (where τ^2^ is the estimated heterogeneity and SE is the standard error of μ), and df = 4. For example, to determine the probability of a null or protective effect, we computed the probability that a true HR ≤ 1, which corresponds to a true *ln* (HR) ≤ 0. In general, for any desired threshold *D*, we set *T* = (*ln* (D) – *ln* (μ))/SD_PI_ and df = 4, and computed the following *P* values using this website [https://www.danielsoper.com/statcalc/calculator.aspx?id=8]: *P* (true HR ≤ 1) = 0.99993; *P* (true HR ≤ 0.7133) = 0.95001; *P* (true HR ≤ 0.673) = 0.5.

### Cell culture

Human primary pre-adipocytes isolated from subcutaneous adipose tissue from nondiabetic or type 2 diabetic donors were purchased from Lonza. Cells at passage 2–4 were used in this study. Pre-adipocytes were seeded in 96-well or 6-well plates and cultured in Preadipocyte Growth Medium-2 basic medium (Lonza) supplemented with 10% FBS, L-glutamine, and gentamycin (Lonza, PT-9502) and maintained in at 37 °C, 5% CO_2_ for 5–7 days until reaching 70% confluence. Cells were then exposed to differentiation medium (Preadipocyte Growth Medium-2 supplemented as described by Lonza, including recombinant insulin, dexamethasone, indomethacin, isobutylmethylxanthine, and indomethacin) for 5–7 days to induce maturation, which was monitored morphologically. Human primary skeletal myoblasts (HSM) isolated from the upper arm muscle tissue were obtained from Zenbio or Lonza. HSM were obtained from nondiabetic or type 2 diabetic donors. HSM were utilized at passage 4–5 in this study. HSM were seeded in 96-well or 6-well plates and cultured in SKM-M (Zenbio) basic medium as described by the manufacturer. Cells were maintained in at 37 °C, 5% CO_2_ for 2–3 days until reaching 70% confluence. HSM were then exposed to differentiation medium (#SKM-D, Zenbio) for another 6–8 days to induce maturation, as assessed morphologically.

Mature adipocytes or HSM seeded in 6-well plates were treated with NRTIs (lamivudine (L1295 from Sigma-Aldrich), azidothymidine (A2169 from Sigma-Aldrich), stavudine (D1413 from Sigma-Aldrich); optimal dose of 100 μM and duration of 1 h selected from pilot experiments), 2.5 nM TNF (human recombinant, #T0157-10UG from Sigma-Aldrich), 25 mM glucose (G5500-500MG from Sigma-Aldrich), 100 nM insulin (human recombinant, #12585014 from ThermoFisher Scientific).

### Animals

All animal studies were approved by the University of Virginia Animal Care and Use Committee and performed according to their guidelines. Male 12-week-old C57BL/6 J mice (The Jackson Laboratory, JAX stock #000664) were housed in specific pathogen-free conditions and maintained under a 12-h light-dark cycle and fed ad libitum with a standard laboratory diet or a high-fat diet paste containing 60% fat plus 0.2% cholesterol (Bioserv) for 8 weeks. Animals were housed in the same room and their care and housing were in accordance with the guidelines and rules of the Institutional Animal Care and Use Committee. High-fat diet-fed mice were administered intraperitoneal injections of lamivudine (70 mg/kg of body-weight once daily) or of phosphate-buffered saline (vehicle control). Euthanasia was performed as a two-step process by inhalation of carbon dioxide gas followed by cervical dislocation.

### Western blotting

Human cells and various mouse tissue protein lysates were homogenized in Complete Lysis Buffer (Roche). Protein concentrations were measured using Pierce BCA protein Assay Kit^®^ (ThermoFisher Scientific). Proteins were separated by either 4–20% or 10–20% sodium dodecyl sulfate polyacrylamide gel electrophoresis and transferred to polyvinylidene difluoride membranes, which were then probed with specific primary antibodies. The abundance of AKT (phosphorylated and total), DICER1, and IL-18 proteins was monitored in human adipocytes, human myocytes, mouse subcutaneous adipose tissue, mouse visceral adipose tissues, and mouse skeletal muscle by western blotting using the following primary antibodies: Mouse anti-human phospho-specific AKT, Ser473 (#12694, Cell Signaling Technology; 1:1000); rabbit anti-mouse AKT (pan), 11E7 (#4685, Cell Signaling Technology; 1:1000); rabbit anti-human DICER1 A301-936A (Bethyl Laboratories; 1:1000); rat anti-mouse IL-18 (Clone 39-3 F, #D046-3, MBL International; 1:1000); anti-mouse β-actin (8H10D10) (#3700, Cell Signaling Technology; 1:1000; for loading control assessment). Following incubation with secondary antibodies, protein abundance was visualized using the Licor Odyssey documentation system and quantitated using ImageJ Fiji software, version 2.1.0/1.53c.

### Northern blotting

Total RNA from primary human adipocytes, primary human skeletal muscle cells, mouse adipose tissue, and mouse skeletal muscle tissue was extracted using Trizol (Thermo Fisher Scientific). RNA samples were separated on 10% or 15% TBE-urea gels (Bio-Rad Laboratories) according to the manufacturer’s instructions. Samples were transferred and cross-linked using ultraviolet light to a HyBond N+ nylon membrane, and blotted for *Alu* RNA, B2 RNA or 5.8S rRNA using biotinylated oligonucleotide probes. Blots were developed with the Thermo Pierce chemiluminescent nucleic acid detection kit (ThermoFisher Scientific).

### IL-1β ELISA

To measure IL-1β levels in mouse adipose and skeletal muscle tissues, we used a monoclonal antibody-based sandwich ELISA (ThermoFisher Scientific) according to the manufacturer′s instructions.

### In vitro glucose uptake in human adipocytes and myocytes

From type 2 diabetic patients and nondiabetic donors, preadipocytes were isolated from subcutaneous adipose tissue and skeletal myoblasts from the upper arm muscle tissue (Zenbio and Lonza). Glucose uptake assay was performed according to the manufacturer’s instructions (Cayman Chemical). Briefly, pre-adipocytes or human primary skeletal myoblasts were seeded in 96-well plates and induced to mature adipocytes or myocytes for 5–8 days. Nondiabetic cells were rendered insulin resistant by treatment with human tumor necrosis factor (TNF) (2.5 nM) or with high glucose (25 mM) and high insulin (100 nM) for 24 h^87,88^. Cells, pre-treated with lamivudine (100 μM) or vehicle for 1 h, were treated with insulin (20 nM) for 20 min in 100 μl glucose-free culture medium containing 2-NBDG (150–300 μg/ml; Cayman Chemical), a fluorescent derivative of glucose used to monitor glucose uptake. At the end of the treatment, the plate was centrifuged for 5 min at 400 g and washed twice with cell-based assay buffer, and read at 485/535 nm.

### Cell viability

Cell viability measurements were performed using the CellTiter 96 AQueous One Solution Cell Proliferation Assay (Promega) according to the manufacturer’s instructions. Briefly, human adipocytes were seeded on a 96-well plate and treated with 100 µM NRTI (lamivudine, 3TC; stavudine, D4T; azidothymidine, AZT) or phosphate-buffered saline (PBS; Ctrl) for 24 h. Then, 20 μl of CellTiter 96 AQueous One solution reagent was added into each well. Then, the 96-well assay plate was incubated at 37 °C for 2 h. Final absorbance reading was performed by Cytation 5 Cell Imaging Multi-Mode Reader (BioTek) at 490 nm.

### Glucose tolerance test and insulin tolerance test in mice

Male 12-week-old C57BL/6 J mice (The Jackson Laboratory) were fed with a standard laboratory diet or a high-fat diet paste with 60% fat plus 0.2% cholesterol (Bio-Serv) for 8 weeks. High-fat diet-fed mice were administered lamivudine (70 mg/kg of body weight) or phosphate-buffered saline via intraperitoneal injection once daily for 8 weeks. The glucose tolerance test was performed after a fast for 16–18 h followed by an injection of glucose (2.5 g/kg body weight; Sigma), and the insulin tolerance test after a fast for 4 h followed by an injection of insulin (0.75 U/kg body weight; Sigma). Blood glucose was monitored in tail vein blood by One-Touch Ultra (Life Scan) Glucometer at 0, 15, 30, 60, 90 and 120 min after glucose or insulin injection. Areas under the curve (AUCs) were calculated using trapezoidal integration.

### Protein and RNA assays in human cells and mouse tissues

Levels of AKT (phosphorylated and total), DICER1, and IL-18 in human cells or mouse tissues, were monitored using western blotting. To measure IL-1β levels in tissue samples obtained from mice, we used a monoclonal antibody-based sandwich ELISA (ThermoFisher Scientific). To assess the abundance of *Alu* RNA in human cells and B2 RNA in mouse tissues, we performed northern blotting^20^.

### Statistical analysis for in vitro and in vivo experiments

Data are expressed as means ± SEM. Statistical significance was determined by Student *t* test, using Prism, version 8.3.0 (Graphpad). *P* values less than 0.05 were considered significant.

### Reporting summary

Further information on research design is available in the Nature Research Reporting Summary linked to this article.

## Supplementary information

Supplementary Information

Reporting Summary

## Data Availability

The experimental data that support this study are available from the corresponding author upon reasonable request. Analyses of the Veterans Health Administration Database were performed using data within the US Department of Veterans Affairs secure research environment, the VA Informatics and Computing Infrastructure (VINCI). The other health insurance datasets are subject to licensing agreements and privacy restrictions. All relevant data outputs are within the paper and its supplemental information. Researchers interested in accessing data are encouraged to make direct enquiries to the corresponding author and should note they may also need to approach Truven MarketScan, PearlDiver, Clinformatics, and the Centers for Medicare & Medicaid Services for access to data from these sources. Source data are provided with this paper.

## Source data

Source Data

## Code availability

Analyses were conducted using standard procedures without special code development. Code for meta-analyses is available at https://github.com/macavsma/CodeforNC/.

## References

[1] International Diabetes Federation. *IDF Diabetes Atlas*. 9th edn, (International Diabetes Federation, 2019).

[2] Kahn SE, Cooper ME, Del Prato S (2014). Pathophysiology and treatment of type 2 diabetes: perspectives on the past, present, and future. Lancet.

[3] Pickup JC, Crook MA (1998). Is type II diabetes mellitus a disease of the innate immune system?. Diabetologia.

[4] Donath MY, Shoelson SE (2011). Type 2 diabetes as an inflammatory disease. Nat. Rev. Immunol..

[5] Masters SL, Latz E, O’Neill LA (2011). The inflammasome in atherosclerosis and type 2 diabetes. Sci. Transl. Med..

[6] Vandanmagsar B (2011). The NLRP3 inflammasome instigates obesity-induced inflammation and insulin resistance. Nat. Med..

[7] Masters SL (2010). Activation of the NLRP3 inflammasome by islet amyloid polypeptide provides a mechanism for enhanced IL-1beta in type 2 diabetes. Nat. Immunol..

[8] Wen H (2011). Fatty acid-induced NLRP3-ASC inflammasome activation interferes with insulin signaling. Nat. Immunol..

[9] Martinon F, Burns K, Tschopp J (2002). The inflammasome: a molecular platform triggering activation of inflammatory caspases and processing of proIL-beta. Mol. Cell.

[10] Zhou R, Tardivel A, Thorens B, Choi I, Tschopp J (2010). Thioredoxin-interacting protein links oxidative stress to inflammasome activation. Nat. Immunol..

[11] Youm YH (2011). Elimination of the NLRP3-ASC inflammasome protects against chronic obesity-induced pancreatic damage. Endocrinology.

[12] Stienstra R (2011). Inflammasome is a central player in the induction of obesity and insulin resistance. Proc. Natl Acad. Sci. USA.

[13] Stienstra R (2010). The inflammasome-mediated caspase-1 activation controls adipocyte differentiation and insulin sensitivity. Cell Metab..

[14] Goossens GH (2012). Expression of NLRP3 inflammasome and T cell population markers in adipose tissue are associated with insulin resistance and impaired glucose metabolism in humans. Mol. Immunol..

[15] Lee HM (2013). Upregulated NLRP3 inflammasome activation in patients with type 2 diabetes. Diabetes.

[16] Spranger J (2003). Inflammatory cytokines and the risk to develop type 2 diabetes: results of the prospective population-based European Prospective Investigation into Cancer and Nutrition (EPIC)-Potsdam Study. Diabetes.

[17] Thorand B (2005). Elevated levels of interleukin-18 predict the development of type 2 diabetes: results from the MONICA/KORA Augsburg Study, 1984-2002. Diabetes.

[18] Tarallo V (2012). DICER1 loss and Alu RNA induce age-related macular degeneration via the NLRP3 inflammasome and MyD88. Cell.

[19] Guo C (2018). Tau activates transposable elements in Alzheimer’s disease. Cell Rep..

[20] Kaneko H (2011). DICER1 deficit induces Alu RNA toxicity in age-related macular degeneration. Nature.

[21] Kerur N (2018). cGAS drives noncanonical-inflammasome activation in age-related macular degeneration. Nat. Med..

[22] Hung T (2015). The Ro60 autoantigen binds endogenous retroelements and regulates inflammatory gene expression. Science.

[23] Gelfand BD (2015). Iron toxicity in the retina requires Alu RNA and the NLRP3 inflammasome. Cell Rep..

[24] Hu Q (2012). DICER- and AGO3-dependent generation of retinoic acid-induced DR2 Alu RNAs regulates human stem cell proliferation. Nat. Struct. Mol. Biol..

[25] Elgheznawy A (2015). Dicer cleavage by calpain determines platelet microRNA levels and function in diabetes. Circ. Res..

[26] Yan Y (2013). Dicer expression exhibits a tissue-specific diurnal pattern that is lost during aging and in diabetes. PLoS ONE.

[27] Melkman-Zehavi T (2011). miRNAs control insulin content in pancreatic beta-cells via downregulation of transcriptional repressors. EMBO J..

[28] Kalis M (2011). Beta-cell specific deletion of Dicer1 leads to defective insulin secretion and diabetes mellitus. PLoS ONE.

[29] Oliverio M (2016). Dicer1-miR-328-Bace1 signalling controls brown adipose tissue differentiation and function. Nat. Cell Biol..

[30] Reis FC (2016). Fat-specific Dicer deficiency accelerates aging and mitigates several effects of dietary restriction in mice. Aging.

[31] Kazazian HH, Moran JV (2017). Mobile DNA in health and disease. N. Engl. J. Med..

[32] Dewannieux M, Esnault C, Heidmann T (2003). LINE-mediated retrotransposition of marked Alu sequences. Nat. Genet..

[33] Jones RB (2008). Nucleoside analogue reverse transcriptase inhibitors differentially inhibit human LINE-1 retrotransposition. PLoS ONE.

[34] Dai L, Huang Q, Boeke JD (2011). Effect of reverse transcriptase inhibitors on LINE-1 and Ty1 reverse transcriptase activities and on LINE-1 retrotransposition. BMC Biochem.

[35] Fowler BJ (2014). Nucleoside reverse transcriptase inhibitors possess intrinsic anti-inflammatory activity. Science.

[36] Hernan MA (2010). The hazards of hazard ratios. Epidemiology.

[37] Sohn MW, Arnold N, Maynard C, Hynes DM (2006). Accuracy and completeness of mortality data in the Department of Veterans Affairs. Popul Health Metr..

[38] Tandon N (2019). Compliance with clinical guidelines and adherence to antiretroviral therapy among patients living with HIV. Curr. Med. Res. Opin..

[39] Priest JL, Irwin DE, Evans KA, Oglesby AK, Brady BL (2020). Benchmarking HIV quality measures across US payer types. Popul. Health Manag..

[40] Jiang ZY (2003). Insulin signaling through Akt/protein kinase B analyzed by small interfering RNA-mediated gene silencing. Proc. Natl Acad. Sci. USA.

[41] Winzell MS, Ahren B (2004). The high-fat diet-fed mouse: a model for studying mechanisms and treatment of impaired glucose tolerance and type 2 diabetes. Diabetes.

[42] IntHout J, Ioannidis JP, Rovers MM, Goeman JJ (2016). Plea for routinely presenting prediction intervals in meta-analysis. BMJ Open.

[43] Collins FS (2011). Mining for therapeutic gold. Nat. Rev. Drug Disco..

[44] Brambilla AM (2003). Stavudine or indinavir-containing regimens are associated with an increased risk of diabetes mellitus in HIV-infected individuals. AIDS.

[45] De Wit S (2008). Incidence and risk factors for new-onset diabetes in HIV-infected patients: the Data Collection on Adverse Events of Anti-HIV Drugs (D:A:D) study. Diabetes Care.

[46] Paula AA, Falcao MC, Pacheco AG (2013). Metabolic syndrome in HIV-infected individuals: underlying mechanisms and epidemiological aspects. AIDS Res. Ther..

[47] Ledergerber B (2007). Factors associated with the incidence of type 2 diabetes mellitus in HIV-infected participants in the Swiss HIV Cohort Study. Clin. Infect. Dis..

[48] Jordakieva G (2019). Country-wide medical records infer increased allergy risk of gastric acid inhibition. Nat. Commun..

[49] Cheng F (2018). Network-based approach to prediction and population-based validation of in silico drug repurposing. Nat. Commun..

[50] Sutton AJ, Abrams KR (2001). Bayesian methods in meta-analysis and evidence synthesis. Stat. Methods Med. Res..

[51] Dawid AP (1982). The well-calibrated Bayesian. J. Am. Stat. Assoc..

[52] Dekker FW, de Mutsert R, van Dijk PC, Zoccali C, Jager KJ (2008). Survival analysis: time-dependent effects and time-varying risk factors. Kidney Int..

[53] Lipsitch M, Tchetgen Tchetgen E, Cohen T (2010). Negative controls: a tool for detecting confounding and bias in observational studies. Epidemiology.

[54] Prasad V, Jena AB (2013). Prespecified falsification end points: can they validate true observational associations?. JAMA.

[55] Arnold BF, Ercumen A, Benjamin-Chung J, Colford JM (2016). Brief report: negative controls to detect selection bias and measurement bias in epidemiologic studies. Epidemiology.

[56] Al-Khalidi R (2018). Zidovudine ameliorates pathology in the mouse model of Duchenne muscular dystrophy via P2RX7 purinoceptor antagonism. Acta Neuropathol. Commun..

[57] Rosenbaum PR, Rubin DB (1983). The central role of the propensity score in observational studies for causal effects. Biometrika.

[58] Haukoos JS, Lewis RJ (2015). The propensity score. JAMA.

[59] Ohlsson, H. & Kendler, K. S. Applying causal inference methods in psychiatric epidemiology: a review. *JAMA Psychiatry*, 10.1001/jamapsychiatry.2019.3758 (2019). 10.1001/jamapsychiatry.2019.3758 PMC7286775 31825494

[60] Imai K, van Dyk DA (2004). Causal inference with general treatment regimes. J. Am. Stat. Assoc..

[61] Rubin DB (2005). Causal inference using potential outcomes: design, modeling, decisions. J. Am. Stat. Assoc..

[62] Pearl J (2009). Causal inference in statistics: an overview. Stat. Surv..

[63] Schuemie MJ, Hripcsak G, Ryan PB, Madigan D, Suchard MA (2018). Empirical confidence interval calibration for population-level effect estimation studies in observational healthcare data. Proc. Natl Acad. Sci. USA.

[64] Ridruejo E, Silva MO (2012). Safety of long-term nucleos(t)ide treatment in chronic hepatitis B. Expert Opin. Drug Saf..

[65] Dienstag JL (1999). Lamivudine as initial treatment for chronic hepatitis B in the United States. N. Engl. J. Med..

[66] Jonas MM (2002). Clinical trial of lamivudine in children with chronic hepatitis B. N. Engl. J. Med..

[67] Lai CL (1998). A one-year trial of lamivudine for chronic hepatitis B. Asia Hepatitis Lamivudine Study Group. N. Engl. J. Med..

[68] Grant RM (2010). Preexposure chemoprophylaxis for HIV prevention in men who have sex with men. N. Engl. J. Med..

[69] Baeten JM (2012). Antiretroviral prophylaxis for HIV prevention in heterosexual men and women. N. Engl. J. Med..

[70] Quercia R (2018). Twenty-five years of lamivudine: current and future use for the treatment of HIV-1 infection. J. Acquir Immune Defic. Syndr..

[71] Lawlor DA, Tilling K, Davey Smith G (2016). Triangulation in aetiological epidemiology. Int. J. Epidemiol..

[72] Munafo MR, Davey Smith G (2018). Robust research needs many lines of evidence. Nature.

[73] Filippatos TD (2016). Mediterranean diet and 10-year (2002-2012) incidence of diabetes and cardiovascular disease in participants with prediabetes: the ATTICA study. Rev. Diabet. Stud..

[74] Hrubeniuk, T. J., Bouchard, D. R., Goulet, E. D. B., Gurd, B. & Senechal, M. The ability of exercise to meaningfully improve glucose tolerance in people living with prediabetes: a meta-analysis. *Scand J. Med. Sci. Sports*, 10.1111/sms.13567 (2019). 10.1111/sms.13567 31593613

[75] Senechal M, Slaght J, Bharti N, Bouchard DR (2014). Independent and combined effect of diet and exercise in adults with prediabetes. Diabetes Metab. Syndr. Obes..

[76] National Institute of Diabetes and Digestive and Kidney Diseases (NIDDK). *Risk Factors for Type 2 Diabetes*, https://www.niddk.nih.gov/health-information/diabetes/overview/risk-factors-type-2-diabetes. 38117928

[77] Centers for Disease Control and Prevention. *Who’s at Risk?*, https://www.cdc.gov/diabetes/basics/risk-factors.html.

[78] Moreau T, O’Quigley J, Lellouch J (1986). On D. Schoenfeld’s approach for testing the proportional hazards assumption. Biometrika.

[79] Schoenfeld D (1980). Chi-squared goodness-of-fit tests for the proportional hazards regression model. Biometrika.

[80] Higgins JP, Thompson SG (2002). Quantifying heterogeneity in a meta-analysis. Stat. Med..

[81] Anello, C. & Fleiss, J. L. Exploratory or analytic meta-analysis: should we distinguish between them? *J. Clin. Epidemiol.* **48**, 109-116; discussion 117-108 (1995). 10.1016/0895-4356(94)00084-4 7853037

[82] DerSimonian R, Laird N (1986). Meta-analysis in clinical trials. Control Clin. Trials.

[83] Lau J, Ioannidis JP, Schmid CH (1998). Summing up evidence: one answer is not always enough. Lancet.

[84] Spiegelhalter, D. J., Abrams, K. R. & Myles, J. P. *Bayesian approaches to clinical trials and health-care evaluation*. (John Wiley & Sons, 2004).

[85] Polson NG, Scott JG (2012). On the half-Cauchy prior for a global scale parameter. Bayesian Anal..

[86] Gelman A (2006). Prior distributions for variance parameters in hierarchical models. Bayesian Anal..

[87] Henry RR, Ciaraldi TP, Mudaliar S, Abrams L, Nikoulina SE (1996). Acquired defects of glycogen synthase activity in cultured human skeletal muscle cells: influence of high glucose and insulin levels. Diabetes.

[88] Hotamisligil GS (1996). IRS-1-mediated inhibition of insulin receptor tyrosine kinase activity in TNF-alpha- and obesity-induced insulin resistance. Science.

