# Supplementary material for: Repurposing anti-inflammasome NRTIs for improving insulin sensitivity and reducing type 2 diabetes development
Source: Nat Commun. 2020 Sep 23;11:4737. doi: 10.1038/s41467-020-18528-z (PMC7511405; doi:10.1038/s41467-020-18528-z)
Supplement: Supplementary file 1 — Supplementary Information [file 41467_2020_18528_MOESM1_ESM.pdf]

Supplementary Information

Repurposing NRTIs for inhibiting inflammasome, insulin resistance, and type 2 diabetes development

Ambati et al.

This PDF file includes:

Supplementary Figs. S1 to S17
Supplementary Tables 1 to 31
Materials and Methods
References

Supplementary Figure 1. Flow Diagrams of Cohort Selection in the 5 Databases.

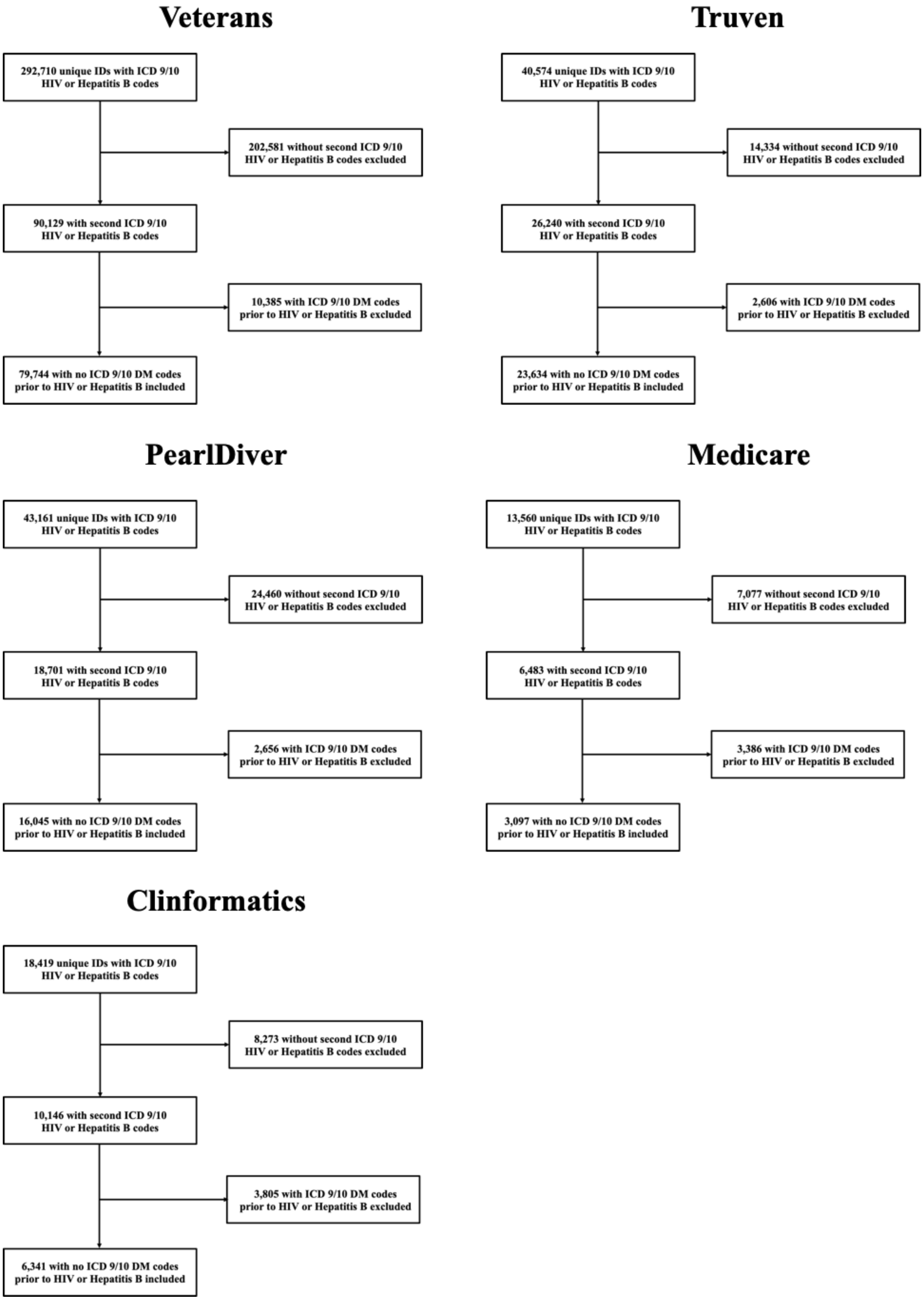

Supplementary Figure 2. Adjusted Survival Curves in the Veterans Health Administration Database.

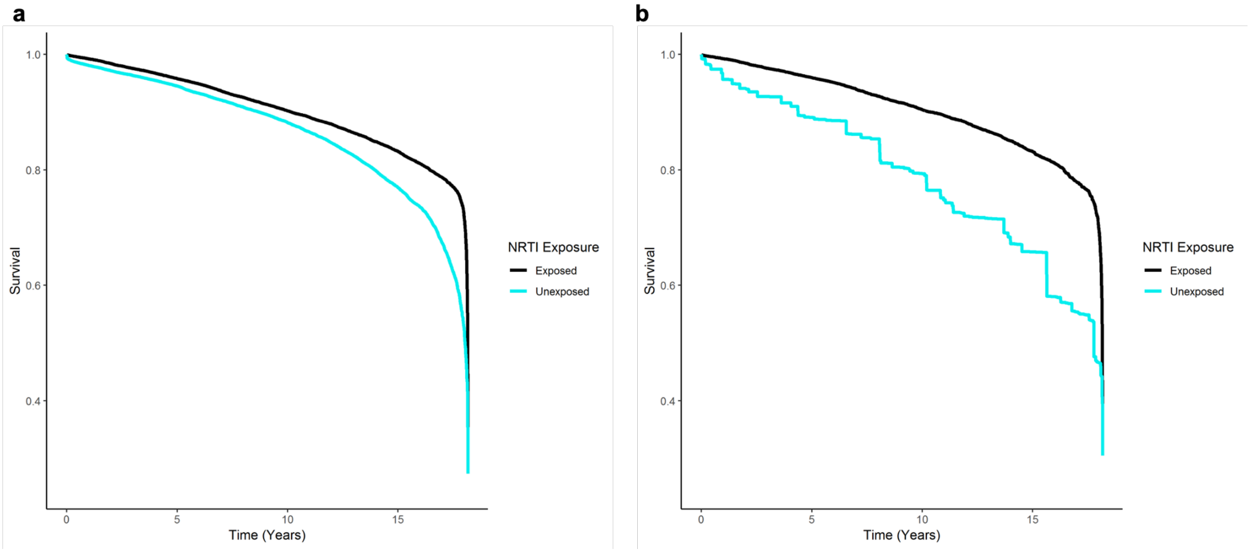

a, b Unadjusted (**a**) and adjusted (inverse probability weighted) survival curves (**b**) are presented for Veterans Health Administration database participants, calculated for each level of NRTI exposure (exposed/unexposed). These curves present expected survival (remaining free of type 2 diabetes) over time calculated for the NRTI exposure levels as fit from the exposed / unexposed Cox proportional hazards model.

Supplementary Figure 3. Forest Plot of Incident Diabetes and NRTI exposure (Per Year of Exposure)

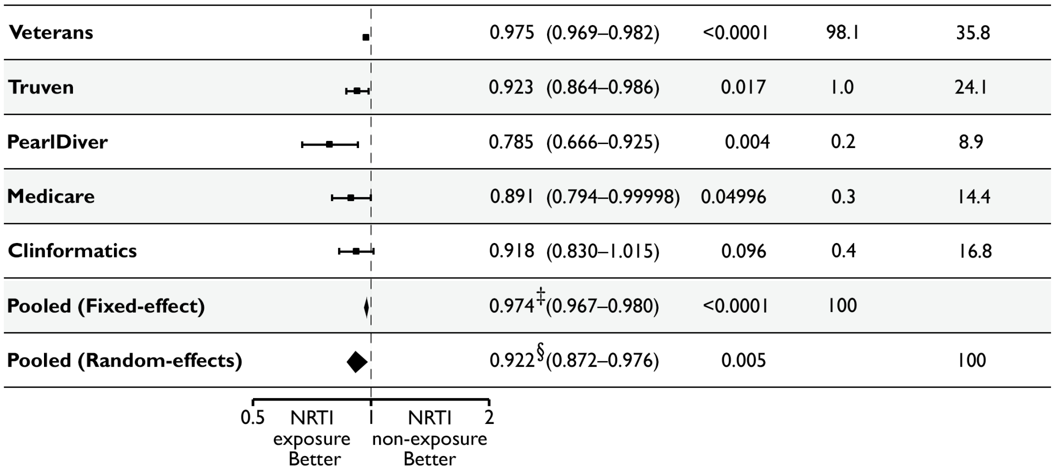

	Adjusted Hazard Ratio Per Year of NRTI Exposure (95% CI)	P value	Weight, % Fixed-effect*	Weight, % Random-effects†
**Veterans**	0.975 (0.969–0.982)	<0.0001	98.1	35.8
**Truven**	0.923 (0.864–0.986)	0.017	1.0	24.1
**PearlDiver**	0.785 (0.666–0.925)	0.004	0.2	8.9
**Medicare**	0.891 (0.794–0.99998)	0.04996	0.3	14.4
**Clinformatics**	0.918 (0.830–1.015)	0.096	0.4	16.8
**Pooled (Fixed-effect)**	0.974‡ (0.967–0.980)	<0.0001	100	
**Pooled (Random-effects)**	0.922§ (0.872–0.976)	0.005		100

*Influence of studies on meta-analysis using fixed-effect model
†Influence of studies on meta-analysis using random-effects model
Test for heterogeneity: χ^2=12.87 df=4, P=0.0119; τ^2=0.0023; I^2=65.7%; 95% CI, 9.7% to 97.6%
‡Test for overall effect: z=7.95, P<0.0001
§Test for overall effect: z=2.81, P=0.005

Forest Plot of Incident Diabetes and NRTI exposure using Random-Effects and Fixed-Effect Models. Hazard ratios based on a Cox proportional-hazards model and adjusted for the confounding variables listed in Supplementary Tables 5, 7, 9, 11, and 13 were estimated separately for each database. The dashed vertical line denotes a hazard ratio of 1.0, which represents no difference in risk between nucleoside reverse-transcriptase inhibitor (NRTI) exposure and non-exposure. P values derived from z tests for individual databases are reported. Inverse-variance weighted random-effects and fixed-effect meta-analysis were performed to obtain a pooled estimate of the adjusted hazard ratio of incident diabetes per year of NRTI exposure. The estimate of heterogeneity (τ^2) and the results of the statistical test of heterogeneity using the chi-square (χ^2) test statistic and its degrees of freedom (df) are shown below the plot. The Higgins I^2 statistic and its 95% CI are presented. The results of the statistical tests of overall effect, the z test statistics, and corresponding P values are presented. All tests were two-tailed.

Supplementary Figure 4. Forest Plot of Incident Appendicitis and NRTI exposure.

	Adjusted Hazard Ratio Per Year of NRTI Exposure (95% CI)	P value	Weight, % Fixed-effect*	Weight, % Random-effects†
**Veterans**	0.985 (0.966–1.005)	0.13	63.5	60.6
**Truven**	0.902 (0.743–1.095)	0.30	0.7	0.9
**PearlDiver**	1.004 (0.978–1.031)	0.77	35.7	38.4
**Medicare**	0.383 (0.101–1.445)	0.16	0.01	0.02
**Clinformatics**	1.376 (0.844–2.244)	0.20	0.1	0.1
**Pooled (Fixed-effect)**	0.991‡ (0.976–1.007)	0.28	100	
**Pooled (Random-effects)**	0.992§ (0.974–1.010)	0.37		100

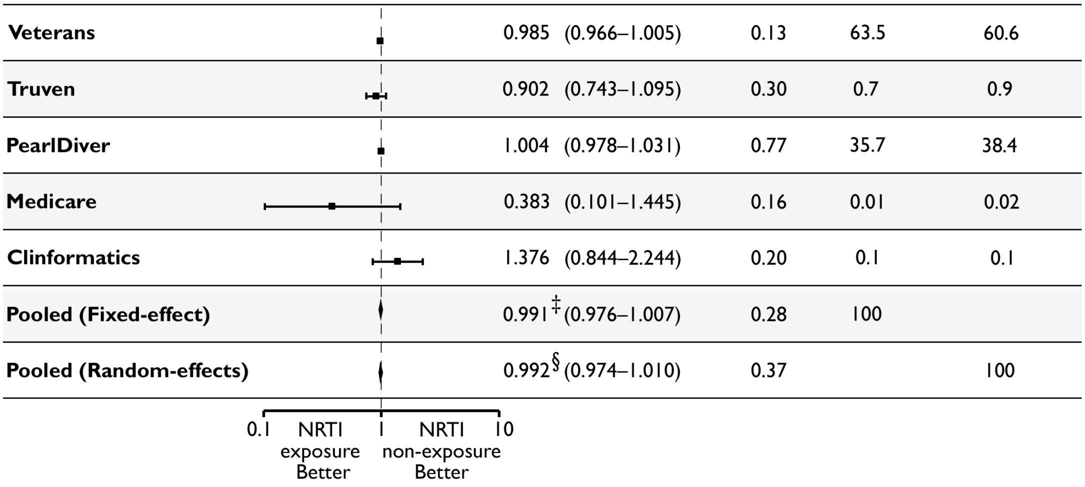

*Influence of studies on meta-analysis using fixed-effect model

†Influence of studies on meta-analysis using random-effects model

Test for heterogeneity: χ^2=5.89 df=4, P=0.21; τ^2<0.0001; I^2=6.0%; 95% CI, 0% to 100%

‡Test for overall effect: z=1.08, P=0.28

§Test for overall effect: z=0.91, P=0.37

Forest Plot of Incident Appendicitis and NRTI exposure using Random-Effects and Fixed-Effect Models. Hazard ratios based on a Cox proportional-hazards model and adjusted for the confounding variables listed in Supplementary Tables 5, 7, 9, 11, and 13 were estimated separately for each database. The dashed vertical line denotes a hazard ratio of 1.0, which represents no difference in risk between nucleoside reverse-transcriptase inhibitor (NRTI) exposure and non-exposure. P values derived from z tests for individual databases are reported. Inverse-variance weighted random-effects and fixed-effect meta-analysis were performed to obtain a pooled estimate of the adjusted hazard ratio of incident appendicitis per year of NRTI exposure. The estimate of heterogeneity (τ^2) and the results of the statistical test of heterogeneity using the chi-square (χ^2) test statistic and its degrees of freedom (df) are shown below the plot. The Higgins I^2 statistic and its 95% CI are presented. The results of the statistical tests of overall effect, the z test statistics, and corresponding P values are presented. All tests were two-tailed.

Supplementary Figure 5. Forest Plot of Incident Hernia and NRTI exposure.

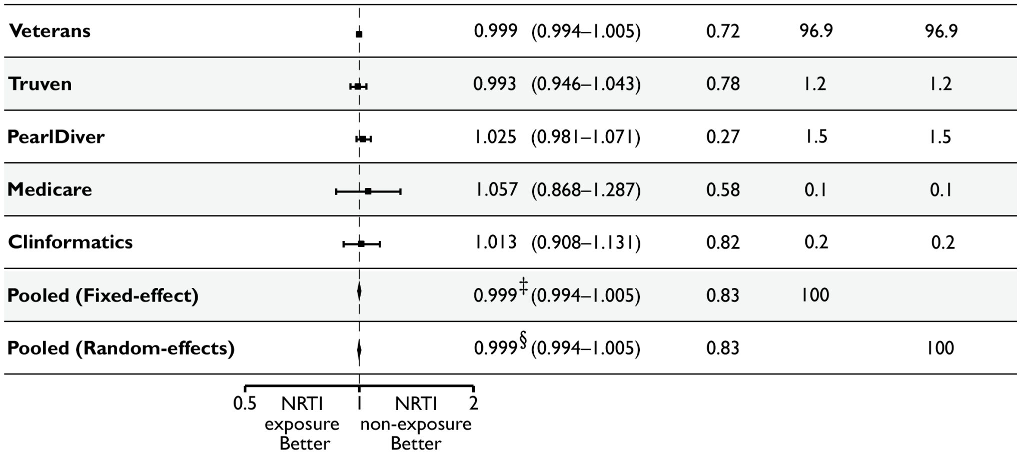

	Adjusted Hazard Ratio Per Year of NRTI Exposure (95% CI)	P value	Weight, % Fixed-effect*	Weight, % Random-effects†
**Veterans**	0.999 (0.994–1.005)	0.72	96.9	96.9
**Truven**	0.993 (0.946–1.043)	0.78	1.2	1.2
**PearlDiver**	1.025 (0.981–1.071)	0.27	1.5	1.5
**Medicare**	1.057 (0.868–1.287)	0.58	0.1	0.1
**Clinformatics**	1.013 (0.908–1.131)	0.82	0.2	0.2
**Pooled (Fixed-effect)**	0.999‡ (0.994–1.005)	0.83	100	
**Pooled (Random-effects)**	0.999§ (0.994–1.005)	0.83		100

*Influence of studies on meta-analysis using fixed-effect model
†Influence of studies on meta-analysis using random-effects model
Test for heterogeneity: χ^2=1.73 df=4, P=0.78, τ^2=0.0; I^2=0.0%; 95% CI, 0% to 75.6%
‡Test for overall effect: z=0.22, P=0.83
§Test for overall effect: z=0.22, P=0.83

Forest Plot of Incident Hernia and NRTI exposure using Random-Effects and Fixed-Effect Models. Hazard ratios based on a Cox proportional-hazards model and adjusted for the confounding variables listed in Supplementary Tables 5, 7, 9, 11, and 13 were estimated separately for each database. The dashed vertical line denotes a hazard ratio of 1.0, which represents no difference in risk between nucleoside reverse-transcriptase inhibitor (NRTI) exposure and non-exposure. P values derived from z tests for individual databases are reported. Inverse-variance weighted random-effects and fixed-effect meta-analysis were performed to obtain a pooled estimate of the adjusted hazard ratio of incident hernia per year of NRTI exposure. The estimate of heterogeneity (τ^2) and the results of the statistical test of heterogeneity using the chi-square (χ^2) test statistic and its degrees of freedom (df) are shown below the plot. The Higgins I^2 statistic and its 95% CI are presented. The results of the statistical tests of overall effect, the z test statistics, and corresponding P values are presented. All tests were two-tailed.

Supplementary Figure 6. Distribution of the Logit of the Propensity Score – Veterans Health Administration Database.

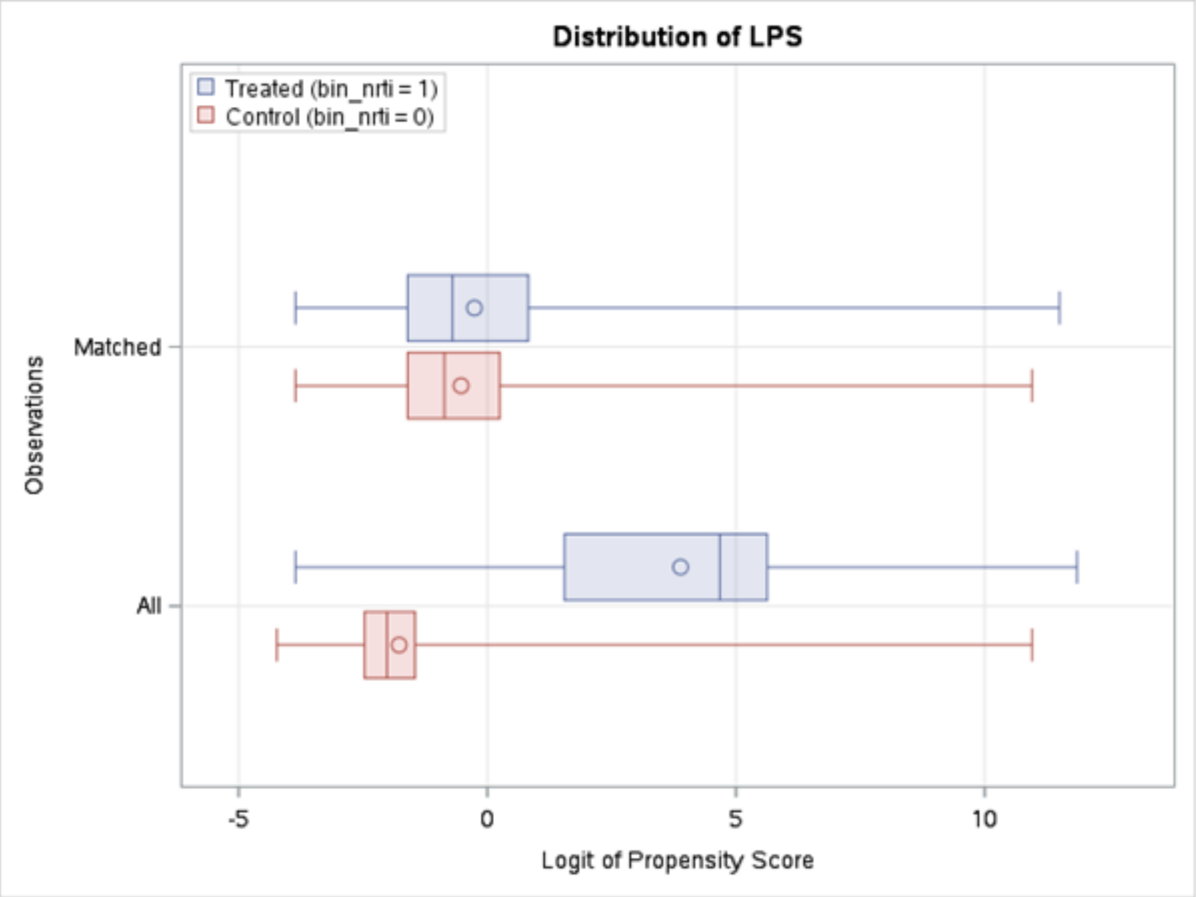

Box plots for the logit of the propensity score (LPS) show good balance for the matched observations. The box represents the 1st quartile (left side), median(vertical line inside the box), mean (circle) and the 3rd quartile (right side). Lines extending on either side of the box span the minimum and maximum values. N=9,057 in each of the matched cohorts, N=37,227 among all NRTI-unexposed patients and N=41,517 among all NRTI-exposed patients.

Supplementary Figure 7. Cloud plot: Logit of the propensity score support region – Veterans Health Administration Database.

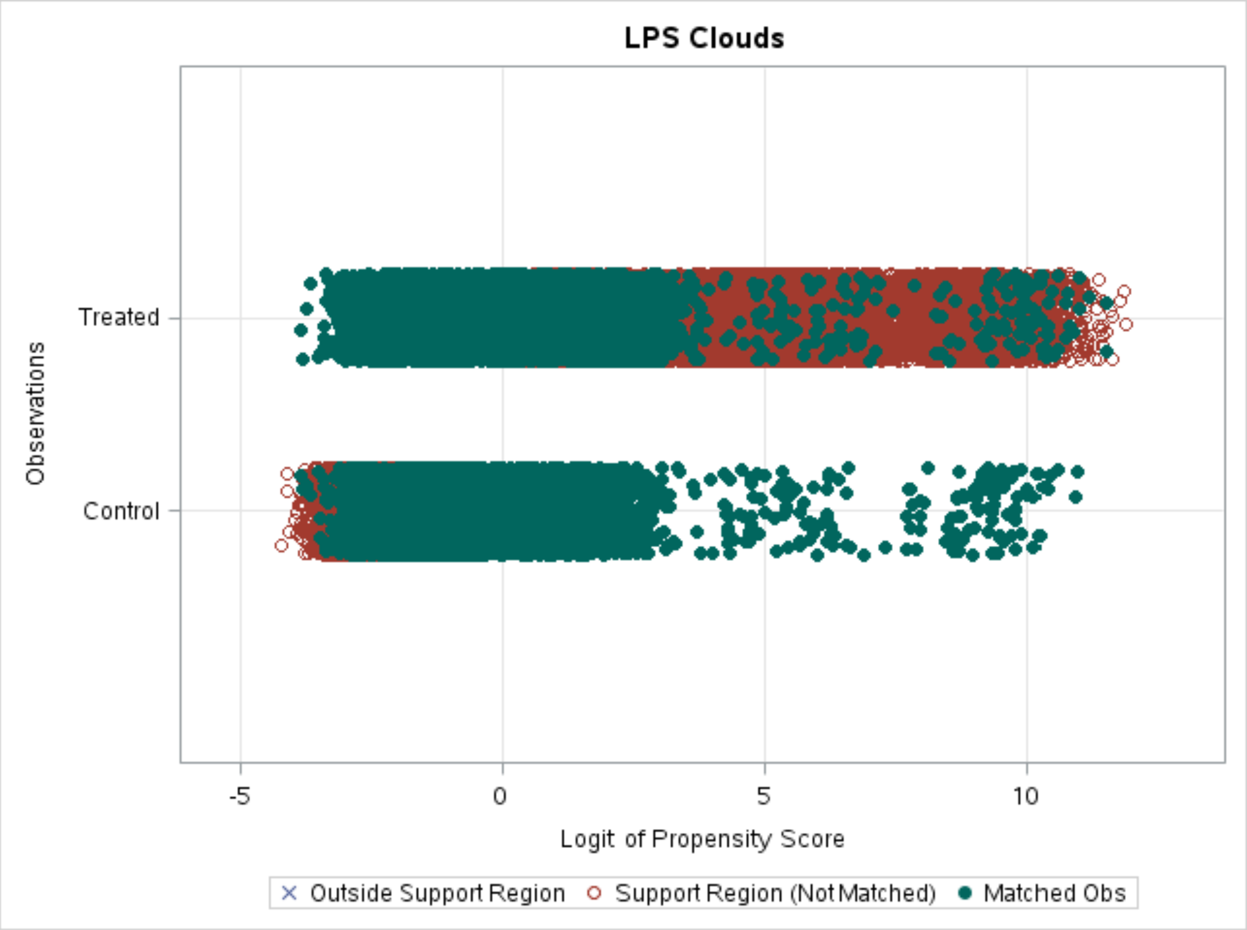

Cloud plot shows the distribution of the logit of the propensity scores for the treated (NRTI exposed) and control (NRTI non-exposed) patients and the degree to which these distributions overlap. Green dots represent patients who are successfully matched with a patient in the opposite group with a similar propensity score. Red circles and blue x's represent patients for whom a match is not available.

Supplementary Figure 8. Distribution of the Logit of the Propensity Score – Truven Marketscan Database.

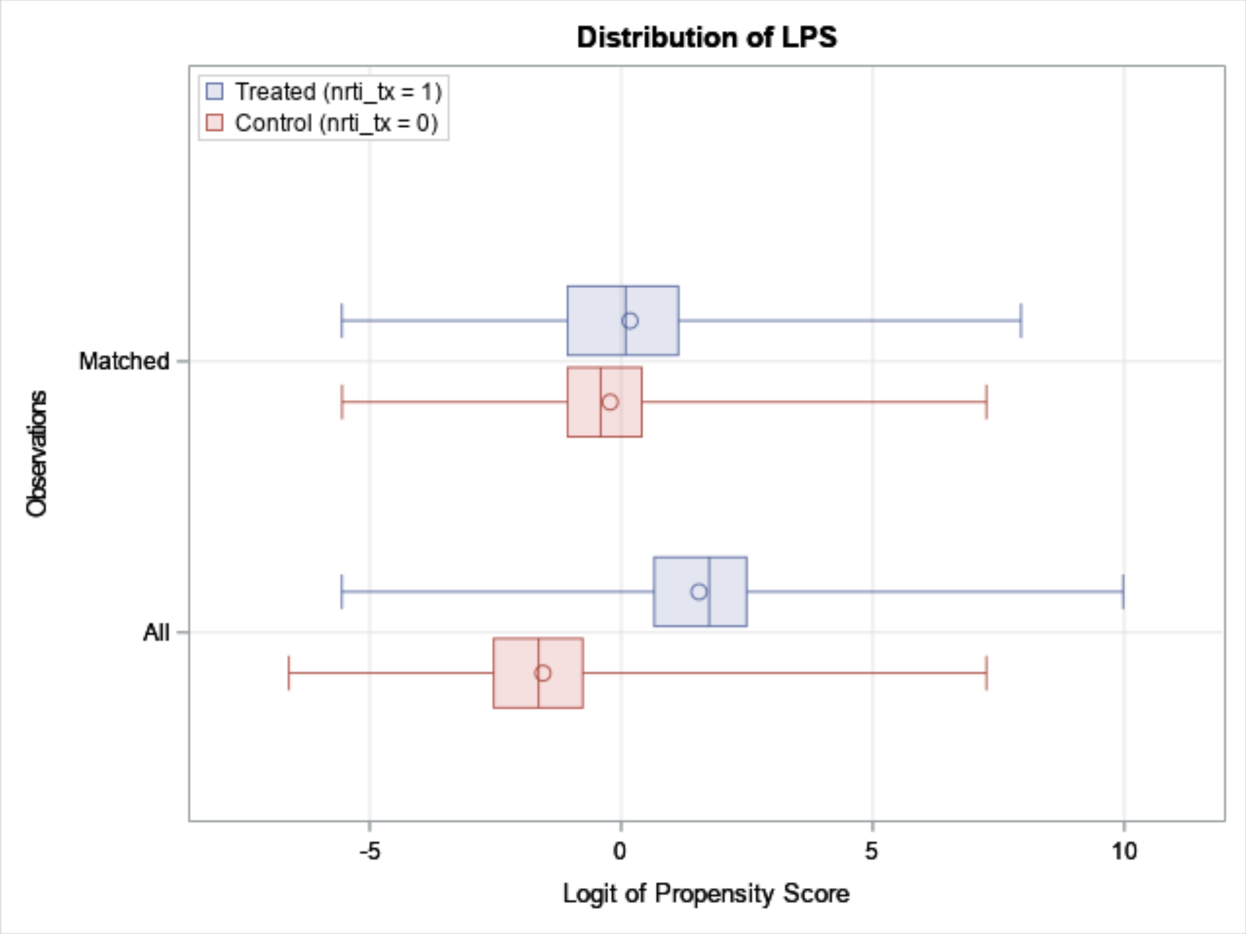

Box plots for the logit of the propensity score (LPS) show good balance for the matched observations. The box represents the 1st quartile (left side), median(vertical line inside the box), mean (circle) and the 3rd quartile (right side). Lines extending on either side of the box span the minimum and maximum values. N=4,343 in each of the matched cohorts, N=12,308 among all NRTI-unexposed patients and N=11,326 among all NRTI-exposed patients.

Supplementary Figure 9. Cloud plot: Logit of the propensity score support region – Truven Marketscan Database.

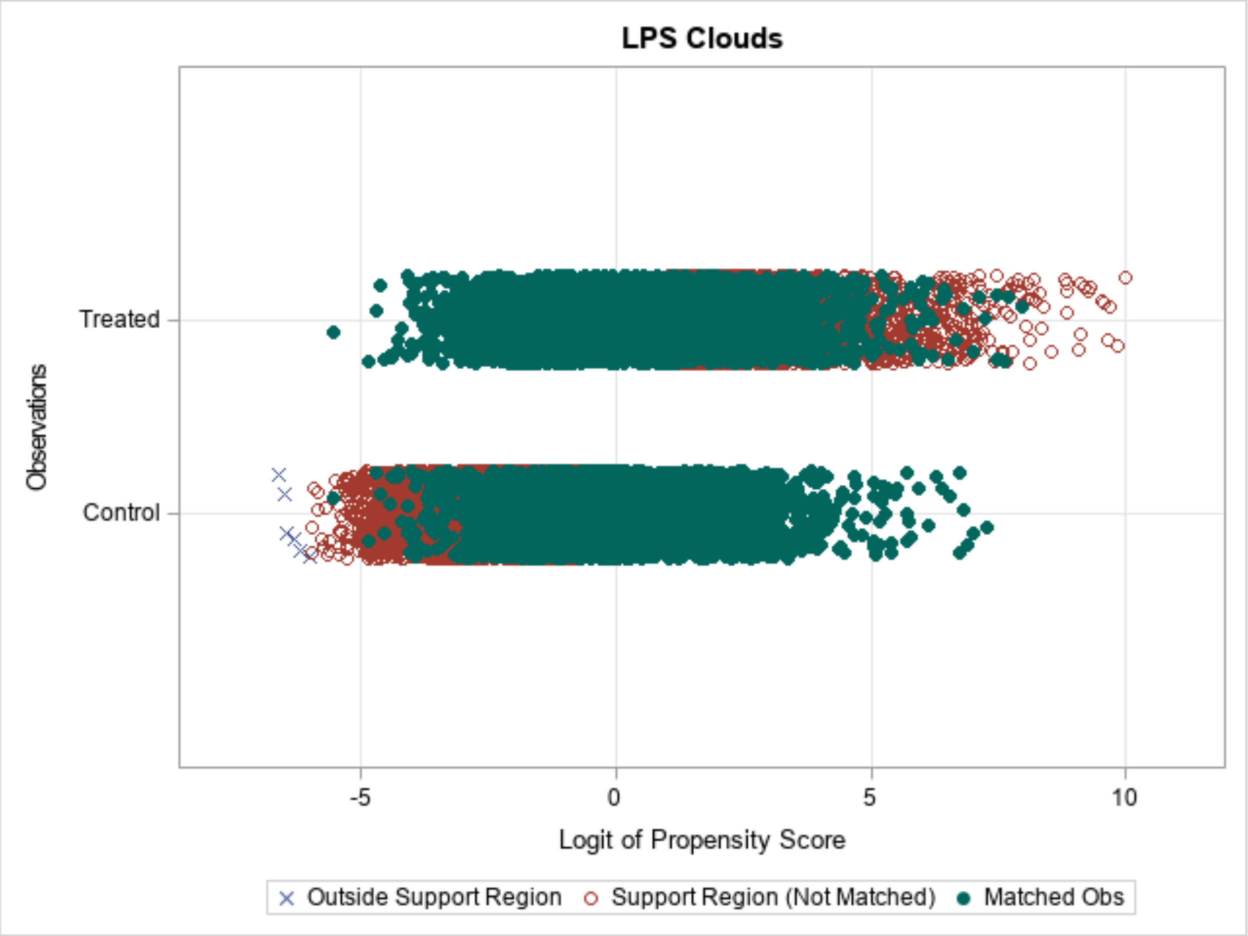

Cloud plot shows the distribution of the logit of the propensity scores for the treated (NRTI exposed) and control (NRTI non-exposed) patients and the degree to which these distributions overlap. Green dots represent patients who are successfully matched with a patient in the opposite group with a similar propensity score. Red circles and blue x's represent patients for whom a match is not available.

Supplementary Figure 10. Distribution of the Propensity Scores in PearlDiver Database.

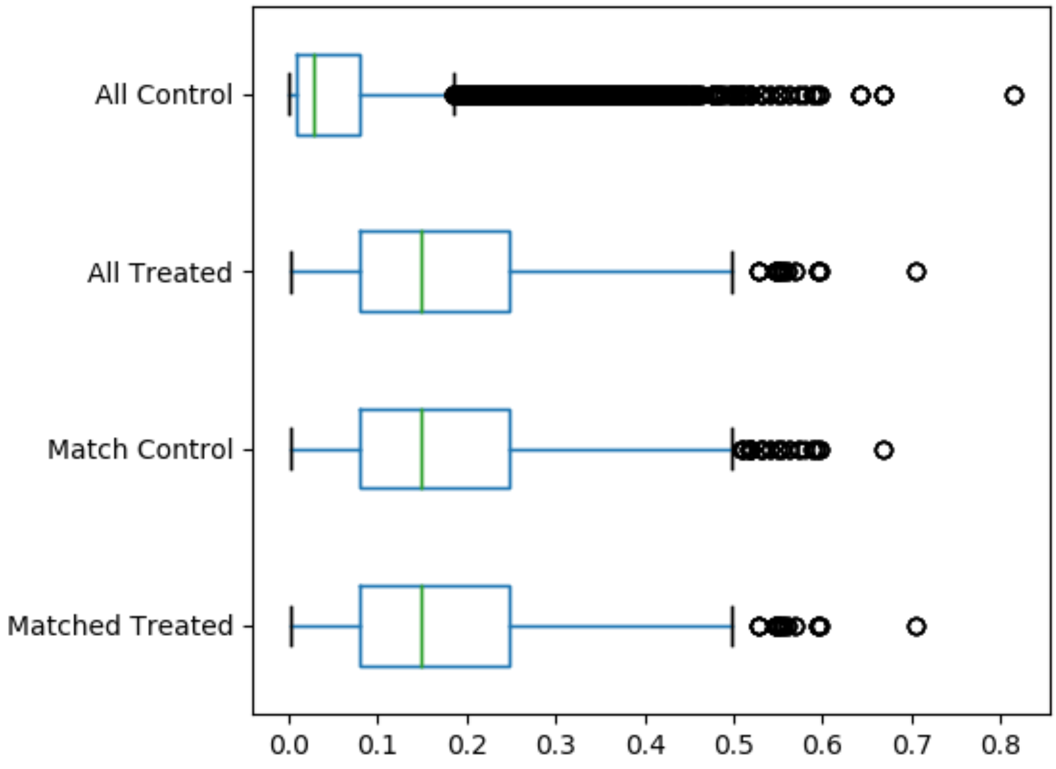

Box plots for the propensity score show good balance for the matched observations. Fliers are shown as open circles. The box represents the 1st quartile (left side), median(vertical line inside the box), mean (circle) and the 3rd quartile (right side). Lines extending on either side of the box are 1.5-times the interquartile range. N=2,153 in each of the matched cohorts, N=8,513 among all NRTI-unexposed patients and N=11,838 among all NRTI-exposed patients.

Supplementary Figure 11. Cloud plot: Propensity scores for matched and unmatched populations in PearlDiver Database.

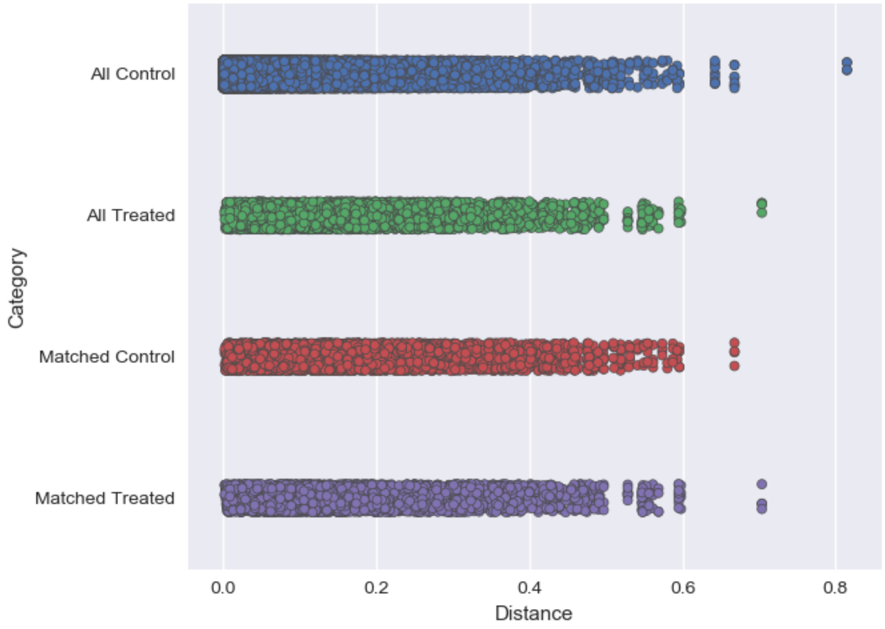

Cloud plot shows the distribution of the propensity scores (distance) for the treated (NRTI exposed) and control (NRTI non-exposed) patients and the degree to which these distributions overlap.

Supplementary Fig. 12. AKT phosphorylation in human adipocytes and skeletal myocytes.

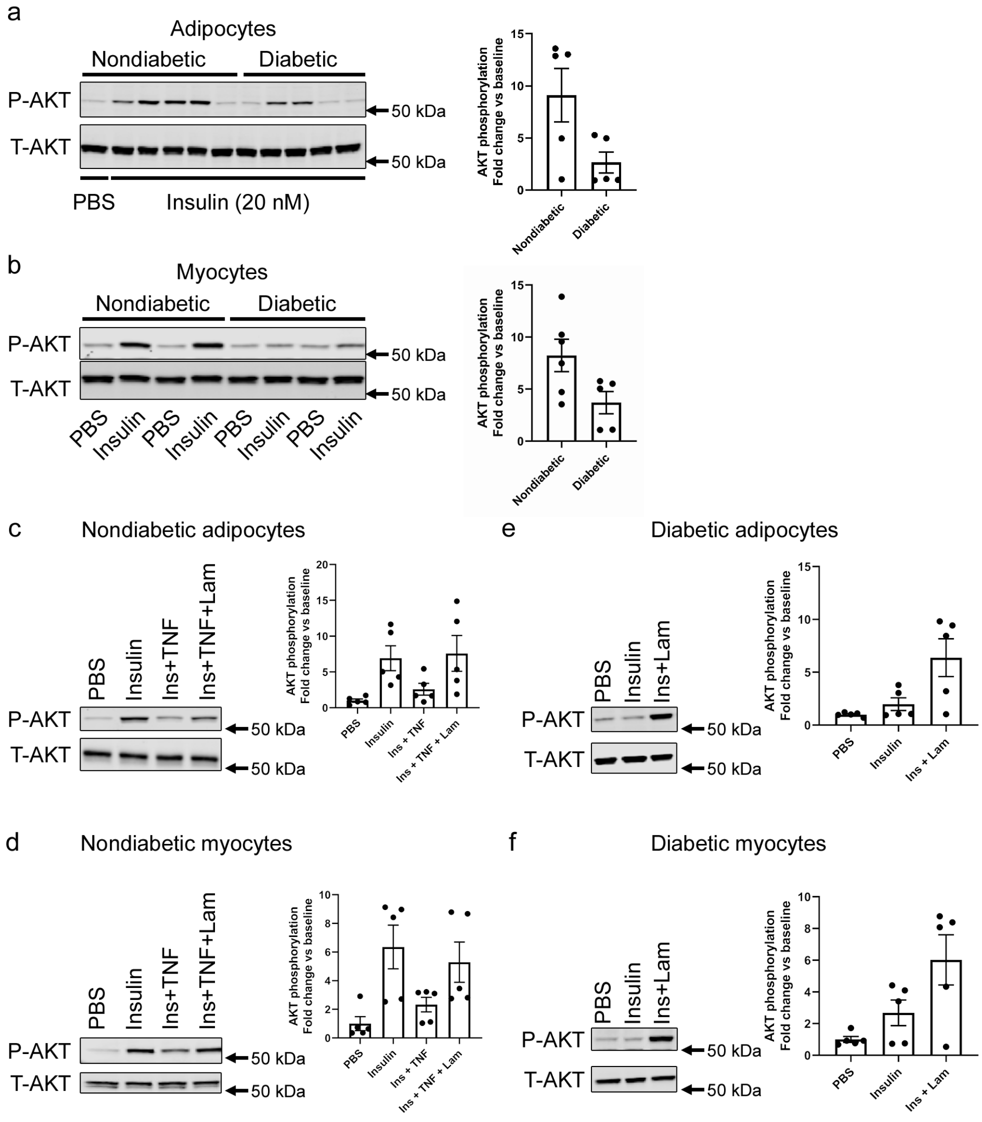

a, b The left panels show the results of western blotting of extracts of proteins from nondiabetic and diabetic human adipocytes (**a**) and myocytes (**b**) treated with phosphate-buffered saline (PBS) or insulin (20 nM). Immunoreactive bands corresponding to phosphorylated AKT (P-AKT) and total AKT (T-AKT) are shown. The right panels show bar graphs of the densitometric analyses of the ratio of P-AKT to T-AKT abundance in these western blots in the insulin-treated samples compared to that in the PBS-treated samples. In adipocytes, P=0.048 (diabetic versus nondiabetic), two-tailed unpaired Student t test. n = 5 biologically independent samples per

group (**a**). In myocytes, P=0.047 (diabetic versus nondiabetic), two-tailed unpaired Student t test. *n* = 6 (nondiabetic) or 5 (diabetic) biologically independent samples per group (**b**). **c, d** The left panels show the results of western blotting of extracts of proteins from nondiabetic human adipocytes (**c**) and myocytes (**d**) treated with PBS, insulin, tumor necrosis factor (TNF) to induce insulin resistance, and lamivudine (Lam). Immunoreactive bands corresponding to phosphorylated AKT (P-AKT) and total AKT (T-AKT) are shown. The right panels in Panels **c** and **d** show bar graphs of the densitometric analyses of the ratio of P-AKT to T-AKT abundance in these western blots in the various samples compared to that in the PBS-treated samples. Ins, insulin. In nondiabetic adipocytes, P=0.009 (Insulin versus PBS), P=0.10 (Insulin+TNF versus PBS), and P=0.03 (Insulin+TNF+Lam versus PBS), two-tailed unpaired Student t test. In nondiabetic myocytes, P=0.01 (Insulin versus PBS), P=0.10 (Insulin+TNF versus PBS), and P=0.02 (Insulin+TNF+Lam versus PBS), two-tailed unpaired Student t test. **e, f** The left panels show the results of western blotting of extracts of proteins from diabetic human adipocytes (**e**) and myocytes (**f**) treated with PBS, insulin, and Lam. Immunoreactive bands corresponding to phosphorylated AKT (P-AKT) and total AKT (T-AKT) are shown. Data are reported as mean ± s.e.m. In diabetic adipocytes, P=0.14 (Insulin versus PBS) and P=0.02 (Insulin+Lam versus PBS), two-tailed unpaired Student t test. In diabetic myocytes, P=0.08 (Insulin versus PBS) and P=0.01 (Insulin+Lam versus PBS), two-tailed unpaired Student t test. *n* = 5 biologically independent samples per group (**c**–**f**). Source data are provided as a Source Data file.

Supplementary Fig. 13. AKT phosphorylation in human adipocytes treated with AZT or D4T.

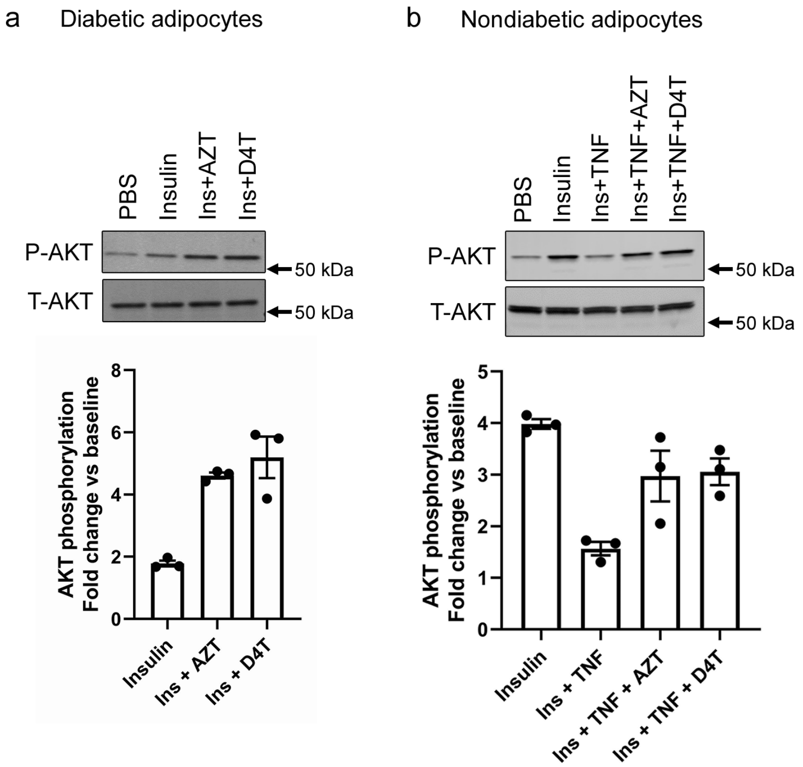

a The top panel shows the results of western blotting of extracts of proteins from diabetic human adipocytes treated with phosphate-buffered saline (PBS), insulin (20 nM; Ins), and AZT (azidothymidine) or D4T (stavudine). Immunoreactive bands corresponding to phosphorylated AKT (P-AKT) and total AKT (T-AKT) are shown. P<0.001 (Ins+AZT versus Insulin), P=0.007 (Ins+D4T versus Insulin), two-tailed unpaired Student t test. *n* = 3 biologically independent samples per group. **b** The top panel shows the results of western blotting of extracts of proteins from nondiabetic human adipocytes treated with PBS, insulin, tumor necrosis factor (TNF) to induce insulin resistance, and AZT or D4T. Immunoreactive bands corresponding to phosphorylated AKT (P-AKT) and total AKT (T-AKT) are shown. P<0.001 (Ins+TNF versus Insulin), P=0.049 (Ins+TNF+AZT versus Ins+TNF), P=0.007 (Ins+TNF+D4T versus Ins+TNF), two-tailed unpaired Student t test. *n* = 3 biologically independent samples per group. The bottom panels in (**a**) and (**b**) show bar graphs of the densitometric analyses of the ratio of P-AKT to T-AKT abundance in these western blots in the various samples compared to that in the PBS-treated samples. Data are reported as mean ± s.e.m. Source data are provided as a Source Data file.

Supplementary Fig. 14. Cell viability of NRTI-treated cells.

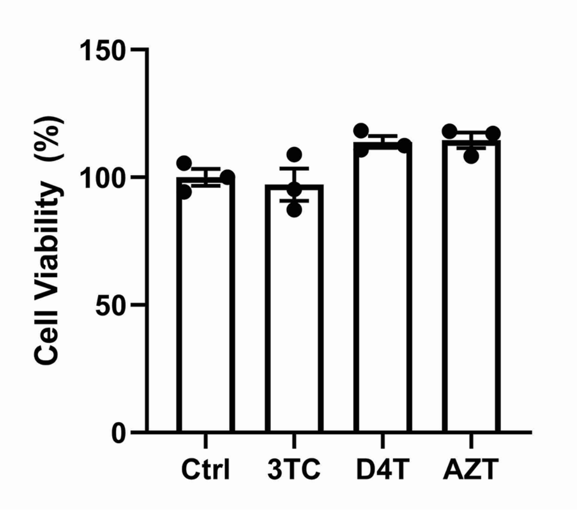

Cell viability of human adipocytes treated with 100 μM NRTIs (lamivudine, 3TC; stavudine, D4T; azidothymidine, AZT) or phosphate-buffered saline (PBS; Ctrl). Values normalized to Ctrl-treated cells. Data are reported as mean ± s.e.m. No reduction in cell viability induced by NRTIs. P=0.64 (3TC vs Ctrl), P=0.99 (D4T vs Ctrl), P=0.99 (AZT vs Ctrl), one-tailed unpaired Student t test). *n* = 3 biologically independent samples per group. Source data are provided as a Source Data file.

Supplementary Fig 15. AKT phosphorylation in high-fat diet-fed mice.

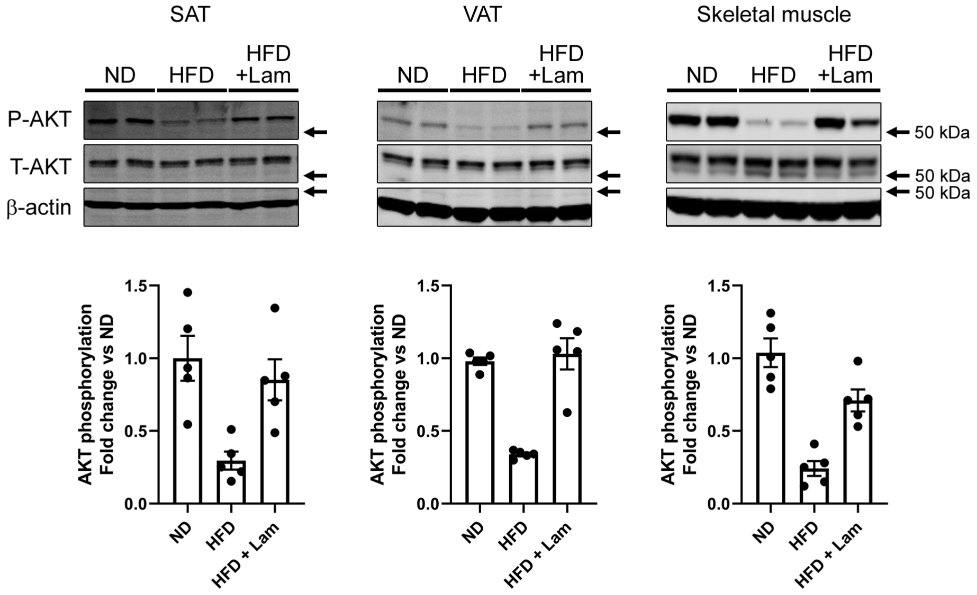

The top three panels show the results of western blotting of extracts of proteins from subcutaneous adipose tissue (SAT), visceral adipose tissue (VAT), and skeletal muscle tissue isolated from mice fed a normal diet (ND), high-fat diet and treated with intraperitoneal injection of phosphate-buffered saline (HFD), and those fed HFD and treated with once-daily intraperitoneal injection of Lamivudine (70 mg/kg of body weight) (HFD+Lam). Immunoreactive bands corresponding to phosphorylated AKT (P-AKT) and beta-actin (β-actin) are shown. The bottom three panels show bar graphs of the densitometric analyses of the P-AKT western blots in the top panels that have been normalized to β-actin abundance and to the ND groups. Data are reported as mean ± s.e.m. In SAT, P=0.003 (ND versus HFD) and P=0.007 (HFD+Lam versus HFD), two-tailed unpaired Student t test. In VAT, P<0.001 (ND versus HFD) and P<0.001 (HFD+Lam versus HFD), two-tailed unpaired Student t test. In Skeletal muscle, P<0.001 (ND versus HFD) and P<0.001 (HFD+Lam versus HFD), two-tailed unpaired Student t test. *n* = 5 biologically independent samples per group. Source data are provided as a Source Data file.

Supplementary Fig. 16. IL-1β and IL-18 expression in high-fat diet-fed mice.

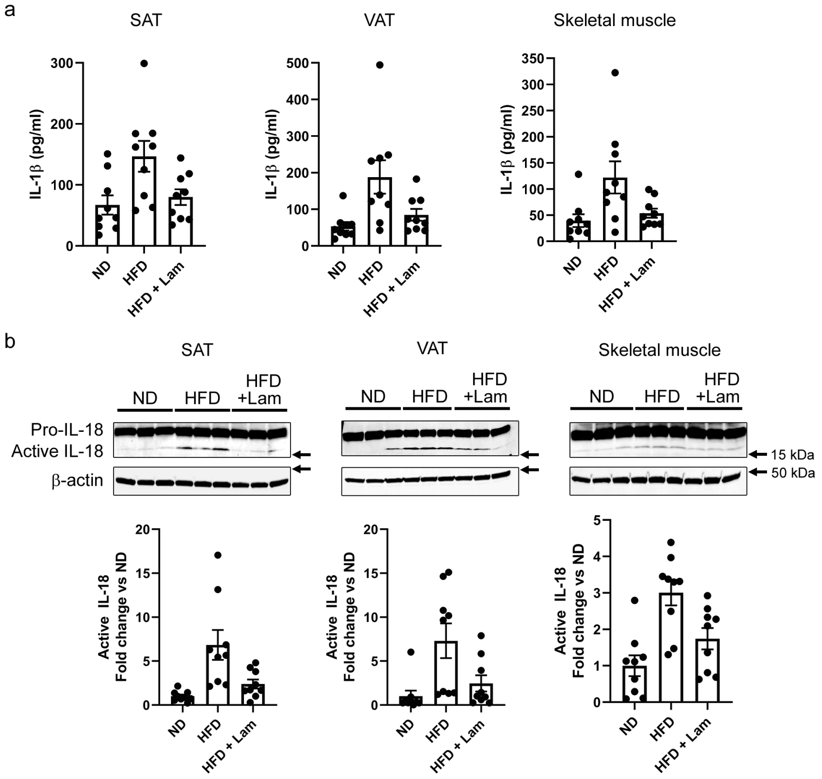

a Bar graphs show the results of ELISA measurements (in pg/ml) of interleukin-1β (IL-1β) of extracts of proteins from subcutaneous adipose tissue (SAT), visceral adipose tissue (VAT), and skeletal muscle tissue isolated from mice fed a normal diet (ND), high-fat diet and treated with intraperitoneal injection of phosphate-buffered saline (HFD), and those fed HFD and treated with intraperitoneal injection of Lamivudine (70 mg/kg of body weight per day) (HFD+Lam). In SAT, P=0.02 (ND versus HFD) and P=0.03 (HFD+Lam versus HFD), two-tailed unpaired Student t test. In VAT, P=0.01 (ND versus HFD) and P=0.049 (HFD+Lam versus HFD), two-tailed unpaired Student t test. In Skeletal muscle, P=0.02 (ND versus HFD) and P=0.048 (HFD+Lam versus HFD), two-tailed unpaired Student t test. **b** The top three panels show the results of western blotting of extracts of proteins from SAT, VAT, and skeletal muscle tissue isolated from ND, HFD, and HFD-Lam groups of mice. Immunoreactive bands corresponding to pro-interleukin-18 (Pro-IL-18), Active IL-18, and beta-actin (β-actin) are shown. The bottom three panels show bar graphs of the densitometric analyses of the Active IL-18 western blots in the top panels that have been normalized to β-actin abundance and to the ND groups. Data are reported as mean ± s.e.m. In SAT, P=0.004 (ND versus HFD) and P=0.02 (HFD+Lam versus HFD), two-tailed unpaired Student t test. In VAT, P=0.008 (ND versus HFD) and P=0.04 (HFD+Lam versus HFD), two-tailed unpaired Student t test. In Skeletal muscle, P<0.001 (ND versus HFD) and P=0.01 (HFD+Lam versus HFD), two-tailed unpaired Student t test. n = 9 biologically independent samples per group. Source data are provided as a Source Data file.

Supplementary Fig. 17. Body weight in high-fat diet-fed mice.

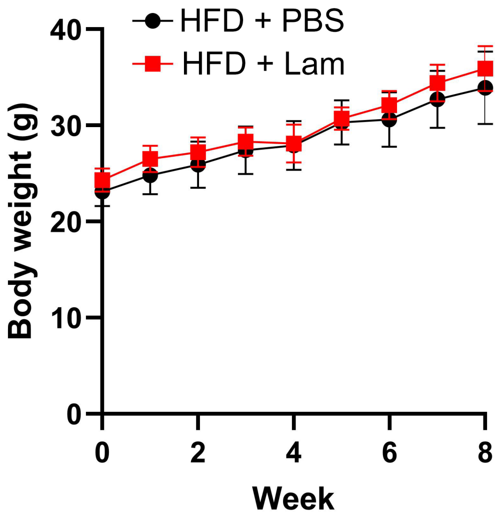

Body weight of mice fed a high fat diet (HFD) and treated with once-daily intraperitoneal injections of phosphate-buffered saline (PBS) or lamivudine (Lam; 70 mg/kg of body weight). Data are reported as mean ± s.d. *n* = 9 animals per group (HFD treated with PBS). *n* = 10 animals per group (HFD treated with Lam). Source data are provided as a Source Data file.

Supplementary Table 1. List of Medications Studied*.

Nucleoside reverse-transcriptase inhibitors (NRTIs)

Abacavir
Adefovir
Didanosine
Emtricitabine
Entecavir
Lamivudine
Stavudine
Tenofovir
Zidovudine

Nonnucleoside reverse-transcriptase inhibitors (NNRTIs)

Delavirdine
Efavirenz
Etravirine
Nevirapine
Rilpivirine

Protease inhibitors (PIs)

Atazanavir
Darunavir
Fosamprenavir
Lopinavir
Ritonavir
Saquinavir
Tipranavir

Integrase strand transfer inhibitors (INSTIs)

Dolutegravir
Elvitegravir
Raltegravir

***Combination drugs (e.g. Cobicistat/Elvitegravir/Emtricitabine/Tenofovir, Emtricitabine/Rilpivirine/Tenofovir) are counted in each component drug's category.**

Supplementary Table 2. Comorbidities, laboratory values, and medications associated with development of diabetes.

Comorbidities	ICD-9-CM	ICD-9-CM
Systemic Hypertension	401.xx – 405.xx	I10 – I15
Depression	296.2, 296.3, 311, 311.0	F32, F33
Pure Hypercholesterolemia	272.0	E78.00, E78.01
Hyperglyceridemia	272.1, 272.2, 272.3, 272.4	E78.1, E78.2, E78.3
Ischemic heart disease	410.xx, 411.0, 411.1, 413.1, 414	I20 – I25
Other heart disease	420.xx – 429.xx	I30 – I52
Stroke	430.xx, 431.xx, 432.xx, 433.x1,433.9, 434.x1, 434.9	I60 – I63, I69
Polycystic ovarian syndrome	256.4	E28.2
Gestational diabetes	648.00, 648.01, 648.02, 648.03, 648.04	O24.4
Acanthosis nigricans	701.2	L83
Hepatitis C	70.54	B18.2
Osteoarthritis	715.xx	M15 – M19
Rheumatoid arthritis	714	M05, M06
Psoriatic arthritis	696.0	L40.50 – L40.54
Family history of diabetes	V18.0	Z83.3

ICD-9-CM/ICD-10-CM, International Classification of Diseases, 9th/10th Revision, Clinical Modification.

Laboratory Tests	LOINC
High-density lipoproteins	2085-9
Triglycerides	2571-8
Alanine aminotransferases	1742-6
Aspartate aminotransferases	1920-8
Bilirubin	1975-2
Amylase	1798-8
Lactate	2524-7

LOINC, Logical Observation Identifiers Names and Codes.

Medications	
Antihypertensives	Acebutolol, Alfuzosin, Aliskiren, Amlodipine, Atenolol, Azilsartan, Benazepril, Bisoprolol, Bumetanide, Candesartan, Captopril, Carvedilol, Chlorothiazide, Chlorthalidone, Clevidipine, Clonidine, Diltiazem, Dobutamine, Doxazosin, Enalapril,

	Eplerone, Esmolol, Ethacrynic acid, Felodipine, Fosinopril, Furosemide, Guanabenz, Guanfacine, Hydralazine, Hydrochlorothiazide, Indapamide, Irbesartan, Isradipine, Labetalol, Lisinopril, Losartan, Methyldopa, Metoprolol, Midodrine, Minoxidil, Moexipril, Nadolol, Nebivolol, Nicardipine, Nifedipine, Nisoldipine, Olmesartan, Perindopril, Pindolol, Prazosin, Propranolol, Quinapril, Ramipril, Spironolactone, Telmisartan, Terazosin, Timolol, Trandolapril, Valsartan, Verapamil
Lipid-lowering medications	Alirocumab, Atorvastatin, Cholestyramine, Colesevelam, Colestipol, Evolocumab, Ezetimibe, Fenofibrate, Fenofibric acid, Gemfibrozil, Lovastatin, Niacin, Pioglitazone, Pitavastatin, Pravastatin, Rosuvastatin, Simvastatin
Fluoroquinolones	Ciprofloxacin, Levofloxacin, Moxifloxacin, Norfloxacin, Ofloxacin
Antipsychotic medications	Amitriptyline, Aripiprazole, Asenapine, Brexpiprazole, Chlorpromazine, Clozapine, Fluoxetine, Fluphenazine, Haloperidol, Loxapine, Lurasidone, Olanzapine, Paliperidone, Perphenazine, Pimozide, Prochlorperazine, Quetiapine, Risperidone, Thiothixene, Trifluoperazine, Ziprasidone
Corticosteroids	Betamethasone, Dexamethasone, Hydrocortisone, Methylprednisolone, Prednisone, Prednisolone, Triamcinolone

Codes for generic and brand name versions of medications available from authors upon request.

Supplementary Table 3. Characteristics of the Patients.

Variable	Value	Veterans (N=79,744) %	Truven (N=23,634) %	PearlDiver (N=16,045) %	Medicare (N=3,097) %	Clinformatics (N=6,341) %
**Incident diabetes**	**No**	84.6	93.1	93.3	77.2	83.2
	**Yes**	15.4	6.9	6.7	22.8	16.8
**NRTI Use**	**Never**	46.7	52.1	39.6	60.1	60.6
	**Ever**	53.3	47.9	60.4	39.9	39.4
**NNRTI Use**	**Never**	77.9	77.7	70.5	82.4	80.1
	**Ever**	22.1	22.3	29.5	17.6	19.9
**PI Use**	**Never**	79.5	84.1	73.3	83.5	82.8
	**Ever**	20.5	15.9	26.7	16.5	17.2
**INSTI Use**	**Never**	78.0	88.7	73.9	88.2	89.5
	**Ever**	22.0	11.3	26.1	11.8	10.5
**Sex**	**Male**	94.1	66.2	67.6	54.7	63.4
	**Female**	5.9	33.8	32.4	45.3	36.6
**Race**	**White**	45.7	N/A	37.8	39.2	48.2
	**Black**	31.8	N/A	19.4	17.4	15.5
	**Asian**	1.8	N/A	2.1	30.3	21.1
	**Latino/Hispanic**	0.3	N/A	1.8	2.8	7.7
	**Unknown/Other**	12.3	N/A	39.1	10.2	7.5
**Variable**		**Mean (SD)**	**Mean (SD)**	**Mean (SD)**	**Mean (SD)**	**Mean (SD)**
**Age at Index Date**		48.6 (11.3)	58.2 (2.58)	53.8 (14.2)	73.4 (5.5)	66.7 (6.8)
**Year of Index Date**		2004.7 (4.9)	2010.3 (2.7)	2011.3 (2.8)	2012.1 (1.7)	2010.7 (4.0)
**Charlson comorbidity index**		6.3 (4.0)	4.5 (3.54)	4.5 (3.0)	6.8 (3.3)	6.1 (3.7)

NRTI denotes nucleoside reverse-transcriptase inhibitor, NNRTI non-nucleoside reverse-transcriptase inhibitor, PI protease inhibitor, INSTI integrase strand transfer inhibitor, N/A not available, and SD standard deviation.

Supplementary Table 4. Baseline characteristics of Veterans database.

Variable		No record of NRTI N=37,227	NRTI exposure N=42,517	P value*
**Race**	**Black**	10,493 (28.2%)	14,870 (34.5%)	<0.001
	**Other**	11,015 (29.6%)	13,499 (31.8%)	<0.001
	**White**	15,719 (42.2%)	14,148 (33.3%)	<0.001
**Charlson comorbidity index Mean (SD)**		3.99 (3.45)	8.29 (3.26)	<0.001
**Lipid lowering medication use**		13,881 (37.3%)	15,466 (36.4%)	0.008
**Antihypertensive medication use**		22,508 (60.5%)	23,393 (55.0%)	<0.001
**Tobacco use**		18,397 (49.4%)	17,282 (40.7%)	<0.001
**Index age – Mean (SD)**		50.09 (11.50)	47.33 (11.03)	<0.001
**Index year – Mean (SD)**		2004.75 (4.38)	2004.67 (5.39)	0.025
**Viral load**	**>1000**	1,520 (4.1%)	18,708 (44.0%)	<0.001
	**0-500**	1,389 (3.7%)	14,446 (34.0%)	<0.001
	**501-1000**	110 (0.3%)	1,338 (3.2%)	<0.001
	**Missing**	34,208 (91.9%)	8,025 (18.9%)	<0.001
**CD4 counts**	**>250**	1,139 (3.1%)	12,875 (30.3%)	<0.001
	**0-50**	195 (0.5%)	1,834 (4.3%)	<0.001
	**51-250**	263 (0.7%)	4,581 (10.8%)	<0.001
	**Missing**	35,630 (95.7%)	23,226 (54.6%)	<0.001
**BMI**	**Missing**	5,399 (14.5%)	7,761 (18.3%)	<0.001
	**Normal**	10,462 (28.1%)	15,655 (36.8%)	<0.001
	**Obese**	8,502 (22.8%)	5,795 (13.6%)	<0.001
	**Overweight**	12,133 (32.6%)	12,041 (28.3%)	<0.001
	**Underweight**	731 (2.0%)	1,265 (3.0%)	<0.001
**Systemic hypertension**		24,113 (64.8%)	24,727 (58.2%)	<0.001
**Depression**		20,405 (54.8%)	21,452 (50.5%)	<0.001
**Ischemic heart disease**		8,982 (24.1%)	8,325 (19.6%)	<0.001
**Other heart disease**		12,020 (32.3%)	12,224 (28.8%)	<0.001
**Stroke**		2,763 (7.4%)	2,524 (6.0%)	<0.001
**Hepatitis C**		11,080 (29.8%)	10,010 (23.5%)	<0.001
**Osteoarthritis**		16,622 (44.7%)	12,363 (29.1%)	<0.001
**Rheumatoid arthritis**		1,067 (2.9%)	774 (1.8%)	<0.001
**HDL**	**<40**	11,927 (32.0%)	19,081 (44.9%)	<0.001
	**>=60**	6,274 (16.9%)	4,908 (11.5%)	<0.001
	**40-59**	15,601 (41.9%)	15,330 (36.1%)	<0.001
	**Missing**	3,425 (9.2%)	3,198 (7.5%)	<0.001
**Triglycerides**	**<150**	23,515 (63.2%)	22,906 (53.9%)	<0.001
	**>=500**	535 (1.4%)	1,449 (3.4%)	<0.001

	**150-199**	4,803 (12.9%)	6,273 (14.8%)	<0.001
	**200-499**	5,376 (14.4%)	9,252 (21.8%)	<0.001
	**Missing**	2,998 (8.1%)	2,637 (6.2%)	<0.001
**ALT**	**<40**	21,033 (56.5%)	25,368 (59.7%)	<0.001
	**>120**	2,316 (6.2%)	2,195 (5.2%)	<0.001
	**40-79**	8,696 (23.4%)	10,155 (23.9%)	<0.001
	**80-120**	2,126 (5.7%)	2,488 (5.9%)	<0.001
	**Missing**	3,056 (8.2%)	2,311 (5.4%)	<0.001
**AST**	**<40**	24,904 (66.9%)	27,829 (65.5%)	<0.001
	**>120**	2,338 (6.3%)	2,232 (5.3%)	<0.001
	**40-79**	6,864 (18.4%)	9,722 (22.9%)	<0.001
	**80-120**	1,970 (5.3%)	2,307 (5.4%)	<0.001
	**Missing**	1,151 (3.1%)	427 (1.0%)	<0.001
**Bilirubin**	**<2**	34,391 (92.4%)	40,421 (95.1%)	<0.001
	**>3**	808 (2.2%)	632 (1.5%)	<0.001
	**2--3**	739 (2.0%)	958 (2.3%)	<0.001
	**Missing**	1,289 (3.5%)	506 (1.2%)	<0.001
**Lactate**	**<2**	3,894 (10.5%)	8,039 (18.9%)	<0.001
	**>4**	409 (1.1%)	511 (1.2%)	<0.001
	**3--4**	1,293 (3.5%)	2,290 (5.4%)	<0.001
	**Missing**	31,631 (85.0%)	31,677 (74.5%)	<0.001
**Antipsychotic medication use**		4,528 (12.2%)	3,121 (7.3%)	<0.001
**Corticosteroid use**		2,422 (6.6%)	2,311 (5.5%)	<0.001

*P-values for continuous variables are from unpaired Student t tests and categorical from chi-square (χ^2) tests. All statistical tests are two-sided and not adjusted for multiple comparisons.

Supplementary Table 5. Hazard of Incident Diabetes for NRTI use (ever/never) in the Veterans Health Administration Database.

Variable		Adjusted HR (95% CI)
**NRTI use ever**		0.665 (0.625-0.708)
**NNRTI use ever**		1.115 (1.052-1.183)
**PI use ever**		1.281 (1.206-1.362)
**INSTI use ever**		0.443 (0.418-0.468)
**Sex: Female**		0.947 (0.862-1.041)
**Race (baseline=White)**	**Black**	1.410 (1.350-1.473)
	**Other**	1.198 (1.136-1.264)
**Charlson comorbidity index**		1.090 (1.084-1.097)
**Lipid lowering medication use**		1.749 (1.669-1.833)
**Antihypertensive medication use**		1.212 (1.154-1.273)
**Tobacco use**		0.978 (0.941-1.017)
**Index age**		1.029 (1.027-1.031)
**Index year**		1.225 (1.217-1.232)
**Viral load**	**0-500**	0.891 (0.828-0.958)
	**501-100**	0.776 (0.666-0.903)
	**>1000**	0.847 (0.783-0.917)
**CD4 counts**	**0-50**	0.991 (0.855-1.149)
	**51-250**	0.916 (0.838-1.002)
	**>250**	0.865 (0.816-0.916)
**BMI (baseline Underweight)**	**Missing**	1.021 (0.858-1.213)
	**Normal**	0.975 (0.823-1.155)
	**Obese**	2.185 (1.845-2.589)
	**Overweight**	1.344 (1.135-1.590)
**Systemic hypertension**		1.263 (1.198-1.332)
**Pure hypercholesterolemia**		1.151 (0.965-1.374)
**Hyperglyceridemia**		0.737 (0.617-0.880)
**Depression**		0.829 (0.798-0.862)
**Ischemic heart disease**		0.916 (0.877-0.957)
**Other heart disease**		0.850 (0.815-0.886)
**Stroke**		0.719 (0.672-0.769)
**Polycystic ovarian syndrome**		1.522 (1.074-2.156)
**Gestational diabetes**		1.487 (0.798-2.773)
**Acanthosis nigricans**		0.933 (0.886-0.982)
**Hepatitis C**		0.866 (0.828-0.907)
**Osteoarthritis**		0.798 (0.768-0.829)

**Rheumatoid arthritis**		1.034 (0.930-1.150)
**Psoriatic arthritis**		0.859 (0.781-0.945)
**HDL (Baseline=Missing)**	**40-59**	0.817 (0.707-0.944)
	**<40**	0.989 (0.856-1.142)
	**>=60**	0.717 (0.616-0.836)
**Triglycerides (baseline=Missing)**	**150-199**	1.023 (0.848-1.234)
	**200-499**	1.218 (1.011-1.468)
	**<150**	0.888 (0.739-1.067)
	**>=500**	1.454 (1.193-1.771)
**ALT (Baseline=Missing)**	**40-79**	1.000 (0.909-1.101)
	**80-120**	1.011 (0.899-1.137)
	**<40**	0.833 (0.759-0.913)
	**>120**	0.920 (0.806-1.050)
**AST (Baseline=Missing)**	**40-79**	2.098 (1.401-3.143)
	**80-120**	2.284 (1.514-3.447)
	**<40**	1.890 (1.265-2.826)
	**>120**	2.273 (1.500-3.445)
**Bilirubin (Baseline=Missing)**	**2--3**	1.524 (1.039-2.236)
	**<2**	1.417 (0.987-2.035)
	**>3**	1.418 (0.961-2.093)
**Amylase (Baseline=Missing)**	**<=140**	1.197 (1.149-1.247)
	**>140**	1.225 (1.137-1.321)
**Lactate (Baseline=Missing)**	**2--4**	1.480 (1.375-1.592)
	**<2**	1.188 (1.131-1.248)
	**>4**	1.739 (1.497-2.021)
**Family history of diabetes**		1.406 (1.232-1.604)
**Fluoroquinolone use**		1.075 (1.007-1.147)
**Antipsychotic medication use**		1.147 (1.082-1.216)
**Corticosteroid use**		0.944 (0.876-1.018)

NRTI denotes nucleoside reverse-transcriptase inhibitor, NNRTI non-nucleoside reverse-transcriptase inhibitor, PI protease inhibitor, INSTI integrase strand transfer inhibitor, BMI body-mass index (mass in kilograms divided by the square of the height in meters), HDL high-density lipoprotein, ALT alanine aminotransferase, AST aspartate aminotransferase, HR hazard ratio, and CI confidence interval. Viral Load is measured in RNA copies per milliliter and CD4 count in cells per cubic millimeter. HDL, Triglycerides, and Bilirubin are measured in milligrams per deciliter. AST, ALT, and Amylase are measured in units per liter. Hazard ratios based on a Cox proportional-hazards model and adjusted for the confounding variables are presented.

Supplementary Table 6. Baseline characteristics of Truven Marketscan database.

Variable		No record of NRTI N=12,308	NRTI Exposure N=11,326	P value*
**Index age – Mean (SD)**		58.53 (2.61)	57.99 (2.52)	<0.001
**Sex**	**Male**	6,496 (52.8%)	9,140 (80.7%)	<0.001
	**Female**	5,812 (47.2%)	2,186 (19.3%)	<0.001
**Charlson comorbidity index Mean (SD)**		2.52 (2.59)	6.66 (3.16)	<0.001
**Index year – Mean (SD)**		2010.5 (2.69)	2010.09 (2.79)	<0.001
**Depression**		2,226 (18.1%)	2,007 (17.7%)	0.47
**Systemic hypertension**		7,236 (58.8%)	6,129 (54.1%)	<0.001
**Hyperglyceridemia**		7,902 (64.2%)	7,004 (61.8%)	<0.001
**Stroke**		1,030 (8.4%)	721 (6.4%)	<0.001
**Polycystic ovarian syndrome**		184 (1.5%)	40 (0.4%)	<0.001
**Gestational diabetes**		5 (0.04%)	2 (0.02%)	0.31
**Acanthosis nigricans**		1,068 (8.7%)	639 (5.6%)	<0.001
**Hepatitis C**		2,373 (19.3%)	1,063 (9.4%)	<0.001
**Osteoarthritis**		4,123 (33.5%)	2,088 (18.4%)	<0.001
**Rheumatoid arthritis**		816 (6.6%)	245 (2.2%)	<0.001
**Psoriatic arthritis**		441 (3.6%)	265 (2.3%)	<0.001
**Family history of diabetes**		136 (1.1%)	62 (0.6%)	<0.001
**Fluoroquinolone use**		2,832 (23.0%)	976 (8.6%)	<0.001
**Corticosteroid use**		2,730 (22.2%)	873 (7.7%)	<0.001
**Antihypertensive medication use**		8,048 (65.4%)	5,047 (44.6%)	<0.001
**Lipid lowering medication use**		1,808 (14.7%)	1,017 (9.0%)	<0.001

*P-values for continuous variables are from unpaired Student t tests and categorical from chi-square (χ^2) tests. All statistical tests are two-sided and not adjusted for multiple comparisons.

Supplementary Table 7. Hazard of Incident Diabetes for NRTI use (ever/never) in the Truven Marketscan Database.

Variable	Adjusted HR (95% CI)
**NRTI use ever**	0.614 (0.524-0.718)
**NNRTI use ever**	0.638 (0.547-0.745)
**PI use ever**	0.753 (0.641-0.884)
**INSTI use ever**	0.446 (0.364-0.547)
**Index Age**	0.954 (0.927-0.982)
**Sex: Male**	1.081 (0.964-1.213)
**Charlson comorbidity index**	1.197 (1.182-1.212)
**Index year**	0.893 (0.876-0.910)
**Depression**	0.949 (0.836-1.079)
**Systemic hypertension**	1.248 (1.115-1.397)
**Hyperglyceridemia**	1.163 (1.033-1.310)
**Stroke**	0.835 (0.707-0.986)
**Polycystic ovarian syndrome**	0.916 (0.520-1.614)
**Gestational diabetes**	1.784 (0.353-9.019)
**Acanthosis nigricans**	0.622 (0.502-0.770)
**Hepatitis C**	0.749 (0.648-0.866)
**Osteoarthritis**	0.809 (0.720-0.909)
**Rheumatoid arthritis**	1.113 (0.883-1.404)
**Psoriatic arthritis**	0.766 (0.565-1.038)
**Family History of Diabetes**	0.867 (0.471-1.594)
**Fluoroquinolone use**	1.030 (0.884-1.199)
**Corticosteroid use**	0.867 (0.731-1.027)
**Antihypertensive medication use**	1.089 (0.973-1.219)
**Lipid lowering medication use**	1.330 (1.151-1.537)

NRTI denotes nucleoside reverse-transcriptase inhibitor, NNRTI non-nucleoside reverse-transcriptase inhibitor, PI protease inhibitor, INSTI integrase strand transfer inhibitor. Hazard ratios based on a Cox proportional-hazards model and adjusted for the confounding variables are presented.

Supplementary Table 8. Baseline characteristics of PearlDiver database.

Variable		No record of NRTI N=6,360	NRTI exposure N=9,685	P value*
**Sex**	**Male**	3,541 (55.7%)	7,311 (75.5%)	<0.001
	**Female**	2,819 (44.3%)	2,374 (24.5%)	<0.001
**Race**	**Black**	980 (10.1%)	2,127 (22.0%)	<0.001
	**White**	2,904 (30.0%)	3,154 (32.6%)	<0.001
**Charlson comorbidity index Mean (SD)**		4.01 (3.75)	6.08 (2.48)	<0.001
**Tobacco use**		1,876 (29.5%)	2,798 (28.9%)	0.41
**Index age - Mean (SD)**		58.0 (16.9)	51.1 (12.0)	<0.001
**Index year - Mean (SD)**		2011.4 (2.6)	2011.3 (2.9)	0.03
**Viral load**	**>1000**	6 (0.1%)	51 (0.5%)	<0.001
	**0-500**	287 (4.5%)	3,179 (32.8%)	<0.001
	**501-1000**	14 (0.2%)	179 (1.9%)	<0.001
**CD4 counts**	**>250**	194 (3.1%)	2,164 (22.3%)	<0.001
	**0-50**	42 (0.7%)	525 (5.4%)	<0.001
	**51-250**	58 (0.9%)	944 (9.8%)	<0.001
**BMI**	**30-40**	1,154 (18.1%)	1,205 (12.4%)	<0.001
	**>40**	559 (8.8%)	490 (5.1%)	<0.001
**Systemic hypertension**		4,734 (74.4%)	5,707 (58.9%)	<0.001
**Pure hypercholesterolemia**		2,883 (45.3%)	3,734 (38.6%)	<0.001
**Hyperglyceridemia**		4,571 (71.9%)	6,183 (63.8%)	<0.001
**Depression**		1,898 (29.8%)	2,765 (28.6%)	0.10
**Ischemic heart disease**		1,136 (17.9)%	912 (9.4%)	<0.001
**Other heart disease**		3,041 (47.8%)	2,795 (28.9%)	<0.001
**Stroke**		645 (10.1%)	505(5.2%)	<0.001
**Polycystic ovarian syndrome**		22 (0.4%)	15 (0.2%)	0.01
**Acanthosis nigricans**		13 (0.2%)	17 (0.2%)	0.68
**Hepatitis C**		405 (6.4%)	350 (3.6%)	<0.001
**Osteoarthritis**		2,828 (44.5%)	2,395 (24.7%)	<0.001
**Rheumatoid arthritis**		561 (8.8%)	275 (2.8%)	<0.001
**Psoriatic arthritis**		44 (0.7%)	35 (0.4%)	0.003
**HDL**	**40-59**	3,305 (52.0%)	5,044 (52.1%)	<0.001
	**<40**	1,967 (30.9%)	3,932 (40.6%)	<0.001
	**>=60**	1,968 (30.9%)	2,385 (24.6%)	<0.001
**Triglycerides**	**150-199**	1,891 (29.7%)	3,329 (34.4%)	<0.001
	**200-499**	1,436 (22.6%)	3,007 (31.1%)	<0.001
	**<150**	4,121 (64.8%)	5,898 (60.9%)	<0.001
	**>=500**	152 (2.4%)	404 (4.2%)	<0.001

**ALT**	**<40**	1,788 (28.1%)	3,354 (34.6%)	<0.001
	**>120**	638 (10.0%)	1,082 (11.2%)	0.02
	**40-79**	4,972 (78.2%)	7,768 (80.2%)	0.002
	**80-120**	504 (7.9%)	733 (7.6%)	0.41
**AST**	**40-79**	1,612 (25.4%)	2,780 (28.7%)	<0.001
	**80-120**	590 (9.3%)	842 (8.7%)	0.20
	**<40**	4,980 (78.3%)	7,807 (80.6%)	<0.001
	**>120**	502 (7.9%)	672 (6.9%)	<0.001
**Bilirubin**	**2-3**	286 (4.5%)	786 (8.1%)	<0.001
	**<2**	5,211 (81.9%)	8,005 (82.7%)	0.19
	**>3**	208 (3.3%)	519 (5.4%)	<0.001
**Amylase**	**<=140**	655 (10.3%)	1,142 (11.8%)	0.003
	**>140**	66 (1.0%)	167 (1.7%)	<0.001
**Family history of diabetes**		173 (2.7%)	212 (2.2%)	0.03
**Fluoroquinolone use**		3,094 (48.7%)	4,522 (46.7%)	0.01
**Antipsychotic medication use**		927 (14.6%)	1,614 (16.7%)	<0.001
**Corticosteroid use**		3,625 (57.0%)	5,309 (54.8%)	0.006
**Lipid lowering medication use**		2,778 (43.7%)	4,056 (41.9%)	0.02
**Antihypertensive medication use**		4,084 (64.2%)	5,456 (56.3%)	<0.001

*P-values for continuous variables are from unpaired Student t tests and categorical from chi-square (χ^2) tests. All statistical tests are two-sided and not adjusted for multiple comparisons.

Supplementary Table 9. Hazard of Incident Diabetes for NRTI use (ever/never) in the PearlDiver Database.

Variable		Adjusted HR(95% CI)
**NRTI use ever**		0.738 (0.600-0.908)
**NNRTI use ever**		0.701 (0.567-0.867)
**PI use ever**		0.757 (0.605-0.948)
**INSTI use ever**		0.669 (0.542-0.827)
**Sex (reference=Female)**	**Male**	0.983 (0.841-1.149)
**Race (reference=White)**	**Black**	1.268 (1.027-1.508)
	**Hispanic**	1.217 (0.896-1.538)
**Charlson comorbidity index**		1.092 (1.071-1.115)
**Tobacco use**		0.726 (0.620-0.851)
**Age (Per year)**		0.989 (0.968-1.010)
**Index year (Per year)**		1.023 (0.963-1.087)
**Viral Load (reference=missing)**	**0-500**	0.991 (0.795-1.236)
	**501-1000**	0.684 (0.301-1.557)
	**>1000**	0.601 (0.083-4.336)
**CD4 count (reference=missing)**	**0-50**	0.803 (0.514-1.256)
	**51-250**	1.332 (0.971-1.829)
	**>250**	1.081 (0.853-1.371)
**BMI (reference=normal)**	**30-40**	1.311 (1.115-1.542)
	**>40**	0.726 (0.620-0.851)
**Systemic hypertension**		2.362 (1.741-3.205)
**Pure hypercholesterolemia**		1.125 (0.963-1.315)
**Hyperglyceridemia**		1.949 (1.469-2.586)
**Depression**		1.033 (0.889-1.201)
**Ischemic heart disease**		1.179 (1.006-1.381)
**Other heart disease**		1.260 (1.074-1.478)
**Stroke**		0.890 (0.729-1.089)
**Polycystic ovarian syndrome**		1.747 (0.543-5.642)
**Gestational diabetes**		24.17 (8.246-70.90)
**Acanthosis nigricans**		1.232 (0.391-3.880)
**Hepatitis C**		1.300 (1.029-1.641)
**Osteoarthritis**		1.101 (0.952-1.273)
**Rheumatoid arthritis**		0.871 (0.681-1.114)
**Psoriatic arthritis**		1.244 (0.634-2.442)
**HDL (Baseline=Missing)**	**40-59**	1.033 (0.874-1.220)
	**<40**	1.417 (1.185-1.696)
	**>=60**	0.967 (0.809-1.155)
**Triglycerides (Baseline=Missing)**	**150-199**	0.907 (0.742-1.109)
	**200-499**	0.968 (0.822-1.139)

	**<150**	0.967 (0.809-1.155)
	**>=500**	1.138 (0.892-1.354)
**ALT (Baseline=Missing)**	**40-79**	1.148 (0.952-1.383)
	**80-120**	1.100 (0.858-1.412)
	**<40**	0.976 (0.642-1.483)
	**>120**	1.132 (0.827-1.548)
**AST (Baseline=Missing)**	**40-79**	1.026 (0.846-1.244)
	**80-120**	1.070 (0.820-1.396)
	**<40**	0.985 (0.639-1.519)
	**>120**	0.788 (0.567-1.096)
**Bilirubin (Baseline=Missing)**	**2-3**	0.990 (0.731-1.340)
	**<2**	0.718 (0.483-1.067)
	**>3**	0.988 (0.688-1.318)
**Amylase (Baseline=Missing)**	**<=140**	1.058 (0.873-1.282)
	**>140**	0.820 (0.505-1.333)
**Family history of diabetes**		1.606 (1.171-2.201)
**Fluoroquinolone use**		1.062 (0.919-1.228)
**Antipsychotic medication use**		1.148 (0.963-1.368)
**Corticosteroid Use**		0.827 (0.714-0.958)
**Antihypertensive medication use**		1.416 (1.132-1.771)
**Lipid lowering medication use**		1.462 (1.241-1.721)

NRTI denotes nucleoside reverse-transcriptase inhibitor, NNRTI non-nucleoside reverse-transcriptase inhibitor, PI protease inhibitor, INSTI integrase strand transfer inhibitor, BMI body-mass index (mass in kilograms divided by the square of the height in meters), HDL high-density lipoprotein, ALT alanine aminotransferase, AST aspartate aminotransferase, HR hazard ratio, and CI confidence interval. Viral Load is measured in RNA copies per milliliter and CD4 count in cells per cubic millimeter. HDL, Triglycerides, and Bilirubin are measured in milligrams per deciliter. AST, ALT, and Amylase are measured in units per liter. Hazard ratios based on a Cox proportional-hazards model and adjusted for the confounding variables are presented.

Supplementary Table 10. Baseline characteristics of Medicare database.

Variable	No record of NRTI (N=1,860)	NRTI exposure (N=1,237)	P value*
**Sex: Female**	1,007 (54.1%)	397 (32.1%)	<0.001
**White**	615 (33.1%)	599 (48.4%)	<0.001
**Black**	177 (9.5%)	363 (29.3%)	<0.001
**Asian**	776 (41.7%)	163 (13.2%)	<0.001
**Latino/Hispanic**	55 (3.0%)	33 (2.7%)	0.64
**Charlson comorbidity index (mean/SD)**	5.6 (2.6)	8.7 (3.4)	<0.001
**Index age (mean/SD)**	74.3 (6.0)	71.9 (4.3)	<0.001
**Index year (mean/SD)**	2012.0 (1.6)	2012.3 (1.8)	<0.001
**Systemic hypertension**	1,379 (74.1%)	659 (53.3%)	<0.001
**Pure hypercholesterolemia**	568 (30.5%)	218 (17.6%)	<0.001
**Hypertriglyceridemia**	333 (17.9%)	176 (14.2%)	0.007
**Depression**	56 (3.0%)	54 (4.4%)	0.05
**Ischemic heart disease**	524 (28.2%)	303 (24.5%)	0.02
**Other heart disease**	746 (40.1%)	451 (36.5%)	0.04
**Stroke**	267 (14.4%)	141 (11.4%)	0.02
**Polycystic ovarian syndrome**	1 (0.1%)	0 (0.0%)	0.41
**Gestational diabetes**	0 (0.0%)	0 (0.0%)	
**Acanthosis nigricans**	0 (0.0%)	3 (0.2%)	0.03
**Hepatitis C**	159 (8.5%)	76 (6.1%)	0.01
**Osteoarthritis**	637 (34.2%)	290 (23.4%)	<0.001
**Rheumatoid arthritis**	97 (5.2%)	24 (1.9%)	<0.001
**Psoriatic arthritis**	7 (0.4%)	2 (0.2%)	0.28
**Family history of diabetes**	0 (0.0%)	0 (0.0%)	

*P-values for continuous variables are from unpaired Student t tests and categorical from chi-square (χ^2) tests. All statistical tests are two-sided and not adjusted for multiple comparisons.

Supplementary Table 11. Hazard of Incident Diabetes for NRTI use (ever/never) in the Medicare Database.

Variable		Adjusted HR (95% CI)
**NRTI use ever**		0.828 (0.646-1.062)
**NNRTI use ever**		0.803 (0.594-1.084)
**PI use ever**		0.857 (0.623-1.178)
**INSTI use ever**		0.903 (0.626-1.303)
**Sex (reference=Male)**	**Female**	1.079 (0.922-1.262)
**Race (reference =White)**	**Black**	1.519 (1.190-1.938)
	**Latino/Hispanic**	2.237 (1.518-3.297)
	**Asian**	1.832 (1.495-2.246)
**Charlson comorbidity index**		1.060 (1.027-1.094)
**Index age (Per year)**		0.977 (0.961-0.992)
**Index year (Per year)**		0.896 (0.832-0.964)
**Systemic hypertension**		1.225 (1.022-1.470)
**Pure hypercholesterolemia**		1.240 (1.055-1.459)
**Hypertriglyceridemia**		1.200 (1.000-1.439)
**Depression**		1.283 (0.861-1.912)
**Ischemic heart disease**		1.480 (1.252-1.749)
**Other heart disease**		1.103 (0.935-1.301)
**Stroke**		1.352 (1.104-1.655)
**Polycystic ovarian syndrome**		
**Gestational diabetes**		
**Acanthosis nigricans**		7.288 (1.754-30.28)
**Hepatitis C**		0.885 (0.675-1.161)
**Osteoarthritis**		1.256 (1.072-1.473)
**Rheumatoid arthritis**		0.888 (0.619-1.273)
**Psoriatic arthritis**		
**Family history of diabetes**		

NRTI denotes nucleoside reverse-transcriptase inhibitor, NNRTI non-nucleoside reverse-transcriptase inhibitor, PI protease inhibitor, INSTI integrase strand transfer inhibitor, HR hazard ratio, and CI confidence interval. Hazard ratios based on a Cox proportional-hazards model and adjusted for the confounding variables are presented. Blank cell indicates insufficient evidence to evaluate the regression coefficient and odds ratio.

Supplementary Table 12. Baseline characteristics of Clinformatics database.

Variable	No record of NRTI (N=3,845)	NRTI exposure (N=2,496)	P value*
**Sex: Female**	1,806 (47.0%)	514 (20.6%)	<0.001
**White**	1,727 (44.9%)	1,328 (53.2%)	<0.001
**Black**	487 (12.7%)	498 (20.0%)	<0.001
**Asian**	1,054 (27.4%)	285 (11.4%)	<0.001
**Latino/Hispanic**	265 (6.9%)	224 (9.0%)	0.002
**Charlson (mean/SD)**	4.9 (3.1)	8.0 (3.7)	<0.001
**Index Age (mean/SD)**	67.8 (7.1)	65.1 (6.0)	<0.001
**Index year (mean/SD)**	2010.4 (4.0)	2011.2 (3.8)	<0.001
**Bachelor or more**	630 (16.4%)	514 (20.6%)	<0.001
**Some college**	1,806 (47.0%)	1,112 (44.6%)	0.06
**High school diploma**	1,141 (29.7%)	738 (29.6%)	0.93
**Less than High school**	34 (0.9%)	27 (1.1%)	0.43
**>$500,000**	1,040 (27.0%)	542 (21.7%)	<0.001
**$250,000-$499,000**	862 (22.4%)	515 (20.6%)	0.09
**$150,000-$249,000**	444 (11.5%)	335 (13.4%)	0.02
**$25,000-$149,000**	598 (15.6%)	476 (19.1%)	<0.001
**<$25,000**	332 (8.6%)	268 (10.7%)	0.005
**Urban**	3,565 (92.7%)	2,353 (94.3%)	0.01
**Large Rural**	132 (3.4%)	59 (2.4%)	0.02
**Small Rural**	97 (2.5%)	60 (2.4%)	0.76
**Systemic hypertension**	2,408 (62.6%)	1,414 (56.7%)	<0.001
**Pure hypercholesterolemia**	1,085 (28.2%)	669 (26.8%)	0.22
**Hypertriglyceridemia**	686 (17.8%)	484 (19.4%)	0.12
**Depression**	478 (12.4%)	368 (14.7%)	0.008
**Ischemic heart disease**	492 (12.8%)	261 (10.5%)	0.005
**Other heart disease**	626 (16.3%)	299 (12.0%)	<0.001
**Stroke**	117 (3.0%)	63 (2.5%)	0.22
**Polycystic ovarian syndrome**	0 (0.0%)	0 (0.0%)	
**Gestational diabetes**	0 (0.0%)	0 (0.0%)	
**Acanthosis nigricans**	0 (0.0%)	0 (0.0%)	
**Hepatitis C**	380 (9.9%)	232 (9.3%)	0.44
**Osteoarthritis**	539 (14.0%)	154 (6.2%)	<0.001
**Rheumatoid arthritis**	89 (2.3%)	21 (0.8%)	<0.001
**Psoriatic arthritis**	6 (0.2%)	2 (0.1%)	0.41
**Family history of diabetes**	6 (0.2%)	4 (0.2%)	0.97

*P-values for continuous variables are from unpaired Student t tests and categorical from chi-square (χ^2) tests. All statistical tests are two-sided and not adjusted for multiple comparisons.

Supplementary Table 13. Hazard of Incident Diabetes for NRTI use (ever/never) in the Clinformatics Database.

Variable		Adjusted HR (95% CI)
**NRTI use ever**		0.727 (0.572-0.924)
**NNRTI use ever**		0.894 (0.685-1.167)
**PI use ever**		0.961 (0.732-1.260)
**INSTI use ever**		1.512 (1.093-2.091)
**Sex (reference=Male)**	**Female**	1.018 (0.883-1.173)
**Race (reference =White)**	**Black**	1.484 (1.235-1.783)
	**Latino/Hispanic**	1.405 (1.114-1.771)
	**Asian**	1.235 (1.029-1.484)
**Charlson comorbidity index**		1.003 (0.978-1.029)
**Index age (Per year)**		1.007 (0.997-1.018)
**Index year (Per year)**		0.955 (0.936-0.975)
**Education (reference=Some college)**	**Bachelor or more**	0.848 (0.691-1.042)
	**High school diploma**	1.099 (0.941-1.284)
	**Less than High school**	1.927 (1.094-3.394)
**Household Net Worth (reference=$150 -$249K)**	**>$500,000**	0.894 (0.710-1.126)
	**$250,000-$499,000**	0.931 (0.751-1.155)
	**$25,000-$149,000**	0.986 (0.790-1.230)
	**<$25,000**	1.109 (0.857-1.435)
**Residency (reference=Urban)**	**Large Rural**	0.797 (0.514-1.235)
	**Small Rural**	0.529 (0.290-0.965)
**Systemic hypertension**		1.161 (0.991-1.360)
**Pure hypercholesterolemia**		1.006 (0.862-1.175)
**Hypertriglyceridemia**		1.057 (0.884-1.264)
**Depression**		1.007 (0.808-1.256)
**Ischemic heart disease**		1.197 (0.969-1.480)
**Other heart disease**		1.244 (1.023-1.513)
**Stroke**		1.235 (0.846-1.802)
**Polycystic ovarian syndrome**		
**Gestational diabetes**		
**Acanthosis nigricans**		
**Hepatitis C**		0.841 (0.650-1.088)
**Osteoarthritis**		1.237 (1.007-1.519)
**Rheumatoid arthritis**		1.270 (0.842-1.916)
**Psoriatic arthritis**		1.276 (0.314-5.179)
**Family history of diabetes**		

NRTI denotes nucleoside reverse-transcriptase inhibitor, NNRTI non-nucleoside reverse-transcriptase inhibitor, PI protease inhibitor, INSTI integrase strand transfer inhibitor, HR hazard

ratio, and CI confidence interval. Hazard ratios based on a Cox proportional-hazards model and adjusted for the confounding variables are presented. Blank cell indicates insufficient evidence to evaluate the regression coefficient and odds ratio.

Supplementary Table 14. Cox proportional hazards model for 1,2,5 and 10 years of follow-up in the Veterans Health Administration Database.

Variable		Within 1 year Adjusted HR (95% CI)	Within 2 years Adjusted HR (95% CI)	Within 5 years Adjusted HR (95% CI)	Within 10 years Adjusted HR (95% CI)
**NRTI use ever**		**0.343 (0.281-0.418)**	**0.510 (0.441-0.591)**	**0.616 (0.556-0.683)**	**0.691 (0.641-0.747)**
**NNRTI use ever**		0.873 (0.699-1.089)	0.795 (0.675-0.937)	1.014 (0.912-1.127)	1.004 (0.929-1.085)
**PI use ever**		1.078 (0.864-1.345)	1.032 (0.875-1.216)	1.209 (1.085-1.347)	1.253 (1.157-1.356)
**INSTI use ever**		0.116 (0.072-0.187)	0.254 (0.201-0.320)	0.337 (0.297-0.383)	0.390 (0.358-0.424)
**Sex: Female**		0.510 (0.335-0.777)	0.596 (0.437-0.812)	0.793 (0.651-0.966)	0.840 (0.730-0.966)
**Race (baseline=White)**	**Black**	1.639 (1.414-1.900)	1.675 (1.488-1.885)	1.607 (1.480-1.745)	1.559 (1.469-1.656)
	**Other**	0.851 (0.709-1.020)	1.000 (0.870-1.150)	0.972 (0.881-1.072)	1.082 (1.008-1.161)
**Charlson comorbidity index**		0.952 (0.931-0.973)	0.986 (0.970-1.003)	1.020 (1.008-1.031)	1.039 (1.031-1.047)
**Lipid lowering medication use**		0.636 (0.528-0.767)	1.010 (0.885-1.153)	1.431 (1.312-1.561)	1.774 (1.667-1.889)
**Antihypertensive medication use**		0.642 (0.555-0.743)	0.783 (0.697-0.879)	0.918 (0.845-0.998)	1.104 (1.036-1.176)
**Tobacco use**		0.684 (0.593-0.788)	0.726 (0.651-0.811)	0.796 (0.739-0.858)	0.937 (0.889-0.988)
**Index age**		1.051 (1.044-1.057)	1.049 (1.044-1.054)	1.041 (1.038-1.045)	1.032 (1.029-1.035)
**Index year**		1.012 (0.998-1.027)	1.048 (1.037-1.060)	1.074 (1.066-1.082)	1.075 (1.069-1.081)
**Viral load**	**0-500**	1.900 (1.524-2.369)	1.630 (1.374-1.934)	1.487 (1.321-1.674)	1.286 (1.176-1.406)
	**501-100**	1.449 (0.820-2.560)	1.360 (0.892-2.075)	1.108 (0.826-1.487)	0.964 (0.775-1.200)
	**>1000**	1.573 (1.276-1.940)	1.352 (1.143-1.600)	1.148 (1.015-1.299)	1.001 (0.911-1.100)
**CD4 counts**	**0-50**	1.536 (1.113-2.121)	1.449 (1.103-1.904)	1.164 (0.933-1.451)	1.079 (0.904-1.289)
	**51-250**	1.323 (1.007-1.736)	1.182 (0.946-1.477)	1.048 (0.893-1.230)	1.076 (0.957-1.210)
	**>250**	1.112 (0.893-1.385)	1.078 (0.915-1.271)	1.040 (0.934-1.158)	0.994 (0.919-1.075)
**BMI (baseline Underweight)**	**Missing**	0.705 (0.465-1.068)	0.902 (0.645-1.260)	1.255 (0.968-1.626)	1.417 (1.154-1.740)
	**Normal**	1.481 (1.024-2.141)	1.326 (0.976-1.803)	1.248 (0.975-1.596)	1.203 (0.987-1.466)
	**Obese**	3.542 (2.419-5.186)	3.313 (2.420-4.536)	3.571 (2.785-4.579)	3.331 (2.731-4.063)
	**Overweight**	1.825 (1.251-2.662)	1.791 (1.312-2.444)	1.835 (1.433-2.349)	1.809 (1.485-2.204)
**Systemic hypertension**		0.888 (0.766-1.030)	0.904 (0.802-1.017)	1.016 (0.933-1.107)	1.148 (1.075-1.226)
**Pure hypercholesterolemia**		2.176 (0.816-5.804)	0.874 (0.495-1.545)	1.079 (0.757-1.536)	1.323 (1.033-1.696)
**Hyperglyceridemia**		0.215 (0.081-0.575)	0.491 (0.278-0.868)	0.512 (0.359-0.729)	0.591 (0.461-0.758)
**Depression**		0.479 (0.412-0.557)	0.553 (0.493-0.621)	0.670 (0.621-0.723)	0.780 (0.740-0.823)
**Ischemic heart disease**		1.195 (1.007-1.419)	1.131 (0.989-1.293)	1.031 (0.942-1.127)	0.956 (0.899-1.017)
**Other heart disease**		0.688 (0.586-0.807)	0.704 (0.621-0.797)	0.700 (0.643-0.761)	0.786 (0.741-0.833)
**Stroke**		0.950 (0.709-1.272)	0.779 (0.618-0.982)	0.678 (0.582-0.791)	0.692 (0.626-0.765)
**Polycystic ovarian syndrome**		1.678 (0.232-12.125)	0.884 (0.123-6.343)	1.151 (0.428-3.098)	1.487 (0.855-2.587)
**Gestational diabetes**		0 (0-1.27E+112)	0 (0-4.562E+62)	0.854 (0.120-6.081)	1.148 (0.369-3.569)
**Acanthosis nigricans**		0.424 (0.300-0.599)	0.513 (0.403-0.653)	0.607 (0.526-0.701)	0.793 (0.728-0.863)
**Hepatitis C**		0.541 (0.456-0.643)	0.622 (0.545-0.710)	0.817 (0.748-0.891)	0.909 (0.854-0.968)
**Osteoarthritis**		0.384 (0.321-0.458)	0.446 (0.391-0.509)	0.542 (0.499-0.589)	0.707 (0.669-0.747)
**Rheumatoid arthritis**		1.170 (0.720-1.903)	1.205 (0.844-1.719)	1.284 (1.030-1.600)	1.247 (1.077-1.443)
**Psoriatic arthritis**		0.686 (0.424-1.110)	0.741 (0.519-1.060)	0.667 (0.523-0.850)	0.826 (0.713-0.957)
**HDL (Baseline=Missing)**	**40-59**	0.765 (0.562-1.043)	0.727 (0.562-0.939)	0.825 (0.676-1.006)	1.051 (0.886-1.245)

	**<40**	1.134 (0.843-1.526)	0.996 (0.777-1.276)	0.986 (0.812-1.199)	1.266 (1.070-1.498)
	**>=60**	0.569 (0.397-0.817)	0.601 (0.449-0.805)	0.687 (0.552-0.856)	0.897 (0.748-1.077)
**Triglycerides (baseline=Missing)**	**150-199**	1.345 (0.947-1.912)	1.447 (1.080-1.937)	1.447 (1.151-1.819)	1.296 (1.068-1.571)
	**200-499**	1.723 (1.227-2.419)	1.901 (1.433-2.523)	1.854 (1.483-2.320)	1.583 (1.309-1.914)
	**<150**	1.097 (0.795-1.513)	1.075 (0.819-1.411)	1.098 (0.883-1.365)	1.034 (0.858-1.245)
	**>=500**	2.779 (1.772-4.360)	3.137 (2.196-4.481)	2.693 (2.063-3.515)	1.999 (1.609-2.484)
**ALT (Baseline=Missing)**	**40-79**	0.963 (0.736-1.259)	0.998 (0.803-1.241)	1.135 (0.967-1.334)	1.072 (0.951-1.208)
	**80-120**	1.130 (0.812-1.571)	1.054 (0.802-1.385)	1.204 (0.986-1.470)	1.150 (0.990-1.335)
	**<40**	0.906 (0.701-1.171)	0.885 (0.719-1.090)	0.893 (0.765-1.043)	0.870 (0.775-0.975)
	**>120**	0.927 (0.640-1.341)	0.919 (0.677-1.248)	1.133 (0.906-1.416)	1.170 (0.990-1.382)
**AST (Baseline=Missing)**	**40-79**	0.644 (0.391-1.059)	0.634 (0.421-0.954)	0.559 (0.400-0.781)	0.523 (0.399-0.687)
	**80-120**	0.710 (0.418-1.207)	0.746 (0.483-1.152)	0.605 (0.425-0.860)	0.526 (0.395-0.701)
	**<40**	0.392 (0.239-0.643)	0.449 (0.300-0.673)	0.475 (0.341-0.660)	0.474 (0.362-0.619)
	**>120**	0.601 (0.349-1.034)	0.607 (0.388-0.952)	0.530 (0.369-0.762)	0.459 (0.342-0.617)
**Bilirubin (Baseline=Missing)**	**2-3**	0.956 (0.545-1.677)	0.691 (0.434-1.099)	0.674 (0.461-0.984)	0.642 (0.473-0.872)
	**<2**	0.732 (0.450-1.191)	0.605 (0.407-0.899)	0.666 (0.480-0.926)	0.671 (0.514-0.877)
	**>3**	0.768 (0.432-1.365)	0.595 (0.370-0.957)	0.648 (0.441-0.953)	0.559 (0.407-0.767)
**Amylase (Baseline=Missing)**	**<=140**	2.613 (2.245-3.041)	2.329 (2.069-2.621)	1.929 (1.781-2.090)	1.485 (1.403-1.572)
	**>140**	2.356 (1.862-2.980)	2.121 (1.755-2.562)	1.825 (1.597-2.086)	1.477 (1.339-1.630)
**Lactate (Baseline=Missing)**	**2-4**	2.616 (2.090-3.274)	2.214 (1.838-2.665)	1.824 (1.595-2.085)	1.766 (1.604-1.944)
	**<2**	2.161 (1.831-2.551)	1.937 (1.698-2.210)	1.683 (1.536-1.843)	1.493 (1.396-1.596)
	**>4**	1.401 (0.900-2.180)	1.548 (1.099-2.181)	1.613 (1.267-2.054)	1.648 (1.378-1.971)
**Family history of diabetes**		0.785 (0.325-1.896)	0.764 (0.396-1.474)	0.854 (0.584-1.249)	1.239 (1.007-1.525)
**Fluoroquinolone use**		1.138 (0.918-1.411)	1.011 (0.849-1.204)	0.975 (0.862-1.102)	1.020 (0.935-1.113)
**Antipsychotic medication use**		1.633 (1.345-1.982)	1.427 (1.216-1.675)	1.327 (1.186-1.484)	1.187 (1.095-1.287)
**Corticosteroid use**		1.029 (0.802-1.320)	1.155 (0.957-1.395)	1.003 (0.876-1.147)	0.879 (0.796-0.971)

NRTI denotes nucleoside reverse-transcriptase inhibitor, NNRTI non-nucleoside reverse-transcriptase inhibitor, PI protease inhibitor, INSTI integrase strand transfer inhibitor, BMI body-mass index (mass in kilograms divided by the square of the height in meters), HDL high-density lipoprotein, ALT alanine aminotransferase, AST aspartate aminotransferase, HR hazard ratio, and CI confidence interval. Viral Load is measured in RNA copies per milliliter and CD4 count in cells per cubic millimeter. HDL, Triglycerides, and Bilirubin are measured in milligrams per deciliter. AST, ALT, and Amylase are measured in units per liter. Hazard ratios based on a Cox proportional-hazards model and adjusted for the confounding variables are presented.

Supplementary Table 15. Follow-up duration and mortality rates in the Veterans Health Administration Database.

Factor		Total follow-up (Patient-years)	Deaths (%)
**NRTI**	**Never (N=37,227)**	369,263.2	8,421 (22.6)
	**Ever (N=42,517)**	407,581.1	10,625 (25.0)

Supplementary Table 16. Subdistribution Hazard of Incident Diabetes for NRTI use (ever/never) under Competing Risk of Death in the Veterans Health Administration Database.

Variable		Adjusted HR (95% CI)
**NRTI use ever**		0.727 (0.683-0.775)
**NNRTI use ever**		1.008 (0.952-1.067)
**PI use ever**		1.095 (1.032-1.162)
**INSTI use ever**		0.683 (0.646-0.722)
**Sex: Female**		1.034 (0.945-1.132)
**Race (baseline=White)**	**Black**	1.456 (1.394-1.521)
	**Other**	1.168 (1.108-1.232)
**Charlson comorbidity index**		1.048 (1.042-1.053)
**Lipid lowering medication use**		2.115 (2.014-2.221)
**Antihypertensive medication use**		1.147 (1.092-1.205)
**Tobacco use**		0.910 (0.875-0.945)
**Index age**		1.010 (1.008-1.012)
**Index year**		1.120 (1.114-1.126)
**Viral load**	**0-500**	1.054 (0.980-1.134)
	**501-100**	0.879 (0.758-1.019)
	**>1000**	0.856 (0.793-0.925)
**CD4 counts**	**0-50**	0.859 (0.740-0.997)
	**51-250**	0.874 (0.799-0.957)
	**>250**	0.908 (0.857-0.961)
**BMI (baseline Underweight)**	**Missing**	1.657 (1.383-1.986)
	**Normal**	1.379 (1.155-1.648)
	**Obese**	3.234 (2.706-3.865)
	**Overweight**	2.036 (1.705-2.430)
**Systemic hypertension**		1.416 (1.341-1.494)
**Pure hypercholesterolemia**		1.017 (0.85-1.216)
**Hyperglyceridemia**		0.960 (0.801-1.15)
**Depression**		0.862 (0.83-0.896)
**Ischemic heart disease**		0.913 (0.873-0.954)
**Other heart disease**		0.819 (0.785-0.854)
**Stroke**		0.763 (0.714-0.816)
**Polycystic ovarian syndrome**		1.189 (0.754-1.875)
**Gestational diabetes**		1.551 (0.858-2.803)
**Acanthosis nigricans**		1.055 (1.004-1.108)
**Hepatitis C**		0.936 (0.894-0.980)
**Osteoarthritis**		0.930 (0.894-0.966)
**Rheumatoid arthritis**		1.086 (0.980-1.203)
**Psoriatic arthritis**		0.895 (0.815-0.982)

**HDL (Baseline=Missing)**	**40-59**	1.235 (1.067-1.428)
	**<40**	1.395 (1.207-1.612)
	**>=60**	1.069 (0.917-1.247)
**Triglycerides (baseline=Missing)**	**150-199**	1.629 (1.348-1.968)
	**200-499**	1.939 (1.606-2.340)
	**<150**	1.407 (1.169-1.694)
	**>=500**	2.429 (1.991-2.963)
**ALT (Baseline=Missing)**	**40-79**	1.130 (1.028-1.243)
	**80-120**	1.230 (1.094-1.382)
	**<40**	0.940 (0.858-1.029)
	**>120**	1.322 (1.160-1.507)
**AST (Baseline=Missing)**	**40-79**	1.181 (0.851-1.64)
	**80-120**	1.041 (0.743-1.458)
	**<40**	1.215 (0.876-1.684)
	**>120**	0.966 (0.686-1.360)
**Bilirubin (Baseline=Missing)**	**2--3**	1.138 (0.828-1.566)
	**<2**	1.276 (0.954-1.705)
	**>3**	1.141 (0.824-1.580)
**Amylase (Baseline=Missing)**	**<=140**	1.129 (1.084-1.176)
	**>140**	1.111 (1.029-1.199)
**Lactate (Baseline=Missing)**	**2--4**	1.395 (1.291-1.508)
	**<2**	1.255 (1.193-1.320)
	**>4**	1.155 (0.975-1.368)
**Family history of diabetes**		1.510 (1.335-1.707)
**Fluoroquinolone use**		1.018 (0.952-1.089)
**Antipsychotic medication use**		1.126 (1.061-1.195)
**Corticosteroid use**		0.912 (0.843-0.986)

NRTI denotes nucleoside reverse-transcriptase inhibitor, NNRTI non-nucleoside reverse-transcriptase inhibitor, PI protease inhibitor, INSTI integrase strand transfer inhibitor, BMI body-mass index (mass in kilograms divided by the square of the height in meters), HDL high-density lipoprotein, ALT alanine aminotransferase, AST aspartate aminotransferase, HR hazard ratio, and CI confidence interval. Viral Load is measured in RNA copies per milliliter and CD4 count in cells per cubic millimeter. HDL, Triglycerides, and Bilirubin are measured in milligrams per deciliter. AST, ALT, and Amylase are measured in units per liter. Hazard ratios based on a Cox proportional-hazards model and adjusted for the confounding variables are presented.

Supplementary Table 17. Counts and percent by cohort and NRTI exposure of Veterans Health Administration database.

Cohort	NRTI exposure	Patients (%)
**HIV– / Hepatitis B+**	**Never**	17,399 (81%)
	**Ever**	4,038 (19%)
**HIV+ / Hepatitis B –**	**Never**	16,914 (34%)
	**Ever**	32,514 (66%)
**HIV+ / Hepatitis B +**	**Never**	2,914 (33%)
	**Ever**	5,965 (67%)

Supplementary Table 18. Counts and percent by cohort and NRTI exposure in Truven Marketscan database.

Cohort	NRTI exposure	Patients (%)
**HIV– / Hepatitis B+**	**Never**	12,206 (82%)
	**Ever**	2,600 (11%)
**HIV+ / Hepatitis B –**	**Never**	96 (1%)
	**Ever**	8,197 (99%)
**HIV+ / Hepatitis B +**	**Never**	6 (1%)
	**Ever**	529 (99%)

Supplementary Table 19. Counts and percent by cohort and NRTI exposure in PearlDiver database.

Cohort	NRTI exposure	Patients (%)
**HIV– / Hepatitis B+**	**Never**	4,657 (79.8%)
	**Ever**	1,180 (20.2%)
**HIV+ / Hepatitis B –**	**Never**	1,654 (17.0%)
	**Ever**	8,091 (83.0%)
**HIV+ / Hepatitis B +**	**Never**	49 (10.6%)
	**Ever**	414 (89.4%)

Supplementary Table 20. Counts and percent by cohort and NRTI exposure in Medicare database.

Cohort	NRTI exposure	Patients (%)
**HIV– / Hepatitis B+**	**Never**	1,650 (84.1%)
	**Ever**	313 (15.9%)
**HIV+ / Hepatitis B –**	**Never**	171 (16.4%)
	**Ever**	874 (83.6%)
**HIV+ / Hepatitis B +**	**Never**	39 (43.8%)
	**Ever**	50 (56.2%)

Supplementary Table 21. Counts and percent by cohort and NRTI exposure in Clinformatics database.

Cohort	NRTI exposure	Patients (%)
**HIV– / Hepatitis B+**	**Never**	3,213 (87.1%)
	**Ever**	476 (12.9%)
**HIV+ / Hepatitis B –**	**Never**	582 (23.9%)
	**Ever**	1,857 (76.1%)
**HIV+ / Hepatitis B +**	**Never**	50 (23.5%)
	**Ever**	163 (76.5%)

Supplementary Table 22. Hazard of Incident Diabetes for NRTI use (ever/never) among HIV-positive Hepatitis B-negative persons in the Veterans Health Administration Database.

Variable		Adjusted HR (95% CI)
**NRTI use ever**		0.621 (0.562-0.685)
**NNRTI use ever**		1.150 (1.074-1.231)
**PI use ever**		1.299 (1.212-1.392)
**INSTI use ever**		0.473 (0.444-0.504)
**Sex: Female**		1.022 (0.908-1.151)
**Race (baseline=White)**	**Black**	1.399 (1.318-1.485)
	**Other**	1.146 (1.063-1.235)
**Charlson comorbidity index**		1.106 (1.097-1.115)
**Lipid lowering medication**		1.805 (1.691-1.926)
**Antihypertensive medication**		1.254 (1.175-1.339)
**Tobacco use**		0.953 (0.905-1.004)
**Index Age**		1.029 (1.026-1.032)
**Index year**		1.234 (1.224-1.245)
**Viral load**	**0-500**	0.936 (0.852-1.027)
	**501-100**	0.802 (0.672-0.956)
	**>1000**	0.883 (0.799-0.975)
**CD4 counts**	**0-50**	1.010 (0.859-1.189)
	**51-250**	0.893 (0.807-0.989)
	**>250**	0.882 (0.827-0.940)
**BMI (baseline=Underweight)**	**Missing**	1.028 (0.821-1.287)
	**Normal**	1.028 (0.825-1.281)
	**Obese**	2.319 (1.859-2.894)
	**Overweight**	1.396 (1.121-1.739)
**Systemic Hypertension**		1.303 (1.215-1.398)
**Pure hypercholesterolemia**		1.108 (0.875-1.403)
**Hyperglyceridemia**		0.751 (0.592-0.953)
**Depression**		0.862 (0.818-0.909)
**Ischemic Heart Disease**		0.933 (0.878-0.991)
**Other Heart Disease**		0.846 (0.799-0.896)
**Stroke**		0.708 (0.644-0.778)
**Polycystic ovarian syndrome**		1.544 (1.030-2.315)
**Gestational diabetes**		1.718 (0.855-3.452)
**Acanthosis nigricans**		0.922 (0.860-0.989)
**Hepatitis C**		0.838 (0.781-0.899)
**Osteoarthritis**		0.800 (0.759-0.844)
**Rheumatoid Arthritis**		0.982 (0.833-1.158)
**Psoriatic Arthritis**		0.881 (0.771-1.007)

**HDL (Baseline=Missing)**	**40-59**	0.820 (0.676-0.995)
	**<40**	1.019 (0.842-1.234)
	**>=60**	0.712 (0.580-0.874)
**Triglycerides (baseline=Missing)**	**150-199**	1.021 (0.797-1.309)
	**200-499**	1.246 (0.974-1.593)
	**<150**	0.904 (0.709-1.153)
	**>=500**	1.521 (1.177-1.965)
**ALT (Baseline=Missing)**	**40-79**	1.014 (0.887-1.158)
	**80-120**	0.948 (0.798-1.128)
	**<40**	0.840 (0.740-0.954)
	**>120**	0.980 (0.798-1.203)
**AST (Baseline=Missing)**	**40-79**	1.677 (1.015-2.770)
	**80-120**	1.859 (1.108-3.118)
	**<40**	1.459 (0.886-2.401)
	**>120**	1.758 (1.035-2.985)
**Bilirubin (Baseline=Missing)**	**2-3**	2.095 (1.253-3.505)
	**<2**	1.860 (1.146-3.020)
	**>3**	2.171 (1.265-3.725)
**Amylase (Baseline=Missing)**	**<=140**	1.174 (1.110-1.241)
	**>140**	1.268 (1.149-1.400)
**Lactate (Baseline=Missing)**	**2-4**	1.435 (1.299-1.585)
	**<2**	1.159 (1.086-1.238)
	**>4**	1.977 (1.615-2.419)
**Family history of diabetes**		1.360 (1.130-1.637)
**Fluoroquinolone use**		1.043 (0.951-1.144)
**Antipsychotic medication use**		1.150 (1.059-1.249)
**Corticosteroid use**		0.960 (0.863-1.067)

NRTI denotes nucleoside reverse-transcriptase inhibitor, NNRTI non-nucleoside reverse-transcriptase inhibitor, PI protease inhibitor, INSTI integrase strand transfer inhibitor, BMI body-mass index (mass in kilograms divided by the square of the height in meters), HDL high-density lipoprotein, ALT alanine aminotransferase, AST aspartate aminotransferase, HR hazard ratio, and CI confidence interval. Viral Load is measured in RNA copies per milliliter and CD4 count in cells per cubic millimeter. HDL, Triglycerides, and Bilirubin are measured in milligrams per deciliter. AST, ALT, and Amylase are measured in units per liter. Hazard ratios based on a Cox proportional-hazards model and adjusted for the confounding variables are presented.

Supplementary Table 23. Hazard of Incident Diabetes for NRTI use (ever/never) among HIV-negative Hepatitis B-positive persons in the Veterans Health Administration Database.

Variable		Adjusted HR (95% CI)
**NRTI use ever**		0.717 (0.656-0.783)
**Sex: Female**		0.954 (0.800-1.137)
**Race (baseline=White)**	**Black**	1.454 (1.347-1.570)
	**Other**	1.187 (1.087-1.297)
**Charlson comorbidity index**		1.098 (1.087-1.11)
**Lipid lowering medication**		1.690 (1.559-1.832)
**Antihypertensive medication**		1.024 (0.939-1.117)
**Tobacco use**		1.021 (0.954-1.093)
**Index age**		1.021 (1.017-1.025)
**Index year**		1.254 (1.239-1.268)
**BMI (baseline=Underweight)**	**Missing**	1.166 (0.823-1.653)
	**Normal**	0.889 (0.632-1.251)
	**Obese**	2.076 (1.480-2.912)
	**Overweight**	1.315 (0.938-1.843)
**Systemic Hypertension**		1.272 (1.154-1.403)
**Pure hypercholesterolemia**		1.222 (0.898-1.664)
**Hyperglyceridemia**		0.692 (0.507-0.943)
**Depression**		0.816 (0.763-0.874)
**Ischemic Heart Disease**		0.937 (0.870-1.009)
**Other Heart Disease**		0.861 (0.802-0.924)
**Stroke**		0.737 (0.661-0.823)
**Polycystic ovarian syndrome**		1.459 (0.650-3.275)
**Gestational diabetes**		0.682 (0.096-4.861)
**Acanthosis nigricans**		0.927 (0.848-1.013)
**Hepatitis C**		0.835 (0.777-0.898)
**Osteoarthritis**		0.805 (0.754-0.859)
**Rheumatoid Arthritis**		1.067 (0.917-1.242)
**Psoriatic Arthritis**		0.950 (0.814-1.109)
**HDL (Baseline=Missing)**	**40-59**	0.785 (0.605-1.017)
	**<40**	0.936 (0.723-1.213)
	**>=60**	0.694 (0.530-0.910)
**Triglycerides (baseline=Missing)**	**150-199**	0.877 (0.634-1.214)
	**200-499**	0.969 (0.702-1.338)
	**<150**	0.731 (0.533-1.003)
	**>=500**	1.218 (0.847-1.751)
**ALT (Baseline=Missing)**	**40-79**	0.977 (0.838-1.139)
	**80-120**	1.045 (0.870-1.254)

	**<40**	0.835 (0.719-0.969)
	**>120**	0.813 (0.667-0.991)
**AST (Baseline=Missing)**	**40-79**	2.782 (1.289-6.009)
	**80-120**	3.092 (1.420-6.734)
	**<40**	2.514 (1.166-5.418)
	**>120**	3.135 (1.434-6.855)
**Bilirubin (Baseline=Missing)**	**2-3**	0.903 (0.487-1.673)
	**<2**	0.848 (0.476-1.510)
	**>3**	0.768 (0.416-1.419)
**Amylase (Baseline=Missing)**	**<=140**	1.193 (1.111-1.282)
	**>140**	1.097 (0.943-1.277)
**Lactate (Baseline=Missing)**	**2-4**	1.577 (1.378-1.805)
	**<2**	1.180 (1.074-1.296)
	**>4**	1.424 (1.080-1.877)
**Family history of diabetes**		1.361 (1.106-1.676)
**Fluoroquinolone use**		1.125 (1.011-1.252)
**Antipsychotic medication use**		1.146 (1.037-1.265)
**Corticosteroid use**		0.937 (0.828-1.060)

NRTI denotes nucleoside reverse-transcriptase inhibitor, BMI body-mass index (mass in kilograms divided by the square of the height in meters), HDL high-density lipoprotein, ALT alanine aminotransferase, AST aspartate aminotransferase, HR hazard ratio, and CI confidence interval. Viral Load is measured in RNA copies per milliliter and CD4 count in cells per cubic millimeter. HDL, Triglycerides, and Bilirubin are measured in milligrams per deciliter. AST, ALT, and Amylase are measured in units per liter. Hazard ratios based on a Cox proportional-hazards model and adjusted for the confounding variables are presented.

Supplementary Table 24. Hazard of Incident Diabetes for NRTI use (per year of exposure) in the Veterans Health Administration Database.

Variable		Adjusted HR (95% CI)
**NRTI use per year**		0.975 (0.969-0.982)
**NNRTI use per year**		0.910 (0.885-0.936)
**PI use per year**		0.874 (0.848-0.901)
**INSTI use per year**		1.072 (1.053-1.092)
**Sex: Female**		0.960 (0.874-1.054)
**Race (baseline=White)**	**Black**	1.417 (1.357-1.479)
	**Other**	1.209 (1.147-1.275)
**Charlson comorbidity index**		1.083 (1.077-1.089)
**Lipid lowering medication use**		1.728 (1.650-1.810)
**Antihypertensive medication use**		1.200 (1.143-1.259)
**Tobacco use**		1.006 (0.968-1.045)
**Index age**		1.031 (1.029-1.034)
**Index year**		1.208 (1.200-1.216)
**Viral load**	**0-500**	0.724 (0.678-0.772)
	**501-100**	0.576 (0.498-0.665)
	**>1000**	0.628 (0.587-0.671)
**CD4 counts**	**0-50**	0.949 (0.820-1.098)
	**51-250**	0.871 (0.798-0.951)
	**>250**	0.834 (0.789-0.882)
**BMI (baseline Underweight)**	**Missing**	0.971 (0.819-1.153)
	**Normal**	0.941 (0.796-1.113)
	**Obese**	2.119 (1.791-2.506)
	**Overweight**	1.290 (1.092-1.524)
**Systemic hypertension**		1.262 (1.197-1.330)
**Pure hypercholesterolemia**		1.181 (0.994-1.404)
**Hyperglyceridemia**		0.736 (0.619-0.875)
**Depression**		0.818 (0.787-0.850)
**Ischemic heart disease**		0.922 (0.883-0.963)
**Other heart disease**		0.851 (0.817-0.887)
**Stroke**		0.726 (0.680-0.776)
**Polycystic ovarian syndrome**		1.495 (1.056-2.118)
**Gestational diabetes**		1.717 (0.921-3.201)
**Acanthosis nigricans**		0.928 (0.882-0.976)
**Hepatitis C**		0.866 (0.828-0.906)
**Osteoarthritis**		0.805 (0.775-0.837)
**Rheumatoid arthritis**		1.038 (0.935-1.154)
**Psoriatic arthritis**		0.866 (0.788-0.952)
**HDL (Baseline=Missing)**	**40-59**	0.833 (0.722-0.962)

	**<40**	1.008 (0.874-1.163)
	**>=60**	0.734 (0.631-0.854)
**Triglycerides (baseline=Missing)**	**150-199**	1.014 (0.840-1.223)
	**200-499**	1.191 (0.988-1.434)
	**<150**	0.873 (0.727-1.049)
	**>=500**	1.405 (1.154-1.711)
**ALT (Baseline=Missing)**	**40-79**	0.989 (0.899-1.088)
	**80-120**	1.004 (0.893-1.129)
	**<40**	0.822 (0.749-0.901)
	**>120**	0.903 (0.792-1.030)
**AST (Baseline=Missing)**	**40-79**	1.966 (1.314-2.944)
	**80-120**	2.166 (1.437-3.267)
	**<40**	1.830 (1.225-2.734)
	**>120**	2.101 (1.388-3.182)
**Bilirubin (Baseline=Missing)**	**2--3**	1.509 (1.029-2.211)
	**<2**	1.432 (0.998-2.054)
	**>3**	1.474 (1.000-2.172)
**Amylase (Baseline=Missing)**	**<=140**	1.205 (1.157-1.255)
	**>140**	1.220 (1.133-1.314)
**Lactate (Baseline=Missing)**	**2--4**	1.448 (1.347-1.557)
	**<2**	1.156 (1.101-1.214)
	**>4**	1.770 (1.524-2.055)
**Family history of diabetes**		1.402 (1.231-1.596)
**Fluoroquinolone use**		1.065 (0.997-1.137)
**Antipsychotic medication use**		1.152 (1.087-1.222)
**Corticosteroid use**		0.939 (0.859-1.026)

NRTI denotes nucleoside reverse-transcriptase inhibitor, NNRTI non-nucleoside reverse-transcriptase inhibitor, PI protease inhibitor, INSTI integrase strand transfer inhibitor, BMI body-mass index (mass in kilograms divided by the square of the height in meters), HDL high-density lipoprotein, ALT alanine aminotransferase, AST aspartate aminotransferase, HR hazard ratio, and CI confidence interval. Viral Load is measured in RNA copies per milliliter and CD4 count in cells per cubic millimeter. HDL, Triglycerides, and Bilirubin are measured in milligrams per deciliter. AST, ALT, and Amylase are measured in units per liter. Hazard ratios based on a Cox proportional-hazards model and adjusted for the confounding variables are presented.

Supplementary Table 25. Hazard of Incident Diabetes for NRTI use (per year of exposure) in the Truven Marketscan Database.

Variable	Adjusted HR (95% CI)
**NRTI use (per year)**	0.923 (0.864-0.986)
**NNRTI use (per year)**	0.882 (0.815-0.954)
**PI use (per year)**	0.872 (0.804-0.945)
**INSTI use (per year)**	0.883 (0.778-1.003)
**Index Age**	0.977 (0.948-1.007)
**Sex: Male**	0.972 (0.867-1.090)
**Charlson comorbidity index**	1.151 (1.138-1.165)
**Index year**	0.887 (0.870-0.904)
**Depression**	0.924 (0.815-1.048)
**Systemic hypertension**	1.261 (1.127-1.410)
**Hyperglyceridemia**	1.131 (1.005-1.272)
**Stroke**	0.898 (0.762-1.058)
**Polycystic ovarian syndrome**	0.906 (0.515-1.592)
**Gestational diabetes**	2.041 (0.326-12.796)
**Acanthosis nigricans**	0.616 (0.499-0.760)
**Hepatitis C**	0.862 (0.748-0.992)
**Osteoarthritis**	0.831 (0.740-0.933)
**Rheumatoid arthritis**	1.181 (0.936-1.49)
**Psoriatic arthritis**	0.779 (0.571-1.063)
**Family history of diabetes**	0.910 (0.515-1.606)
**Fluoroquinolone use**	1.090 (0.936-1.269)
**Corticosteroid use**	0.933 (0.788-1.104)
**Antihypertensive medication use**	1.188 (1.064-1.326)
**Lipid lowering medication use**	1.411 (1.223-1.628)

NRTI denotes nucleoside reverse-transcriptase inhibitor, NNRTI non-nucleoside reverse-transcriptase inhibitor, PI protease inhibitor, INSTI integrase strand transfer inhibitor, HR hazard ratio, and CI confidence interval. Hazard ratios based on a Cox proportional-hazards model and adjusted for the confounding variables are presented.

Supplementary Table 26. Hazard of Incident Diabetes for NRTI use (per year of exposure) in the PearlDiver Database *.

Variable		Adjusted HR(95% CI)
**NRTI Use (Per year of exposure)**		0.785 (0.666-0.925)
**NNRTI Use (Per year of exposure)**		0.856 (0.730-1.003)
**PI Use (Per year of exposure)**		0.880 (0.757-1.022)
**INSTI Use (Per year of exposure)**		0.884 (0.737-1.061)
**Sex (reference=Female)**	**Male**	0.954 (0.815-1.116)
**Race (reference=White)**	**Black**	1.318 (1.082-1.554)
	**Hispanic**	1.157 (0.841-1.474)
**Charlson comorbidity index**		1.110 (1.088-1.133)
**Tobacco use**		0.738 (0.629-0.866)
**Index age (Per year)**		0.972 (0.937-1.007)
**Index year (Per year)**		1.027 (0.963-1.095)
**Viral Load (reference=missing)**	**0-500**	1.052 (0.837-1.323)
	**501-1000**	0.599 (0.260-1.380)
	**>1000**	0.697 (0.095-5.095)
**CD4 count (reference=missing)**	**0-50**	0.634 (0.400-1.005)
	**51-250**	1.187 (0.862-1.634)
	**>250**	0.818 (0.634-1.056)
**BMI (reference=normal)**	**30-40**	1.362 (1.157-1.605)
	**>40**	1.773 (1.447-2.173)
**Systemic hypertension**		2.297 (1.688-3.125)
**Pure hypercholesterolemia**		1.099 (0.940-1.287)
**Hyperglyceridemia**		1.842 (1.386-2.448)
**Depression**		1.014 (0.871-1.179)
**Ischemic heart disease**		1.145 (0.975-1.345)
**Other heart disease**		1.167 (0.993-1.373)
**Stroke**		0.863 (0.706-1.055)
**Polycystic ovarian syndrome**		1.785 (0.554-5.748)
**Gestational diabetes**		25.20 (8.316-76.35)
**Acanthosis nigricans**		1.212 (0.383-3.835)
**Hepatitis C**		1.255 (0.992-1.589)
**Osteoarthritis**		1.070 (0.925-1.239)
**Rheumatoid arthritis**		0.871 (0.680-1.118)
**Psoriatic arthritis**		1.327 (0.673-2.619)
**HDL (Baseline=Missing)**	**40-59**	1.015 (0.858-1.202)
	**<40**	1.411 (1.176-1.692)
	**>=60**	0.961 (0.803-1.150)
**Triglycerides (Baseline=Missing)**	**150-199**	0.960 (0.814-1.132)
	**200-499**	1.138 (0.955-1.356)

	**<150**	0.929 (0.758-1.139)
	**>=500**	1.154 (0.866-1.539)
**ALT (Baseline=Missing)**	**40-79**	1.133 (0.938-1.369)
	**80-120**	1.099 (0.856-1.412)
	**<40**	1.005 (0.686-1.534)
	**>120**	1.165 (0.853-1.592)
**AST (Baseline=Missing)**	**40-79**	1.021 (0.841-1.239)
	**80-120**	1.076 (0.824-1.406)
	**<40**	0.994 (0.643-1.538)
	**>120**	0.791 (0.570-1.097)
**Bilirubin (Baseline=Missing)**	**2-3**	0.967 (0.713-1.313)
	**<2**	0.753 (0.505-1.122)
	**>3**	0.970 (0.676-1.392)
**Amylase (Baseline=Missing)**	**<=140**	1.059 (0.872-1.285)
	**>140**	0.813 (0.498-1.328)
**Family history of diabetes**		1.628 (1.184-2.238)
**Fluoroquinolone use**		1.029 (0.889-1.191)
**Antipsychotic medication use**		1.147 (0.961-1.370)
**Corticosteroid use**		0.836 (0.721-0.970)
**Antihypertensive medication use**		1.414 (1.129-1.772)
**Lipid lowering medication use**		1.498 (1.270-1.766)

NRTI denotes nucleoside reverse-transcriptase inhibitor, NNRTI non-nucleoside reverse-transcriptase inhibitor, PI protease inhibitor, INSTI integrase strand transfer inhibitor, BMI body-mass index (mass in kilograms divided by the square of the height in meters), HDL high-density lipoprotein, ALT alanine aminotransferase, AST aspartate aminotransferase, HR hazard ratio, and CI confidence interval. Viral Load is measured in RNA copies per milliliter and CD4 count in cells per cubic millimeter. HDL, Triglycerides, and Bilirubin are measured in milligrams per deciliter. AST, ALT, and Amylase are measured in units per liter. Hazard ratios based on a Cox proportional-hazards model and adjusted for the confounding variables are presented.

Supplementary Table 27. Hazard of Incident Diabetes for NRTI use (per year of exposure) in the Medicare Database.

Variable		Adjusted HR (95% CI)
**NRTI Use (Per year of exposure)**		0.891 (0.794-1.000)
**NNRTI Use (Per year of exposure)**		0.982 (0.859-1.124)
**PI Use (Per year of exposure)**		1.025 (0.899-1.169)
**ISTI Use (Per year of exposure)**		0.951 (0.806-1.122)
**Sex (reference=Male)**	**Female**	1.083 (0.925-1.267)
**Race (reference =White)**	**Black**	1.477 (1.158-1.884)
	**Latino/Hispanic**	2.227 (1.511-3.283)
	**Asian**	1.860 (1.516-2.281)
**Charlson comorbidity index**		1.051 (1.019-1.084)
**Index age (Per year)**		0.979 (0.964-0.994)
**Index year (Per year)**		0.898 (0.835-0.967)
**Systemic hypertension**		1.237 (1.031-1.484)
**Pure hypercholesterolemia**		1.244 (1.058-1.463)
**Hypertriglyceridemia**		1.204 (1.004-1.445)
**Depression**		1.273 (0.855-1.897)
**Ischemic heart disease**		1.485 (1.256-1.756)
**Other heart disease**		1.105 (0.937-1.304)
**Stroke**		1.346 (1.099-1.648)
**Polycystic ovarian syndrome**		
**Gestational diabetes**		
**Acanthosis nigricans**		8.495 (2.043-35.32)
**Hepatitis C**		0.896 (0.683-1.175)
**Osteoarthritis**		1.265 (1.079-1.483)
**Rheumatoid arthritis**		0.895 (0.624-1.284)
**Psoriatic arthritis**		
**Family history of diabetes**		

NRTI denotes nucleoside reverse-transcriptase inhibitor, NNRTI non-nucleoside reverse-transcriptase inhibitor, PI protease inhibitor, INSTI integrase strand transfer inhibitor, HR hazard ratio, and CI confidence interval. Hazard ratios based on a Cox proportional-hazards model and adjusted for the confounding variables are presented. Blank cell indicates insufficient evidence to evaluate the regression coefficient and odds ratio.

Supplementary Table 28. Hazard of Incident Diabetes for NRTI use (per year of exposure) in the Clinformatics Database.

Variable		Adjusted HR (95% CI)
**NRTI Use (Per year of exposure)**		0.918 (0.830-1.015)
**NNRTI Use (Per year of exposure)**		0.908 (0.801-1.028)
**PI Use (Per year of exposure)**		0.974 (0.868-1.094)
**ISTI Use (Per year of exposure)**		1.126 (0.961-1.318)
**Sex (reference=Male)**	**Female**	1.018 (0.883-1.173)
**Race (reference =White)**	**Black**	1.462 (1.217-1.756)
	**Latino/Hispanic**	1.384 (1.098-1.746)
	**Asian**	1.211 (1.008-1.455)
**Charlson comorbidity index**		1.000 (0.976-1.025)
**Index age (Per year)**		1.007 (0.997-1.018)
**Index year (Per year)**		0.958 (0.939-0.978)
**Education (reference=Some college)**	**Bachelor or more**	0.854 (0.695-1.049)
	**High school diploma**	1.098 (0.939-1.283)
	**Less than High school**	1.863 (1.058-3.280)
**Household Net Worth (reference=$150 -$249K)**	**>$500,000**	0.888 (0.705-1.119)
	**$250,000-$499,000**	0.927 (0.748-1.150)
	**$25,000-$149,000**	0.987 (0.791-1.231)
	**<$25,000**	1.108 (0.856-1.434)
**Residency (reference=Urban)**	**Large Rural**	0.801 (0.517-1.242)
	**Small Rural**	0.532 (0.292-0.970)
**Systemic hypertension**		1.159 (0.989-1.358)
**Pure hypercholesterolemia**		1.009 (0.864-1.178)
**Hypertriglyceridemia**		1.065 (0.890-1.273)
**Depression**		1.009 (0.810-1.258)
**Ischemic heart disease**		1.201 (0.972-1.483)
**Other heart disease**		1.243 (1.022-1.512)
**Stroke**		1.225 (0.839-1.788)
**Polycystic ovarian syndrome**		
**Gestational diabetes**		
**Acanthosis nigricans**		
**Hepatitis C**		0.836 (0.646-1.080)
**Osteoarthritis**		1.244 (1.014-1.528)
**Rheumatoid arthritis**		1.268 (0.840-1.912)
**Psoriatic arthritis**		1.287 (0.317-5.222)
**Family history of diabetes**		

NRTI denotes nucleoside reverse-transcriptase inhibitor, NNRTI non-nucleoside reverse-transcriptase inhibitor, PI protease inhibitor, INSTI integrase strand transfer inhibitor, HR hazard

ratio, and CI confidence interval. Hazard ratios based on a Cox proportional-hazards model and adjusted for the confounding variables are presented. Blank cell indicates insufficient evidence to evaluate the regression coefficient and odds ratio.

Supplementary Table 29. Hazard of Incident Diabetes in the Veterans Health Administration Database among Propensity-Score Matched Participants*.

Variable		Adjusted HR (95% CI)
**NRTI Use (Per year of exposure)**		0.979 (0.959-0.999)
**NNRTI Use (Per year of exposure)**		0.866 (0.744-1.006)
**PI Use (Per year of exposure)**		0.817 (0.710-0.941)
**INSTI Use (Per year of exposure)**		1.052 (0.936-1.181)
**Sex (reference=Male)**	**Female**	1.211 (1.008-1.453)
**Race (reference=White)**	**Black**	1.369 (1.236-1.517)
	**Other**	1.109 (0.978-1.257)
**Charlson comorbidity index**		1.078 (1.066-1.091)
**Lipid lowering medication use**		1.626 (1.463-1.808)
**Antihypertensive use**		1.289 (1.140-1.458)
**Tobacco use**		0.934 (0.853-1.023)
**Index age (Per year)**		1.031 (1.026-1.036)
**Index year (Per year)**		1.258 (1.231-1.285)
**Viral Load (reference=missing)**	**0-500**	0.830 (0.720-0.957)
	**501-1000**	0.760 (0.493-1.171)
	**>1000**	0.841 (0.709-0.997)
**CD4+ count (reference=missing)**	**0-50**	1.920 (1.316-2.802)
	**51-250**	1.266 (0.906-1.771)
	**>250**	0.808 (0.688-0.948)
**BMI (reference<18.5)**	**Missing**	0.699 (0.459-1.064)
	**18.5-24.9**	0.677 (0.450-1.018)
	**25-29.9**	0.945 (0.629-1.418)
	**≥30**	1.487 (0.988-2.236)
**Systemic hypertension**		1.181 (1.040-1.341)
**Pure hypercholesterolemia**		1.296 (0.925-1.816)
**Hyperglyceridemia**		0.731 (0.521-1.026)
**Depression**		0.826 (0.756-0.904)
**Ischemic heart disease**		0.881 (0.792-0.981)
**Other heart disease**		0.900 (0.814-0.996)
**Stroke**		0.682 (0.579-0.803)
**Polycystic ovarian syndrome**		1.630 (0.874-3.039)
**Gestational diabetes**		2.782 (1.049-7.375)
**Acanthosis nigricans**		1.064 (0.949-1.193)
**Hepatitis C**		0.787 (0.709-0.873)
**Osteoarthritis**		0.814 (0.741-0.894)
**Rheumatoid arthritis**		1.335 (1.108-1.609)
**Psoriatic arthritis**		0.955 (0.779-1.170)
**HDL (Baseline=Missing)**	**40-59**	0.708 (0.493-1.015)

	**<40**	0.889 (0.620-1.273)
	**>=60**	0.635 (0.436-0.925)
**Triglycerides (Baseline=Missing)**	**150-199**	1.307 (0.859-1.989)
	**200-499**	1.375 (0.905-2.091)
	**<150**	1.110 (0.735-1.676)
	**>=500**	1.896 (1.203-2.988)
**ALT (Baseline=Missing)**	**40-79**	0.973 (0.788-1.201)
	**80-120**	1.055 (0.821-1.356)
	**<40**	0.831 (0.678-1.019)
	**>120**	1.092 (0.837-1.426)
**AST (Baseline=Missing)**	**40-79**	0.865 (0.478-1.565)
	**80-120**	0.768 (0.414-1.422)
	**<40**	0.821 (0.457-1.476)
	**>120**	0.999 (0.535-1.867)
**Bilirubin (Baseline=Missing)**	**2-3**	1.171 (0.608-2.257)
	**<2**	1.380 (0.764-2.492)
	**>3**	1.100 (0.567-2.135)
**Amylase (Baseline=Missing)**	**<=140**	1.175 (1.066-1.295)
	**>140**	1.513 (1.273-1.799)
**Lactate (Baseline=Missing)**	**2-4**	1.562 (1.301-1.874)
	**<2**	1.091 (0.967-1.232)
	**>4**	1.747 (1.128-2.706)
**Family history of diabetes**		1.336 (0.996-1.791)
**Fluoroquinolone use**		0.996 (0.857-1.157)
**Antipsychotic medication use**		0.921 (0.784-1.082)
**Corticosteroid use**		0.910 (0.774-1.070)

* The propensity-score-matched cohort included 9,057 patients in the NRTI-exposed group and 9,057 patients in the NRTI-non-exposed group. NRTI denotes nucleoside reverse-transcriptase inhibitor, NNRTI non-nucleoside reverse-transcriptase inhibitor, PI protease inhibitor, INSTI integrase strand transfer inhibitor, BMI body-mass index (mass in kilograms divided by the square of the height in meters), HDL high-density lipoprotein, ALT alanine aminotransferase, AST aspartate aminotransferase, HR hazard ratio, and CI confidence interval. Viral Load is measured in RNA copies per milliliter and CD4 count in cells per cubic millimeter. HDL, Triglycerides, and Bilirubin are measured in milligrams per deciliter. AST, ALT, and Amylase are measured in units per liter. Lactate is measured in millimoles per liter. Hazard ratios based on a Cox proportional-hazards model and adjusted for the confounding variables are presented.

Supplementary Table 30. Hazard of Incident Diabetes in the Truven Marketscan Database among Propensity-Score Matched Participants*.

Variable	Adjusted HR (95% CI)
**NRTI use (per year)**	0.926 (0.857-0.999)
**NNRTI use (per year)**	0.837 (0.742-0.945)
**PI use (per year)**	0.728 (0.623-0.852)
**INSTI use (per year)**	0.886 (0.703-1.116)
**Index Age**	0.983 (0.951-1.016)
**Sex: Male**	1.101 (0.939-1.291)
**Charlson index**	1.169 (1.146-1.194)
**Index year**	0.867 (0.841-0.893)
**Depression**	0.861 (0.707-1.048)
**Systemic hypertension**	1.187 (1.006-1.399)
**Hyperglyceridemia**	1.142 (0.969-1.345)
**Stroke**	0.735 (0.581-0.930)
**Polycystic ovarian syndrome**	1.044 (0.429-2.545)
**Gestational diabetes**	0 (0-4.74E+119)
**Acanthosis nigricans**	0.584 (0.426-0.801)
**Hepatitis C**	0.828 (0.679-1.010)
**Osteoarthritis**	0.793 (0.668-0.942)
**Rheumatoid arthritis**	1.271 (0.914-1.766)
**Psoriatic arthritis**	1.066 (0.719-1.580)
**Family history of diabetes**	0.947 (0.423-2.121)
**Fluoroquinolone use**	0.908 (0.724-1.140)
**Corticosteroid use**	0.919 (0.718-1.177)
**Antihypertensive medication use**	1.224 (1.038-1.443)
**Lipid lowering medication use**	1.360 (1.094-1.689)

* The propensity-score-matched cohort included 4,343 patients in the NRTI-exposed group and 4,343 patients in the NRTI-non-exposed group. NRTI denotes nucleoside reverse-transcriptase inhibitor, NNRTI non-nucleoside reverse-transcriptase inhibitor, PI protease inhibitor, INSTI integrase strand transfer inhibitor, HR hazard ratio, and CI confidence interval. Hazard ratios based on a Cox proportional-hazards model and adjusted for the confounding variables are presented.

Supplementary Table 31. Hazard of Incident Diabetes in the PearlDiver Database among Propensity-Score Matched Participants*.

Variable		Adjusted HR (95% CI)
**NRTI Use (Per year of exposure)**		0.830 (0.719-0.958)
**NNRTI Use (Per year of exposure)**		0.945 (0.901-0.992)
**PI Use (Per year of exposure)**		0.941 (0.857-1.033)
**INSTI Use (Per year of exposure)**		0.800 (0.594-1.077)
**Sex (reference=Female)**	**Male**	0.984 (0.838-1.156)
**Race (reference=White)**	**Black**	0.856 (0.420-1.291)
	**Hispanic**	0.962 (0.381-1.544)
**Charlson comorbidity index**		1.009 (0.986-1.032)
**Tobacco use**		1.034 (0.877-1.218)
**Index age (Per year)**		0.971 (0.931-1.013)
**Index year (Per year)**		1.101 (1.034-1.171)
**Viral Load (reference=missing)**	**0-500**	1.094 (0.846-1.415)
	**501-1000**	0.467 (0.197-1.105)
	**>1000**	1.858 (0.238-14.50)
**CD4 count (reference=missing)**	**0-50**	0.879 (0.535-1.444)
	**51-250**	1.300 (0.916-1.845)
	**>250**	1.024 (0.771-1.361)
**BMI (reference=normal)**	**30-40**	0.939 (0.792-1.112)
	**>40**	1.051 (0.850-1.299)
**Systemic hypertension**		0.910 (0.657-1.262)
**Pure hypercholesterolemia**		0.891 (0.756-1.050)
**Hyperglyceridemia**		0.855 (0.635-1.151)
**Depression**		1.009 (0.862-1.180)
**Ischemic heart disease**		1.110 (0.942-1.309)
**Other heart disease**		0.952 (0.805-1.126)
**Stroke**		0.920 (0.748-1.131)
**Polycystic ovarian syndrome**		1.143 (0.289-4.526)
**Gestational diabetes**		1.405 (0.420-4.594)
**Acanthosis nigricans**		2.082 (0.595-7.285)
**Hepatitis C**		1.235 (0.971-1.572)
**Osteoarthritis**		0.827 (0.712-0.962)
**Rheumatoid arthritis**		0.954 (0.736-1.237)
**Psoriatic arthritis**		0.834 (0.416-1.675)
**HDL (Baseline=Missing)**	**40-59**	1.045 (0.876-1.247)
	**<40**	1.038 (0.863-1.250)
	**>=60**	1.037 (0.862-1.246)
**Triglycerides (Baseline=Missing)**	**150-199**	1.035 (0.874-1.226)
	**200-499**	0.954 (0.799-1.140)

	**<150**	0.892 (0.713-1.116)
	**>=500**	1.127 (0.827-1.537)
**ALT (Baseline=Missing)**	**40-79**	1.164 (0.958-1.414)
	**80-120**	0.997 (0.766-1.297)
	**<40**	1.033 (0.673-1.587)
	**>120**	0.859 (0.617-1.196)
**AST (Baseline=Missing)**	**40-79**	0.871 (0.711-1.066)
	**80-120**	1.020 (0.770-1.353)
	**<40**	0.884 (0.578-1.352)
	**>120**	0.987 (0.697-1.397)
**Bilirubin (Baseline=Missing)**	**2-3**	1.031 (0.746-1.423)
	**<2**	0.981 (0.662-1.454)
	**>3**	0.933 (0.641-1.360)
**Amylase (Baseline=Missing)**	**<=140**	1.068 (0.875-1.305)
	**>140**	0.864 (0.518-1.441)
**Family history of diabetes**		1.019 (0.739-1.406)
**Fluoroquinolone use**		0.905 (0.778-1.053)
**Antipsychotic medication use**		1.173 (0.978-1.406)
**Corticosteroid use**		0.850 (0.730-0.991)
**Antihypertensive medication use**		1.026 (0.810-1.299)
**Lipid lowering medication use**		1.086 (0.916-1.287)

[*] The propensity-score-matched cohort included 2,153 patients in the NRTI-exposed group and 2,153 patients in the NRTI-non-exposed group. NRTI denotes nucleoside reverse-transcriptase inhibitor, NNRTI non-nucleoside reverse-transcriptase inhibitor, PI protease inhibitor, INSTI integrase strand transfer inhibitor, BMI body-mass index (mass in kilograms divided by the square of the height in meters), HDL high-density lipoprotein, ALT alanine aminotransferase, AST aspartate aminotransferase, HR hazard ratio, and CI confidence interval. Viral Load is measured in RNA copies per milliliter and CD4 count in cells per cubic millimeter. HDL, Triglycerides, and Bilirubin are measured in milligrams per deciliter. AST, ALT, and Amylase are measured in units per liter. Hazard ratios based on a Cox proportional-hazards model and adjusted for the confounding variables are presented.

Supplementary Methods

Databases

Veterans Health Administration

Data Source

This study used claims from the VA system from January 2000-July 2017. Data were extracted from the VA Informatics and Computing Infrastructure (VINCI). Data include all inpatient, outpatient, and pharmacy claims. Study approval and waiver of Health Insurance Portability and Accountability Act authorization were provided by the Dorn VAMC Institutional Review Board. The completeness, utility, accuracy, validity, and access methods are described on the VA website: [https://www.virec.research.va.gov].

Participants and Sample Selection

Patients were included in the analysis if they met these criteria: had at least 2 medical claims for HIV/AIDS or hepatitis B during the study. Individuals with pre-existing type 2 diabetes (≥1 medical claim prior to diagnosis of HIV or hepatitis B) were excluded. For Falsification Test 1, individuals with pre-existing appendicitis (≥1 medical claim prior to diagnosis of HIV or hepatitis B) were excluded. For Falsification Test 2, individuals with pre-existing hernia (≥1 medical claim prior to diagnosis of HIV or hepatitis B) were excluded.

Exposure to Different Classes of Medications to Treat HIV/AIDS

Individuals were classified as receiving NRTI, NNRTI, PI, or INSTI medications if they filled ≥1 outpatient pharmacy prescription for these medications (listed in Supplementary Table 1) as extracted from the outpatient VA pharmacy data. Use of combination medications, e.g. Efavirenz/Emtricitabine/Tenofovir, were counted as taking medications from each class. The VA

pharmacy data has information on the dispensed date and the days of supply allowing us to quantify the total number of days in which a supply was available during the study period.

Dependent Variable

Time to initial diagnosis of type 2 diabetes, appendicitis, or hernia during the follow-up period, as identified by the ICD-9-CM codes corresponding to type 2 diabetes (250.xx), appendicitis (540-542), and hernia (550-553), and ICD-10-CM codes corresponding to type 2 diabetes (E11), appendicitis (K35-K37), and hernia (K40-K44) was the dependent variable for this analysis. The index date was the date of the first medical claim for HIV or hepatitis B. Patients with a medical claim for type 2 diabetes prior to index HIV or hepatitis B diagnosis were excluded. Patients were followed until the first date of 1) type 2 diabetes, 2) death, 3) July 1, 2017. For Falsification Test 1, patients with a medical claim for appendicitis prior to index HIV or hepatitis B diagnosis were excluded, and were followed until the first date of 1) appendicitis, 2) death, 3) July 1, 2017. For Falsification Test 2, patients with a medical claim for hernia prior to index HIV or hepatitis B diagnosis were excluded, and were followed until the first date of 1) hernia, 2) death, 3) July 1, 2017.

Key Predictor Variables

Key predictors were NRTI, NNRTI, PI, and INSTI usage. For each of these drug classes, medication use was summarized as a categorical covariate (ever used / never used) or as a time dependent covariate measuring the cumulative days supplied over the study time.

Analysis

To analyze the risk of type 2 diabetes, appendicitis, or hernia between those exposed to NRTIs and those not exposed to NRTI medications, we fit adjusted Cox proportional hazard models. The adjusted model included as covariates demographic variables, comorbidities, use of other

medications, and laboratory test values listed in Supplementary Table 5. Statistical tests were two-sided. P values < 0.05 were considered statistically significant.

Period-specific Hazard Ratios

In the main analysis, we estimated the hazard ratio comparing NRTI exposure to non-exposure over the entire study period. However, hazard ratios may change over time and conclusions drawn from a single overall hazard ratio may be dependent on the length of follow-up[1]. To investigate possible changes in the hazard ratio over time, we used different follow-up periods (1, 2 5, and 10 years) in the Veterans Health Administration (VHA) database. We developed four different outcome variables denoting patients who developed type 2 diabetes within the above respective follow-up periods. Patients who did not develop type 2 diabetes within the follow-up period are considered censored and their survival time was set as the end of the respective follow-up period. We fit four Cox proportional hazards models, one for each follow-up period. All Cox models included the same covariates as the primary model. We present the hazard ratios and 95% confidence intervals.

Survival curves

Unadjusted and adjusted survival curves for the VHA database were computed among those exposed and unexposed to NRTIs over the study period. We used the R package IPWsurvival, which constructs the adjusted survival curves using inverse probability weights[2]. We used a logistic regression model to estimate the probability of being in the NRTI-exposed group. Variables included in the logistic regression model were the same as those used in the propensity score matching model (listed in the section below). Individual contributions to the survival curve were weighed by inverse probabilities treatment weights, i.e., 1/P(NRTI exposure) or 1/[1 – P(NRTI exposure)].

Competing risk of mortality analysis

We used the Vital Status File (VSF), which includes the date of death for Veterans who utilized services from the VHA and the Veterans Benefits Administration (VBA). Information in the VSF emanates from multiple sources including the Social Security Administration Death Master File, VBA's Beneficiary Identification Records Locator Subsystem Death File, and the VHA Medicare Vital Status File[3]. The relationship between NRTI use and type 2 diabetes was analyzed in a competing risks framework, accounting for death as a competing risk to type 2 diabetes development. A multivariable Fine and Gray's competing risks models[4] was fitted with an indicator of death, type 2 diabetes or censored. The competing risks models-derived subdistribution hazard ratio is the ratio of the instantaneous risk at time t of having type 2 diabetes between two groups given no type 2 diabetes up to time t. Those who have died are considered event free in terms of type 2 diabetes. The subdistribution hazard can be interpreted similarly to the hazard ratios of the Cox model.

Truven

Data Source

Data on patients with employer-based health insurance was obtained from the Truven Marketscan Commercial Claims & Medicare Supplemental Database (IBM), comprises de-identified outpatient, inpatient, and pharmaceutical claims corresponding to approximately 164 million privately insured individuals between January 1, 2006 and December 31, 2017, originating from more than 150 large employer-sponsored health insurance plans from all fifty U.S. states. All data within the Truven database are Health Insurance Portability and

Accountability Act-compliant and were thus deemed exempt from institutional review board's approval by the University of Virginia Institutional Review Board. The completeness, utility, accuracy, validity, and access methods are described on the Truven Marketscan website: [https://www.ibm.com/products/marketscan-research-databases].

Participants and Sample Selection

Patients were included in the analysis if they met these criteria: had at least 2 medical claims for HIV/AIDS or hepatitis B during the study. Individuals with pre-existing type 2 diabetes (≥1 medical claim prior to diagnosis of HIV or hepatitis B) were excluded. For Falsification Test 1, individuals with pre-existing appendicitis (≥1 medical claim prior to diagnosis of HIV or hepatitis B) were excluded. For Falsification Test 2, individuals with pre-existing hernia (≥1 medical claim prior to diagnosis of HIV or hepatitis B) were excluded.

Exposure to Different Classes of Medications to Treat HIV/AIDS

Individuals were classified as receiving NRTI, NNRTI, PI, or INSTI medications if they filled ≥1 outpatient pharmacy prescription for these medications (listed in Supplementary Table 1) as identified based on American Hospital Formulary Service drug codes. Use of combination medications, e.g. Efavirenz/Emtricitabine/Tenofovir, were counted as taking medications from each class.

Dependent Variable

Time to initial diagnosis of type 2 diabetes, appendicitis, or hernia during the follow-up period, as identified by the ICD-9-CM codes corresponding to type 2 diabetes (250.xx), appendicitis (540-542), and hernia (550-553), and ICD-10-CM codes corresponding to type 2 diabetes (E11), appendicitis (K35-K37), and hernia (K40-K44) was the dependent variable for this analysis. Patients with a medical claim for type 2 diabetes prior to index HIV or hepatitis B diagnosis

were excluded. Patients were followed until the first date of 1) type 2 diabetes, 2) death, 3) December 31, 2017. For Falsification Test 1, patients with a medical claim for appendicitis prior to index HIV or hepatitis B diagnosis were excluded, and were followed until the first date of 1) appendicitis, 2) death, 3) December 31, 2017. For Falsification Test 2, patients with a medical claim for hernia prior to index HIV or hepatitis B diagnosis were excluded, and were followed until the first date of 1) hernia, 2) death, 3) December 31, 2017.

Key Predictor Variables

Key predictors were NRTI, NNRTI, PI, and INSTI usage. For each of these drug classes, medication use was summarized as a categorical covariate (ever used / never used) or as a time dependent covariate measuring the cumulative days supplied over the study time.

Analysis

To analyze the risk of type 2 diabetes, appendicitis, or hernia between those exposed to NRTIs and those not exposed to NRTI medications, we fit adjusted Cox proportional hazard models. The adjusted model included as covariates demographic variables, comorbidities, and use of other medications, listed in Supplementary Table 7. Statistical tests were two-sided. P values < 0.05 were considered statistically significant.

PearlDiver

Data Source

Data on patients with commercial health insurance was obtained from the PearlDiver Patient Records Database (Colorado Springs), which captures health care claims, medication usage, and laboratory data for persons in the Humana network between 2007 and the first quarter of 2017. All data within the PearlDiver database are Health Insurance Portability and Accountability Act-

compliant and were thus deemed exempt from institutional review board's approval by the University of Virginia Institutional Review Board. The completeness, utility, accuracy, validity, and access methods are described on the PearlDiver website: [http://www.pearldiverinc.com/researchinfo.html].

Participants and Sample Selection

Patients were included in the analysis if they met these criteria: had continuous enrollment in the medical plan for at least 3 years and had at least 2 medical claims of HIV/AIDS or Hepatitis B during those first 3 years they were in the plan. Individuals with pre-existing type 2 diabetes (1 or more medical claims prior to diagnosis of HIV or hepatitis B) were excluded. For Falsification Test 1, individuals with pre-existing appendicitis (1 or more medical claims prior to diagnosis of HIV or hepatitis B) were excluded. For Falsification Test 2, individuals with pre-existing hernia (1 or more medical claims prior to diagnosis of HIV or hepatitis B) were excluded.

Exposure to Different Classes of Medications to Treat HIV/AIDS

Individuals were classified as receiving NRTI, NNRTIs, PIs, and INSTIs if they filled 1 or more outpatient pharmacy prescription for these medications (listed in Supplementary Table 1) as identified based on American Hospital Formulary Service drug codes. Use of combination medications were counted as taking medications from each class. The databases contain information on the number of months for which an enrollee filled a prescription for a given medication, thereby enabling us to quantify the amount each beneficiary had taken during their time in the plan.

Dependent Variable

Time to initial diagnosis of type 2 diabetes, appendicitis, or hernia during the follow-up period, as identified by the ICD-9-CM codes corresponding to type 2 diabetes (250.xx), appendicitis

(540-542), and hernia (550-553), and ICD-10-CM codes corresponding to type 2 diabetes (E11), appendicitis (K35-K37), and hernia (K40-K44), was the dependent variable for this analysis. Enrollees with type 2 diabetes, appendicitis, or hernia prior to index HIV or hepatitis B diagnosis were excluded for the corresponding analyses. Observation of beneficiaries was right censored at the end of plan enrollment.

Key Predictor Variables

Key predictors were NRTI, NNRTI, PI, and INSTI usage. Medication use was summarized as a categorical covariate (ever use / never use) or as time-dependent covariates measuring the cumulative months supplied over the study time.

Analysis

To analyze the risk of type 2 diabetes, appendicitis, or hernia between those exposed to NRTIs and those not exposed to NRTI medications, we fit adjusted Cox proportional hazard models. The adjusted model included as covariates demographic variables, comorbidities, use of other medications, and laboratory test values listed in Supplementary Table 9. Statistical tests were two-sided. P values < 0.05 were considered statistically significant.

Medicare & Clinformatics

Data Sources

Data on Medicare enrollees were from a random 20% sample of Medicare beneficiaries with Parts A, B, and D coverage. Data were available from enrollees between 2008 and 2016. Data on patients with commercial health insurance was obtained from the Clinformatics DataMart database (OptumInsight), which captures health care claims for persons in a large nationwide managed care network between 2001 and 2016. The completeness, utility, accuracy, validity, and

access methods are described on the Medicare [https://data.medicare.gov/] and Clinformatics [https://www.optum.com/business/solutions/life-sciences/explore-data/advanced-analytics/claims-data.html] websites.

Both databases include information on diagnoses as identified using ICD-9-CM billing codes, service dates, locations of services, types of healthcare providers caring for the patient, and patient demographic information including age, sex, and race/ethnicity. All patients in these two data sources were fully enrolled in the pharmacy plans, and data on all outpatient medication prescriptions filled were recorded. These data sources have been used extensively to study trends in utilization and outcomes of patients with ocular and non-ocular diseases. The University of Michigan Institutional Review Board approved this study.

Participants and Sample Selection

Patients were included in the analysis if they met these criteria: had continuous enrollment in the medical plan for at least 3 years and had at least 2 medical claims for HIV/AIDS or hepatitis B during those first 3 years they were in the plan (the "lookback period"). Individuals with pre-existing type 2 diabetes (1 or more medical claims during lookback) were excluded, as were individuals with missing age, sex or race/ethnicity information. Medicare or Clinformatics enrollees whose sex or race was unknown were excluded. Clinformatics enrollees whose education, household net worth, or residency was unknown were excluded. For Falsification Test 1, individuals with pre-existing appendicitis (1 or more medical claims during lookback) were excluded, as were individuals with missing age, sex or race/ethnicity information. For Falsification Test 2, individuals with pre-existing hernia (1 or more medical claims during lookback) were excluded, as were individuals with missing age, sex or race/ethnicity information. In addition, commercially insured enrollees younger than 55 years old at eligibility

were excluded as were Medicare enrollees younger than age 65 and those in Medicare Advantage plans.

Exposure to Different Classes of Medications to Treat HIV/AIDS

Individuals were classified as receiving NRTI, NNRTIs, PIs, and INSTIs if they filled 1 or more outpatient pharmacy prescription for these medications (listed in Supplementary Table 1) as identified based on American Hospital Formulary Service drug codes. Use of combination medications were counted as taking medications from each class. The databases contain information on the number of days for which an enrollee filled a prescription for a given medication, thereby enabling us to quantify the amount each beneficiary had taken during their time in the plan.

Dependent Variable

Time to initial diagnosis of type 2 diabetes, appendicitis, or hernia during the follow-up period, as identified by the ICD-9-CM codes corresponding to type 2 diabetes (250.xx), appendicitis (540-542), and hernia (550-553), and ICD-10-CM codes corresponding to type 2 diabetes (E11), appendicitis (K35-K37), and hernia (K40-K44), was the dependent variable for this analysis. Enrollees with type 2 diabetes, appendicitis, or hernia during lookback were excluded for their respective studies. Observation of beneficiaries was right censored at the end of plan enrollment.

Key Predictor Variables

The key predictor variables in our models were NRTI, NNRTI, PI, and INSTI use. Medication use changed over time and was summarized both as a categorical covariate (prior use / no prior use) and as a covariate measuring the number of days' supply of the medication of interest during the previous 3 year moving time window.

Analyses

Participant characteristics were summarized by means and standard deviations for continuous variables and frequencies and percentages for categorical variables. Ever/never or time-dependent proportional hazards regression models were used to estimate the hazard for developing type 2 diabetes, appendicitis, or hernia during follow-up associated with use of NRTIs, NNRTIs, PIs, and INSTIs. We fit a base model using these four predictors. We also fit models adjusting for demographic variables, comorbidities, and use of other medications listed in Supplementary Tables 11 and 13. Using additional variables available in Clinformatics DataMart but not Medicare, we fit another model adjusting also for education level, household net worth, and residency (urban, rural). Model diagnostics were performed to check model fit. We used SAS software, version 9.4 (SAS Institute) to perform all statistical analyses. Statistical tests were two-sided. P values < 0.05 were considered statistically significant.

Supplementary References

1 Hernan, M. A. The hazards of hazard ratios. *Epidemiology* **21**, 13-15, doi:10.1097/EDE.0b013e3181c1ea43 (2010).

2 Cole, S. R. & Hernan, M. A. Adjusted survival curves with inverse probability weights. *Comput Methods Programs Biomed* **75**, 45-49, doi:10.1016/j.cmpb.2003.10.004 (2004).

3 Sohn, M. W., Arnold, N., Maynard, C. & Hynes, D. M. Accuracy and completeness of mortality data in the Department of Veterans Affairs. *Popul Health Metr* **4**, 2, doi:10.1186/1478-7954-4-2 (2006).
