# Supplementary material for: Repurposing anti-inflammasome NRTIs for improving insulin sensitivity and reducing type 2 diabetes development
Source: Nat Commun. 2020 Sep 23;11:4737. doi: 10.1038/s41467-020-18528-z (PMC7511405; doi:10.1038/s41467-020-18528-z)
Supplement: Supplementary file 2 — Reporting Summary [file 41467_2020_18528_MOESM2_ESM.pdf]

nature research

Corresponding author(s): Ambati

Last updated by author(s): Aug 20, 2020

Reporting Summary

Nature Research wishes to improve the reproducibility of the work that we publish. This form provides structure for consistency and transparency in reporting. For further information on Nature Research policies, see our Editorial Policies and the Editorial Policy Checklist.

Statistics

For all statistical analyses, confirm that the following items are present in the figure legend, table legend, main text, or Methods section.

n/a	Confirmed	
☐	☒	The exact sample size (*n*) for each experimental group/condition, given as a discrete number and unit of measurement
☐	☒	A statement on whether measurements were taken from distinct samples or whether the same sample was measured repeatedly
☐	☒	The statistical test(s) used AND whether they are one- or two-sided<br>*Only common tests should be described solely by name; describe more complex techniques in the Methods section.*
☐	☒	A description of all covariates tested
☐	☒	A description of any assumptions or corrections, such as tests of normality and adjustment for multiple comparisons
☐	☒	A full description of the statistical parameters including central tendency (e.g. means) or other basic estimates (e.g. regression coefficient) AND variation (e.g. standard deviation) or associated estimates of uncertainty (e.g. confidence intervals)
☐	☒	For null hypothesis testing, the test statistic (e.g. *F*, *t*, *r*) with confidence intervals, effect sizes, degrees of freedom and *P* value noted<br>*Give P values as exact values whenever suitable.*
☐	☒	For Bayesian analysis, information on the choice of priors and Markov chain Monte Carlo settings
☒	☐	For hierarchical and complex designs, identification of the appropriate level for tests and full reporting of outcomes
☒	☐	Estimates of effect sizes (e.g. Cohen's *d*, Pearson's *r*), indicating how they were calculated

Our web collection on statistics for biologists contains articles on many of the points above.

Software and code

Policy information about availability of computer code

Data collection	All images were obtained by microscopy (model SP-5, Leica or A1R Nikon confocal microscope system, Nikon), Gen5 3.00 (microtiter plate colorimetric assays), LICOR Image studio
Data analysis	ImageJ Fiji version 2.1.0/1.53c, Microsoft Excel version 16.35, Graphpad Prism version 8.3.0, SAS version 9.4, R version 3.6.1

For manuscripts utilizing custom algorithms or software that are central to the research but not yet described in published literature, software must be made available to editors and reviewers. We strongly encourage code deposition in a community repository (e.g. GitHub). See the Nature Research guidelines for submitting code & software for further information.

Data

Policy information about availability of data

All manuscripts must include a data availability statement. This statement should provide the following information, where applicable:

- Accession codes, unique identifiers, or web links for publicly available datasets
- A list of figures that have associated raw data
- A description of any restrictions on data availability

The experimental data that support this study are available from the corresponding author upon reasonable request. Analyses of the Veterans Health Administration Database were performed using data within the US Department of Veterans Affairs secure research environment, the VA Informatics and Computing Infrastructure (VINCI). The other health insurance datasets are subject to licensing agreements and privacy restrictions. All relevant data outputs are within the paper and its supplemental information. Researchers interested in accessing aggregate and individual data are encouraged to make direct enquiries to the corresponding author and should note they may also need to approach Truven MarketScan, PearlDriver, Clinformatics, and the Centers for Medicare & Medicaid Services for access to data from these sources.

Field-specific reporting

Please select the one below that is the best fit for your research. If you are not sure, read the appropriate sections before making your selection.

☒ Life sciences ☐ Behavioural & social sciences ☐ Ecological, evolutionary & environmental sciences

For a reference copy of the document with all sections, see nature.com/documents/nr-reporting-summary-flat.pdf

Life sciences study design

All studies must disclose on these points even when the disclosure is negative.

Sample size	Sample sizes were selected based on power analysis alpha=5%; 1-beta= 80%, such that we could detect a minimum of 50% change assuming a sample SD based on Bayesian inference.
Data exclusions	No data were excluded, however some animals (<5%) were excluded from the experiments due to technical challenges in the procedures such as animal death due to anesthesia complications, etc.
Replication	The experimental findings were reliably reproduced through repeated experiments (3-10 times). Details in Figure Legends.
Randomization	Mice were randomly distributed into treatment groups within each genotype. For databases, covariates were controlled by multivariate Cox regression and also by propensity score matching.
Blinding	To eliminate operator bias, the researchers performing the procedures and analyses were blinded to the identity of the experimental groups.

Reporting for specific materials, systems and methods

We require information from authors about some types of materials, experimental systems and methods used in many studies. Here, indicate whether each material, system or method listed is relevant to your study. If you are not sure if a list item applies to your research, read the appropriate section before selecting a response.

Materials & experimental systems

n/a	Involved in the study	
☐	☒	Antibodies
☒	☐	Eukaryotic cell lines
☒	☐	Palaeontology and archaeology
☐	☒	Animals and other organisms
☒	☐	Human research participants
☒	☐	Clinical data
☒	☐	Dual use research of concern

Methods

n/a	Involved in the study	
☒	☐	ChIP-seq
☒	☐	Flow cytometry
☒	☐	MRI-based neuroimaging

Antibodies

Antibodies used	Mouse anti-human phospho-specific AKT, Ser473 (#12694, Cell Signaling Technology; 1:1000); rabbit anti-mouse AKT (pan), 11E7 (#4685, Cell Signaling Technology; 1:1000); rabbit anti-human DICER1 A301-936A (Bethyl Laboratories; 1:1000); rat anti-mouse IL-18 (Clone 39-3F, #D046-3, MBL International; 1:1000); anti-mouse β-actin (8H10D10) (#3700, Cell Signaling Technology; 1:1000; for loading control assessment).
Validation	1- According to the manufacturer, validation of Phospho-Akt (Ser473) mouse mAb was conducted by Western blot analysis of extracts from various cell lines, untreated or treated with Human Platelet-Derived Growth Factor AA (hPDGF-AA) and by purified recombinant phospho-Akt1, phospho-Akt2 and phospho-Akt3. "Phospho-Akt (Ser473) (D9W9U) Mouse mAb detects endogenous levels of Akt1 only when phosphorylated at Ser473, Akt2 only when phosphorylated at Ser474 and Akt3 only when phosphorylated at Ser473." Species Reactivity: Human, Mouse, Rat, Monkey. Applications: Western Blotting, Immunoprecipitation. Citations: PMID: 31579406, PMID: 31572504.<br>2- According to the manufacturer, validation of rabbit anti-mouse AKT (pan), 11E7 was conducted by Western blot analysis of recombinant Akt1, Akt2 and Akt3 proteins, and extracts from HeLa, C2C12, C6 and COS cells. "This antibody does not cross-react with other related proteins." Species Reactivity: Human, Mouse, Rat, Monkey. Applications: Western blotting, Immunoprecipitation, Immunohistochemistry (Paraffin), Immunofluorescence (Immunocytochemistry), Flow Cytometry. Citations: PMID: 32377744, PMID: 32377751.<br>3- DICER1: The epitope recognized by A301-936A maps to a region between residue 600 and 650 of human double-stranded RNA-specific endoribonuclease; dicer1 using the numbering given in entry NP_085124.2 (GeneID 23405). Species Reactivity: Human, Mouse. Applications: Western blotting, Immunoprecipitation. Citations: PMID: 32484548, PMID: 32220961.<br>4- According to the manufacturer, validation of rat anti-mouse IL-18 was confirmed by western blot of recombinant mouse IL-18. Reactivity: mouse. Applications: Western blotting. Citations: PMID: 17400729, PMID: 11390444.<br>5- According to the manufacturer, "β-Actin (8H10D10) Mouse mAb detects endogenous levels of total β-actin protein." Reactivity:

	Human, Mouse, Rat, Hamster, Monkey, Dog. Applications: Western Blotting, Immunohistochemistry (Paraffin), Immunofluorescence (Immunocytochemistry), Flow Cytometry. Citations: PMID: 32174193, PMID: 32483433.

Animals and other organisms

Policy information about studies involving animals; ARRIVE guidelines recommended for reporting animal research

Laboratory animals	Male, 12-week-old C57BL/6J mice
Wild animals	No wild animals were used in the study.
Field-collected samples	No field collected samples were used in the study.
Ethics oversight	Approvals or exemptions obtained from the IRBs of the Dorn VAMC, University of Michigan, and University of Virginia.

Note that full information on the approval of the study protocol must also be provided in the manuscript.
